# Mechanical stretch induced transcriptomic profiles in cardiac myocytes

**DOI:** 10.1038/s41598-018-23042-w

**Published:** 2018-03-16

**Authors:** Jaana Rysä, Heikki Tokola, Heikki Ruskoaho

**Affiliations:** 1 School of Pharmacy, University of Eastern Finland, Kuopio, Finland; 2 Research Unit of Biomedicine, Pharmacology and Toxicology, University of Oulu, Oulu, Finland; 3 Department of Pathology, Cancer Research and Translational Medicine Research Unit, University of Oulu and Oulu University Hospital, Oulu, Finland; 4 Drug Research Program, Division of Pharmacology and Pharmacotherapy, University of Helsinki, Helsinki, Finland

## Abstract

Mechanical forces are able to activate hypertrophic growth of cardiomyocytes in the overloaded myocardium. However, the transcriptional profiles triggered by mechanical stretch in cardiac myocytes are not fully understood. Here, we performed the first genome-wide time series study of gene expression changes in stretched cultured neonatal rat ventricular myocytes (NRVM)s, resulting in 205, 579, 737, 621, and 1542 differentially expressed (>2-fold, P < 0.05) genes in response to 1, 4, 12, 24, and 48 hours of cyclic mechanical stretch. We used Ingenuity Pathway Analysis to predict functional pathways and upstream regulators of differentially expressed genes in order to identify regulatory networks that may lead to mechanical stretch induced hypertrophic growth of cardiomyocytes. We also performed micro (miRNA) expression profiling of stretched NRVMs, and identified that a total of 8 and 87 miRNAs were significantly (P < 0.05) altered by 1–12 and 24–48 hours of mechanical stretch, respectively. Finally, through integration of miRNA and mRNA data, we predicted the miRNAs that regulate mRNAs potentially leading to the hypertrophic growth induced by mechanical stretch. These analyses predicted nuclear factor-like 2 (Nrf2) and interferon regulatory transcription factors as well as the let-7 family of miRNAs as playing roles in the regulation of stretch-regulated genes in cardiomyocytes.

## Introduction

Cardiac hypertrophy provides an adaptive mechanism to maintain cardiac output in response to increased workload, such as occurs in diseases such as chronic hypertension or myocardial infarction. In the early stages of pathological hypertrophy, changes in cardiac structure compensate for the increased load, whereas in later stages, excessive hypertrophic growth results in decompensation and heart failure^1,2^. As cardiac myocytes are generally considered to be terminally differentiated cells^3^, cardiac hypertrophy has been considered to involve an increase in the size of individual cardiomyocytes. Hypertrophy is associated with alterations in the structure of the heart referred to as myocardial remodeling; in the late-phase, this includes increased extracellular matrix (ECM) deposition, perturbations of cellular calcium homeostasis and an increased rate of cardiac myocyte apoptosis^4,5^.

Hypertrophic growth of cardiac myocytes is triggered by mechanical stimuli as well as the neurohumoral activation produced by cardiac overload. At the level of individual cardiac myocytes, the hypertrophic response is characterized not only by an increase in cell size, but also by an accumulation in the total protein, enhanced sarcomeric reorganization and complex changes in cardiac gene expression^1,6^. The early genetic response to hypertrophic stimulation involves the rapid and transient activation of the so-called immediate-early genes such as proto-oncogenes (*c-fos, c-myc, c-jun* and *Egr-1*) independently of new protein synthesis^7^ and a heat shock protein-70^8^. For example, the activation of c-fos mRNA levels in response to mechanical stretch in cardiomyocytes has been shown to occur within 15 minutes and then to decline to undetectable levels after 4 h^9^. Translated protein products of the c-fos and c-jun genes play an important role as transcriptional regulators of cardiac gene expression^10^. This is followed by an upregulation of fetal genes, initially B-type natriuretic peptide (BNP), and later reactivation of atrial natriuretic peptide (ANP) gene as well as β-myosin heavy chain (β-MHC) and skeletal muscle α-actin (skαA) contractile protein isoforms^11^.

Both cellular components and extracellular structures have been shown to contribute to the transfer of mechanical stretch signals into the nucleus. These mechanotransduction elements include ECM, cell-cell adhesions, ECM–sensing receptors including integrins, cytoskeletal filaments, nuclear scaffolds, and ion channels. Subsequently, mechanical stretch is able to activate a complex network of parallel downstream signal transduction pathways as well as elevating autocrine production and the release of growth factors, resulting in *de novo* synthesis of immediate response genes and total protein synthesis^12,13^. It is still poorly understood whether stretch itself is the direct trigger for the growth process, and furthermore, the mechanisms of altered gene expression are still largely unknown.

Numerous animal models, especially genetically modified animals, have been widely used to elucidate the molecular mechanisms of cardiac hypertrophy^14,15^. Moreover, many *in vitro* and *ex vivo* models have been developed for investigating the different components of hemodynamic overload^16^. However, cell culturing is virtually the only approach that makes it possible to study individually the different cell types and molecular characteristics of distinctive components of cardiac overload (e.g. mechanical stretch, neurohumoral factors) under experimentally controlled conditions for periods long enough to detect changes in gene expression and morphology^17^. The majority of cell culture studies have been conducted with primary neonatal rat ventricular myocytes (NRVMs)^17–19^. Although gene expression changes are crucial for the development of cardiac hypertrophy, virtually nothing is known about genome-wide gene expression response of stretched cardiac myocytes. As far as we are aware, the gene expression patterns have been examined only at one timepoint, 24 hours after the start of biaxial stretching in NRVMs^20^ or cyclic stretching in cardiac myocytes^21^. In addition, there is one report of a DNA microarray analysis of stretched neonatal rat cardiac fibroblasts in response to 24 hours of stretching^21^.

Here, we characterized the comprehensive time course of mechanical stretch activated hypertrophic gene expression response in a well-established *in vitro* model of cultured NRVMs. In addition, we analyzed the functional interaction networks among differentially expressed genes by using Ingenuity pathway analysis (IPA). Finally, we identified mechanical stretch regulated microRNAs (miRNAs) in cardiomyocytes and elucidated their stretch regulated target genes. We believe that this is the first study to fully cover the genome-wide transcriptomic changes in cultured cardiac myocytes from the triggering of the mechanical stretch and to the subsequent stretching of the cells for two days.

## Results

### Mechanical stretch induced cardiac hypertrophic gene program

We validated the *in vitro* mechanical stretch model of cultured NRVMs by measuring gene expression levels of immediately early gene *c-fos*, as well as BNP and ANP mRNA levels, which represent genetic hallmarks of the stretch induced cardiac hypertrophic program^22–24^. In agreement with previous studies, the highest induction of c-fos mRNA levels was detected after one hour of mechanical stretch (2.4-fold, P < 0.01) (Supplementary Table S1), whereas cyclic mechanical stretch elevated ANP and BNP mRNA levels significantly after 1 hour, peaking at 24 to 48 hours (5.6 ± 1.0-fold, P < 0.01 and 3.6 ± 0.4, P < 0.001, respectively) (Supplementary Table S2).

### Enhanced kinase activation by mechanical stretch in NRVMs

Previous studies have shown that mechanical stretch activates protein kinase C (PKC)-pathway and all three main mitogen activated protein kinase (MAPK) pathways; extracellular signal regulated kinase (ERK), c-Jun N-terminal kinase (JNK) and p38 MAPK pathways^8,13,24,25^. Consistently with previous studies, mechanical stretch evoked significant activation of p38 MAPK, ERK and JNK–pathways as shown by western blot analyses (Supplementary Fig. 1A). The ratio of phosphorylated to total p38 MAPK increased transiently, peaking at 4 hours (2.4-fold, P < 0.001) and returning near to basal levels by 24 hours (Supplementary Fig. 1A). When compared with p38 MAPK, the activation of ERK and JNK was more rapid, peaking already at 15 to 30 minutes (3.3-fold, P < 0.01, and 3.1-fold, P < 0.001, respectively) (Supplementary Fig. 1B,C).

### Differentially expressed genes after mechanical stretching

We evaluated gene expression profiles at 1, 4, 12, 24, and 48 hours after the start of cyclic mechanical stretching in order to identify those genes that were regulated by mechanical load in cardiac myocytes *in vitro*. We confirmed selected microarray results by their comparison with the corresponding mRNA levels obtained by Northern blot analysis or real-time quantitative RT-PCR. As shown in Supplementary Tables S1 and S2, similar fold changes in mRNA levels were observed as measured by both DNA microarray and Northern/RT-PCR.

**Figure 1:**
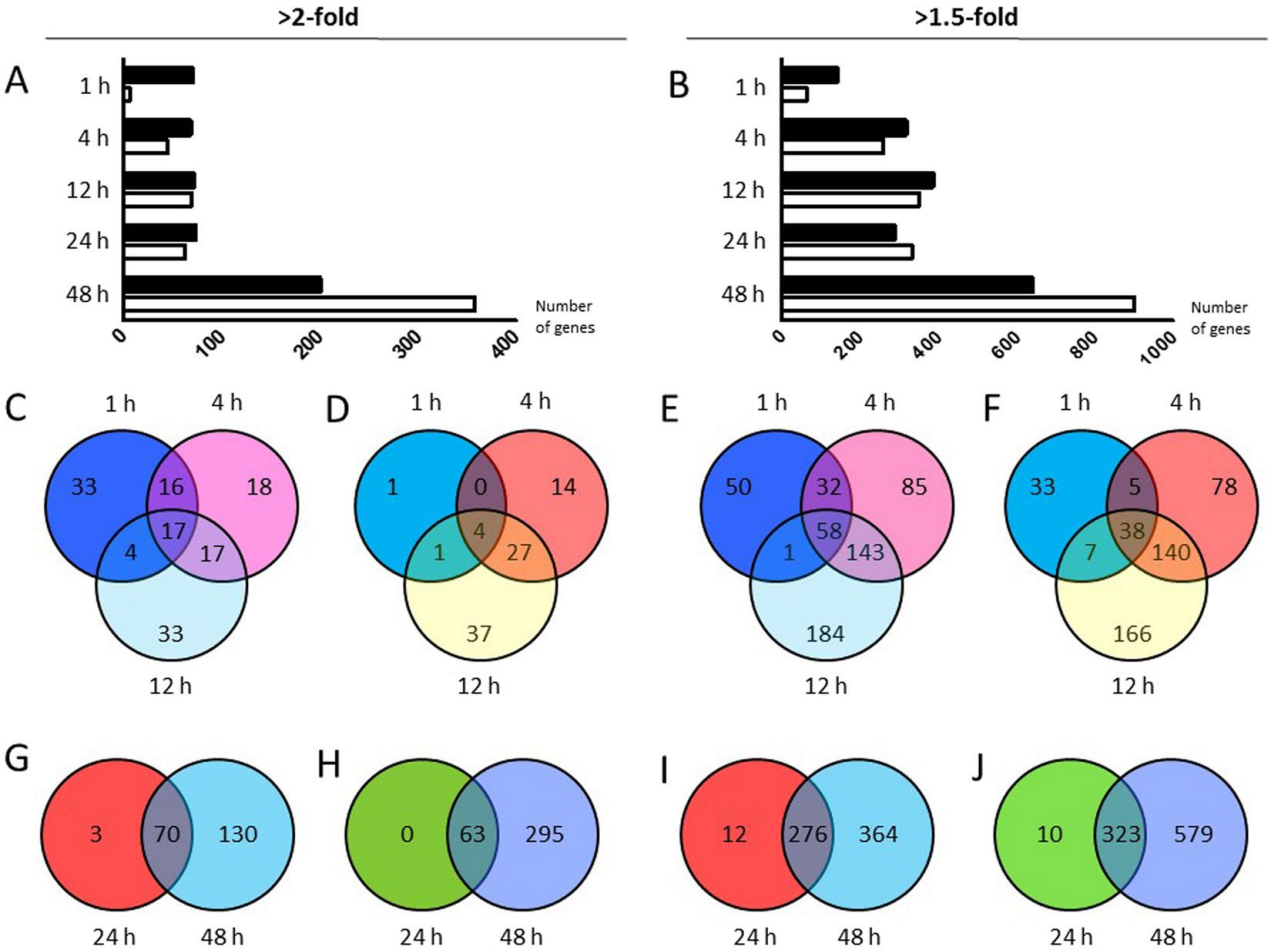
Differentially expressed genes in stretched neonatal rat ventricular myocytes. (**A**,**B**) Numbers of differentially expressed genes in response to 1–48 hours of cyclic mechanical stretch in cultured cardiac myocytes with (**A**) 2-fold difference and (**B**) 1.5 fold difference. Black columns, upregulated genes; white columns downregulated genes, n = 5 in all groups. (**C**–**I**) Venn diagrams indicate the overlap of the genes that were significantly (**C**) upregulated (>2-fold) or (**D**) downregulated (<2-fold) and (**E**) upregulated (>1.5-fold) or (**F**) downregulated (<1.5-fold) after 1, 4 or 12 hours of stretch as compared to controls. A Venn diagram indicating the overlap of the genes that were significantly (**G**) upregulated (>2-fold) or (**H**) downregulated (<2-fold) and (**I**) upregulated (>1.5-fold) or (**J**) downregulated (<1.5-fold) after 24 or 48 hours of stretch compared to controls.

The gene expression profiling of stretched NRVMs identified altogether 76, 113, 140, 136, and 558 different transcripts that were differentially expressed (2-fold, p < 0.05) in response to mechanical stretching of 1, 4, 12, 24, and 48 hours, respectively (Fig. 1A). The numbers of differentially expressed genes were notably higher when we compared 1.5-fold differences in gene expression levels of stretched myocytes with those of unstretched control cells (Fig. 1B). According to these selection criteria, our analysis identified 205, 579, 737, 621, and 1542 differentially expressed genes in response to 1, 4, 12, 24, and 48 hours of mechanical stretching, respectively. After one hour, more genes were upregulated than were downregulated, which was followed by approximately similar numbers of up- and down-regulated genes in response to 4, 12, and 24 hours of stretching. Instead, at 48 hours after the start of the stretch, more genes were repressed than upregulated.

**Figure 2:**
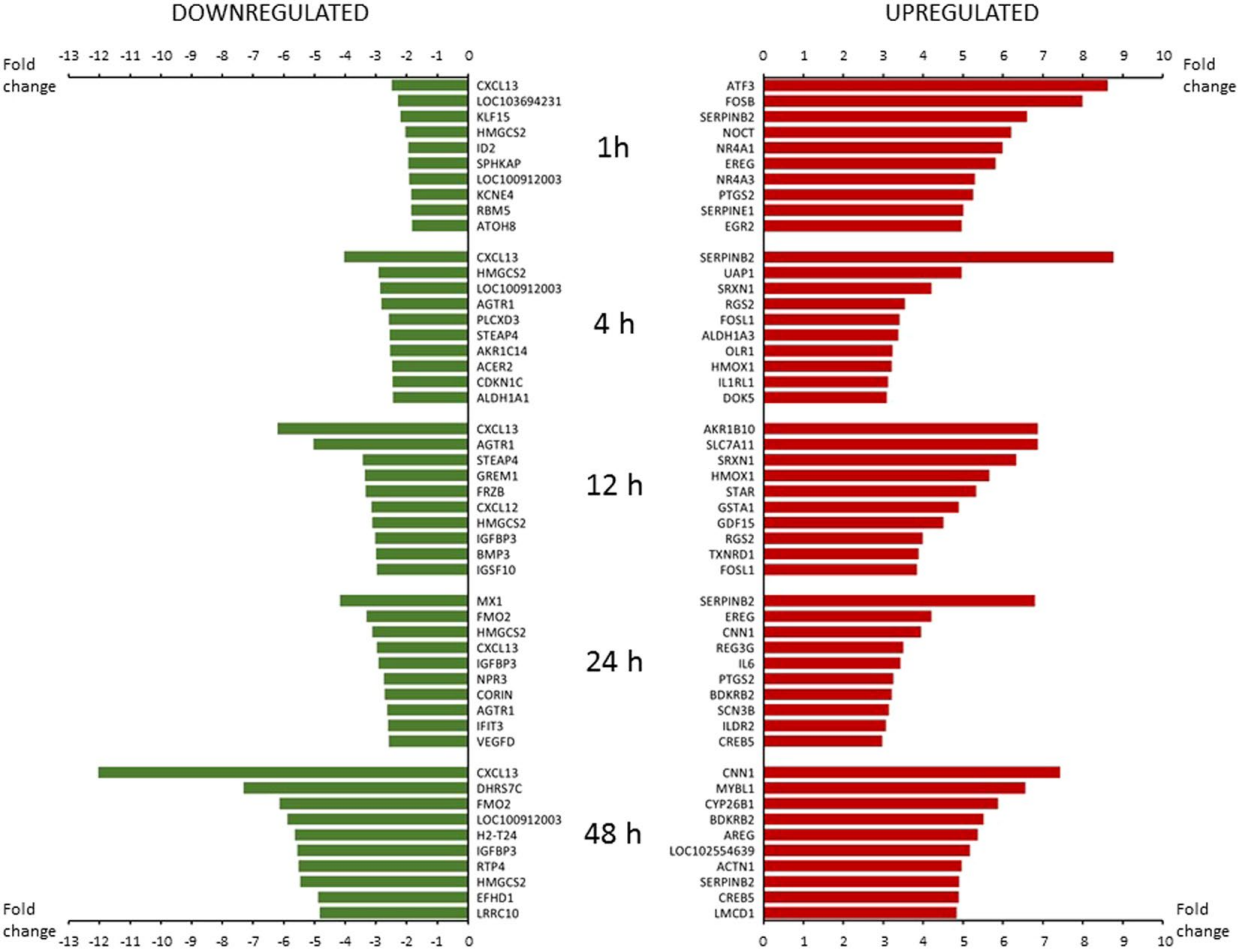
The ten most up- and down-regulated genes in stretched myocytes at 1–48 hours after the initiation of the mechanical stretch. Fold change indicates statistically significant (P < 0.05) difference in gene expression between stretched and unstretched neonatal rat ventricular myocytes. Green columns, downregulated genes; red columns upregulated genes.

Venn diagrams were produced to describe the overlap of significantly up- or down-regulated genes in stretched myocytes (Fig. 1C–J). At earlier timepoints (1–12 hours), more timepoint specific differences were observed in the expression patterns of differentially expressed genes (Fig. 1C–F), whereas in response to 24 and 48 hours of stretch (Fig. 1G–J), the majority of differentially expressed genes in 24 hours were up- or down-regulated also after 48 hours of mechanical stretching. These trends in the amount of differentially expressed genes were essentially similar whether we evaluated 2-fold (Fig. 1C,D,G,H) or 1.5-fold (Fig. 1E,F,I,J) differences in expression levels of stretched cells in comparison to unstretched control cells. The ten most up- and down-regulated genes in stretched myocytes at each time point are shown in Fig. 2. The total lists of differentially expressed genes by mechanical stretch in NRVMs are provided in Supplementary Dataset 1 and the data with normalized values in Supplementary Dataset 2.

### Functional and pathway analysis of differentially expressed genes

**Figure 3:**
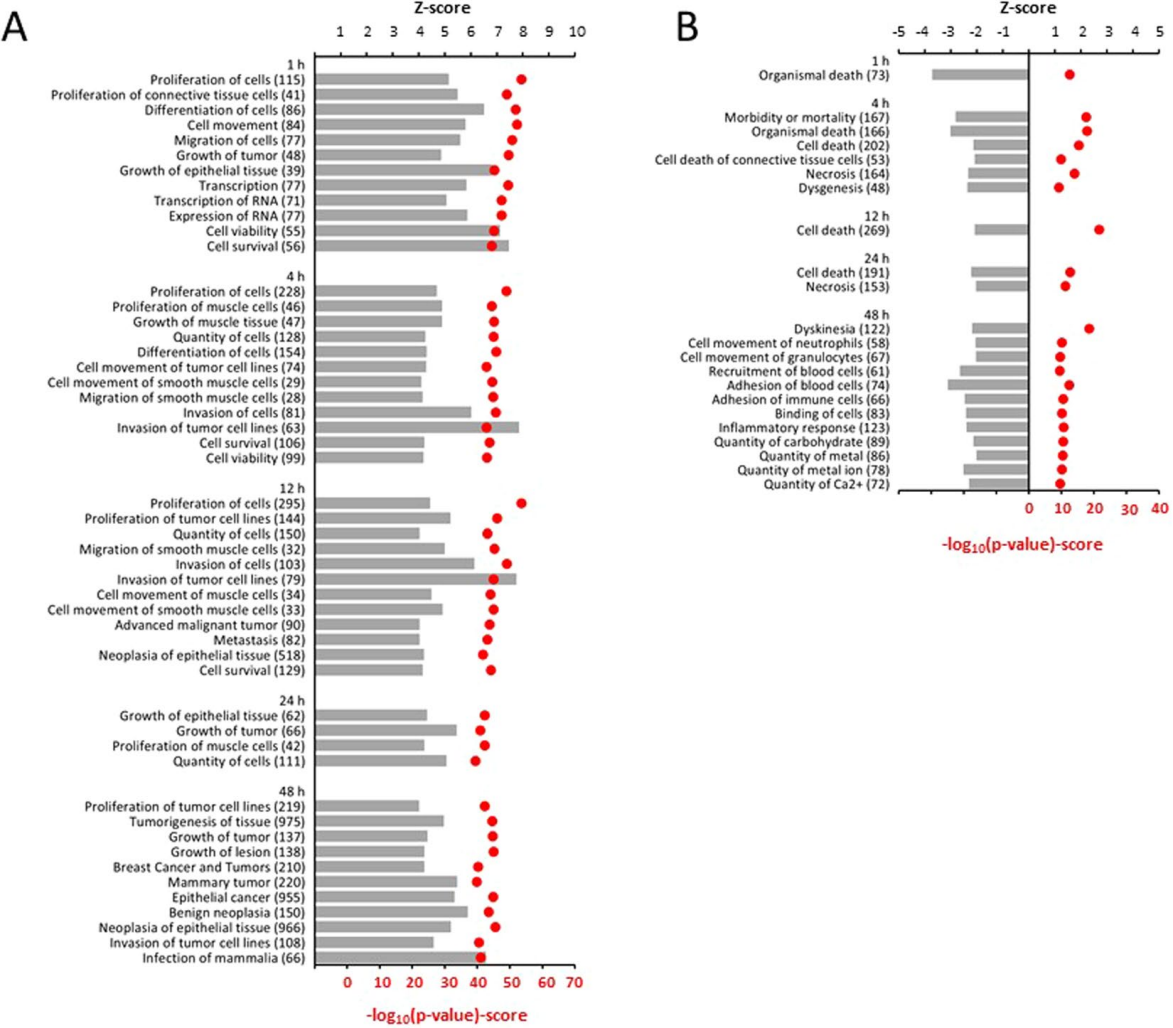
Functional analysis of differentially expressed genes in stretched neonatal rat ventricular myocytes. Differentially expressed genes (1.5-fold upregulation or downregulation compared to controls; P < 0.05) were used in the functional analysis in the IPA-software. A positive or negative z-score value (columns) indicates that a function would be predicted to be either increased (**A**) or decreased (**B**) in stretched myocytes. Only those functional annotations that obtained a significant regulation z-score (>2) are presented. The p-value (red dots) reflects the likelihood that the association between a set of genes in our dataset and a related biological function would be significant [p-value < 0.05 (i.e., −log10 ≥ 1.3), Fisher’s Exact test]. A maximum 10 genes per activation/inactivation-route and P < 1.00E-9 are shown per timepoint. Numbers of differentially expressed genes in each functional class are shown in parentheses.

By using the datasets of differentially expressed genes in response to mechanical stretch at each timepoint, it was possible to conduct an IPA functional analysis and generate predictions for significantly increased or decreased (z-score >2 or <−2, respectively) activity in various cellular processes. The most evidently altered biological functions are summarized in Fig. 3. In response to 1–24 hours of mechanical stretch, more biological pathways were activated than inactivated, and the activated functions were mostly related to cellular growth and proliferation whereas cell death and necrosis were among the functions predicted as being decreased by IPA-analysis (Fig. 3A–D). At 48 hours, more biological pathways were inactivated than activated, although pathways related to cell growth remained active (Fig. 3E). All significantly altered biological pathways are described in Supplementary Dataset 3.

### Canonical pathways

**Figure 4:**
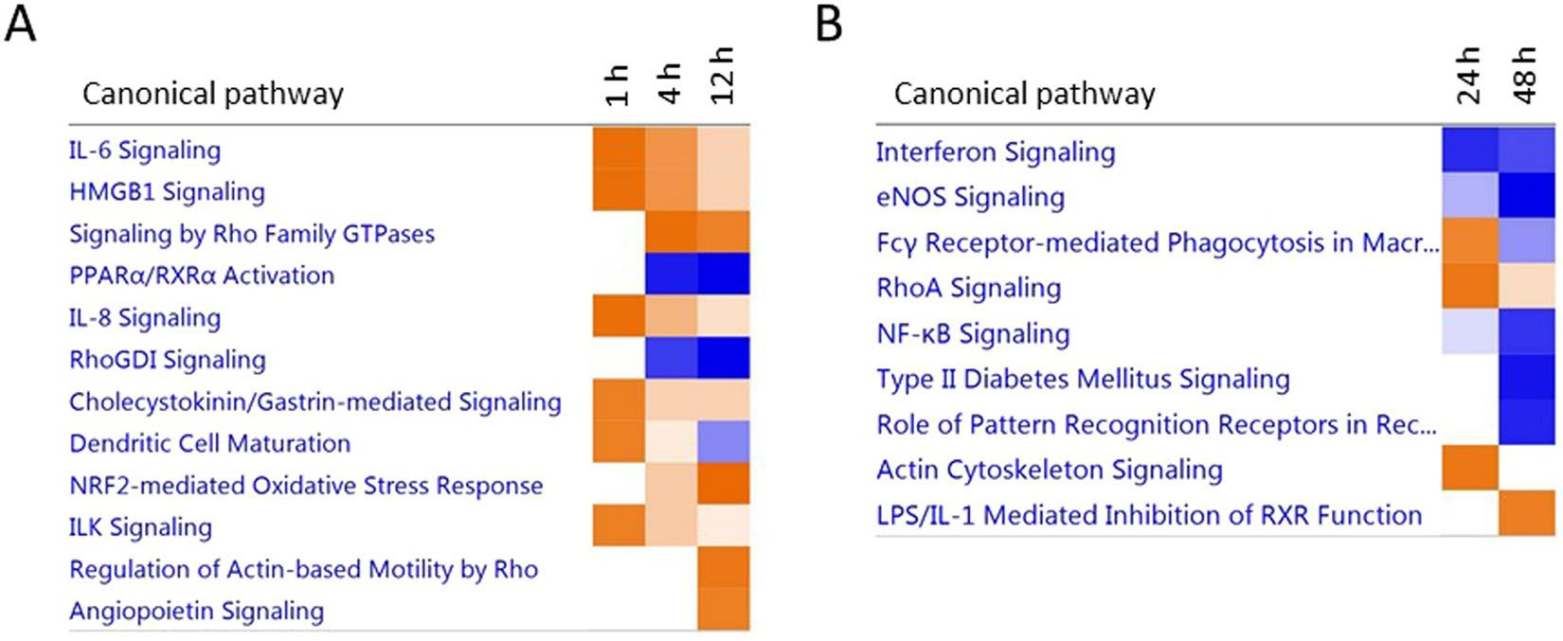
Significant canonical pathways associated with differentially expressed genes in stretched neonatal ventricular myocytes. Ingenuity pathway analysis showing the canonical pathways that were most significant in the data sets of differentially expressed genes after (**A**) 1–12 and (**B**) 24–48 hours of mechanical stretch in cardiac myocytes. The entries that have a −log (p-value) greater than 1.3 and an absolute z-score value greater than 2 are displayed. Colors of the bars in the chart indicate their activation z-scores: orange, overall increase in the activity of the pathway; blue, decrease in activity. The intensity of the color indicates the degree of increase or decrease.

**Figure 5:**
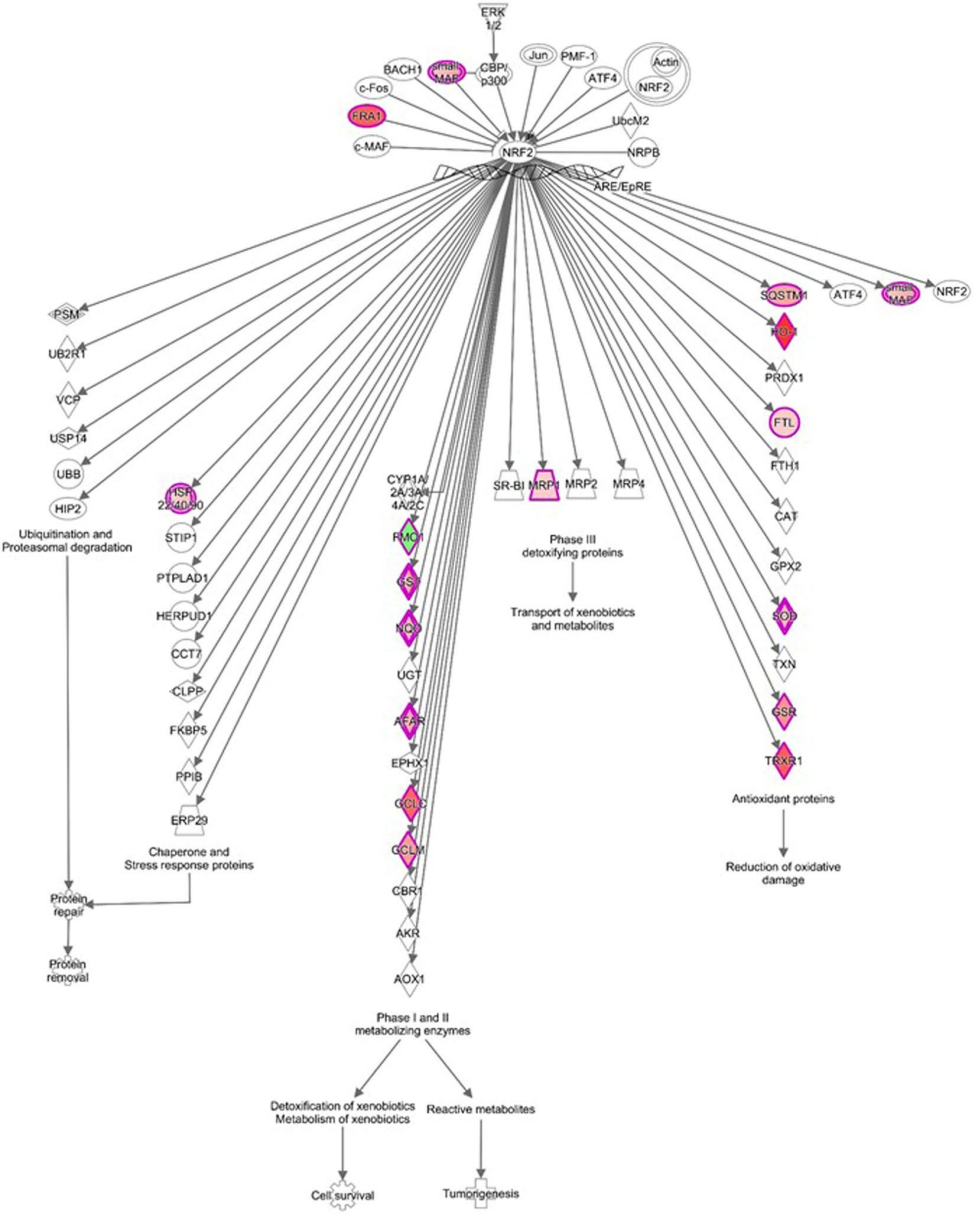
NRF2-mediated oxidative stress response –signaling in the nucleus. Significant canonical pathways associated with differentially expressed genes in response to 12 hours of mechanical stretch in neonatal rat ventricular myocytes are shown. Up- and down-regulated genes are displayed in red and green, respectively. The intensity of the color indicates the degree of up-regulation or downregulation. Genes are represented as various shapes that represent the functional class of the gene product. Full annotations for genes and molecules in this canonical pathway and whole figure of this canonical pathway are provided in Supplementary Datafile 1and Fig. S2, respectively.

Next, specific changes in canonical biological pathways related to differentially expressed genes at each timepoint (Supplementary Dataset 1) were examined (Fig. 4). Our canonical pathway analysis revealed that in the acute phase, all of the significantly modulated pathways, such as the proinflammatory interleukin-1 (IL-6) and IL-8 signaling pathways, had become activated. In the late phase response (48 hours), the majority of signaling pathways were repressed, including the Nf-kB and endothelial nitric oxide synthase (eNOS) signaling pathways. The nuclear factor-like 2 (Nrf2) mediated oxidative stress response signaling was the pathway having the highest activation (a −log [p-value] and an absolute z-score >2) in stretched myocytes during the acute phase (i.e. at 12 hours), whereas in the late phase, it was lipopolysaccharide (LPS)/IL-1 mediated inhibition of retinoid X receptor (RXR) function –signaling (at 48 hours). The transcriptional response regulated by Nrf2 in the nucleus of stretched NRVMs is shown in Fig. 5 with the whole pathway illustrated in Supplementary Fig. 2. LPS/IL-1 mediated inhibition of RXR function –signaling pathway is shown in Supplementary Fig. 3. In addition to known pathobiological pathways, it is likely that other molecular mechanisms are involved in mediating hypertrophic growth of cardiomyocytes. IPA-analysis was used to determine the biological relationships among the differentially expressed genes at each timepoint. The top five molecular networks underlying stretch-activated gene expression changes at each timepoint based on Fisher’s exact test and full annotations for genes and molecules in these networks are shown in Supplementary File 2.

### Upstream analysis

**Figure 6:**
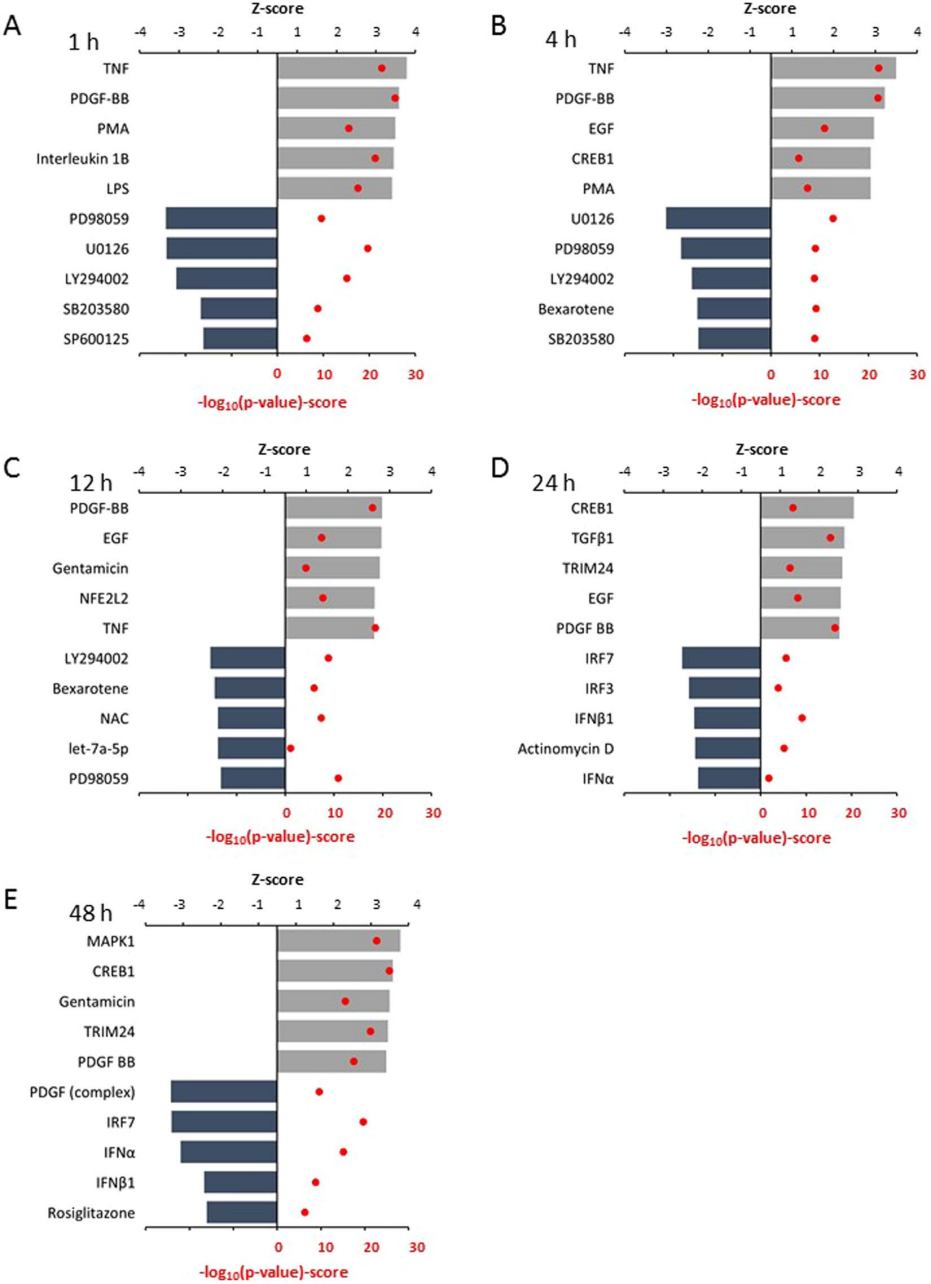
Upstream analysis of mechanical stretch regulated genes. The top five upstream regulators that were predicted to be activated and inhibited in response to (**A**) 1 hour, (**B**) 4 hours (**C**), 12 hours (**D**), 24 hours and (**E**) 48 hours of mechanical stretch in neonatal rat ventricular myocytes are shown. Z-scores ≥2 or ≤−2 indicate that the upstream regulator was predicted to be activated or inhibited, respectively. The p-value (red dots) calculated by a Fisher’s Exact Test was used to determine the significance of the overlap [p-value < 0.05 (i.e., −log10 ≥ 1.3) between the regulator and stretch-responsive genes. Only functional annotations that obtained a significant regulation z-score (>2) are presented. CREB1, cAMP-responsive element binding protein 1; LPS, lipopolysaccharide; EGF, epidermal growth factor; IFNB1, interferon beta 1; IRF3, interferon regulatory factor 3; IRF7, interferon regulatory factor 7; PDGF-BB, platelet-derived growth factor BB-isoform; PMA, phorbol myristate acetate (activator of protein kinase C, PKC); NAC, N-acetyl-L-cysteine; NFE2L2, NRF2 transcription factor; TGFβ1, transforming Growth Factor β1, TNF, tumor necrosis factor; TRIM24, tripartite motif-containing 24. The targets of the inhibitors are LY294002, P13K inhibitor; PD98059, MEK-inhibitor, SB203580, MAPK-inhibitor, SP600125, JNK-inhibitor; U0126, MEK1 and MEK2 inhibitor.

Next we performed an upstream analysis to identify potential upstream regulators that could explain the observed changes in gene expression patterns in stretched myocytes. The top five upstream regulators that were predicted to be activated or inhibited in stretched NRVMs are shown in Fig. 6. In the acute phase, tumor necrosis factor (TNF), epidermal growth factor (EGF) and phorbol myristate acetate (PMA, an activator of PKC) were the most relevant upstream regulators predicted to be in an activated state at several timepoints (Fig. 6A–E). The top upstream regulators predicted to be inhibited at the 1 hour timepoint included five kinase inhibitors (LY294002, Phosphatidylinositol-4,5-bisphosphate 3-kinase [P13K] inhibitor; PD98059, MAPK of ERK kinase [MEK]-inhibitor, SB203580, MAPK-inhibitor, SP600125, JNK-inhibitor; U0126, MEK1 and MEK2 inhibitor), and many of them were among the top predicted inhibited regulators throughout the acute phase (until 12 hours of stretching). In the late phase, several transcription factors were among the predicted upstream regulators: activated regulators included cAMP responsive element binding protein 1 (CREB1) and tripartite Motif-Containing Protein 24 (TRIM24); the inhibited regulators included interferon (IFN) regulatory transcription factor (IRF) family (IRF)7, IRF3, and Peroxisome proliferator-activated receptor gamma coactivator 1α (PPARGC1A) (Fig. 6D–E). All significant upstream regulators at each timepoint are detailed in Supplementary Dataset 4.

### Differentially expressed miRNAs in stretched cardiac myocytes

**Figure 7:**
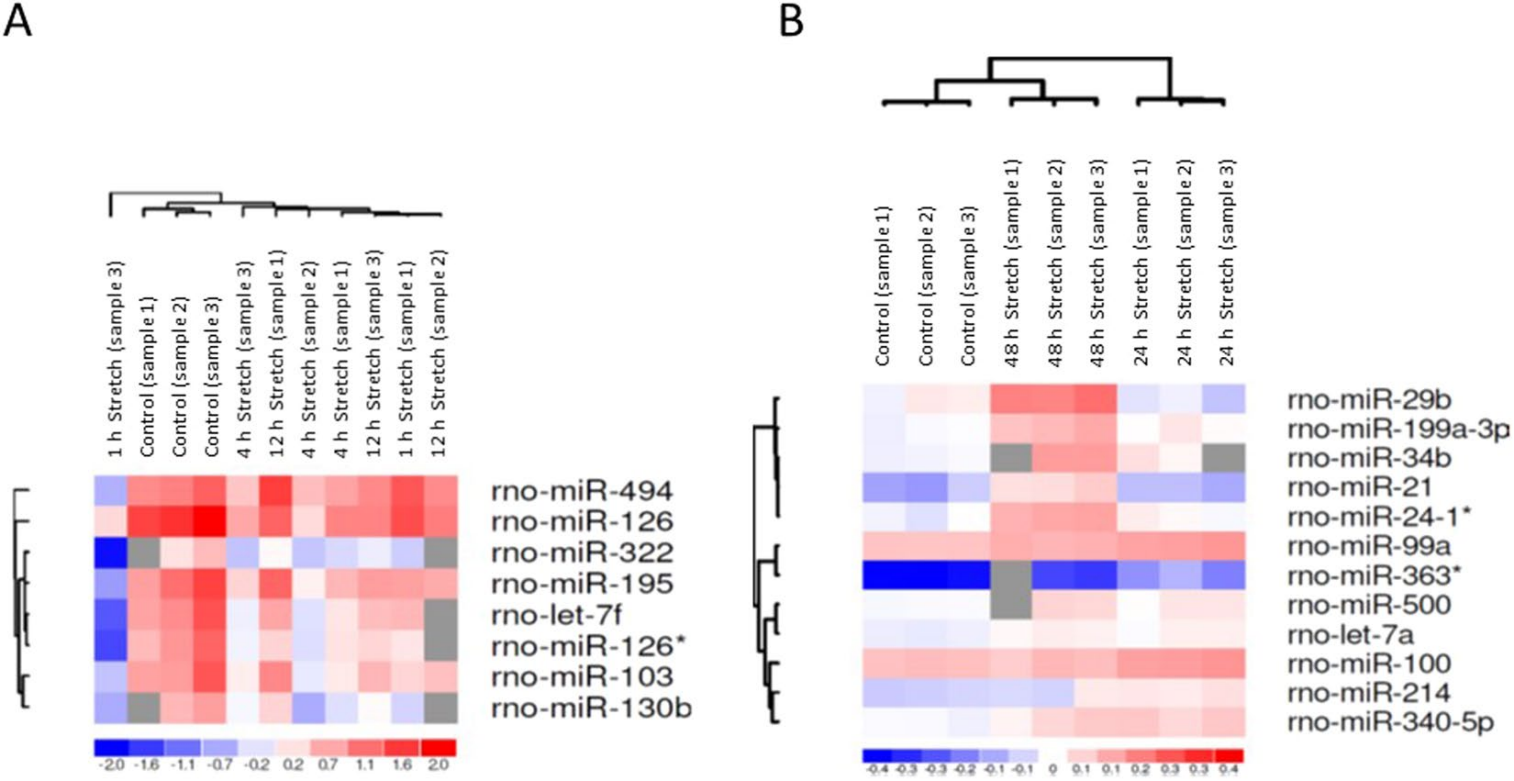
Differentially expressed miRNAs in stretched neonatal rat ventricular myocytes. The heat map diagram shows the results of unsupervised hierarchical clustering of miRNA expression by LNA microarray in cardiac myocytes in response to (**A**) 1, 4 and 12 hrs (P < 0.05) and (**B**) 24 and 48 hrs (P < 0.001) of mechanical stretching (n = 3 in all groups, except n = 2 at 1-hour time point). The clustering was performed on log2(Hy5/Hy3) ratios which passed the filtering criteria by applying a two-tailed t-test between the control and experimental groups. Each row represents a miRNA and each column represents a sample. The miRNA clustering tree is shown on the left. The color scale shown at the bottom illustrates the relative expression level of miRNA across the samples: red color, expression level above mean; blue color, expression lower than the mean; gray color, signal below background.

MicroRNA expression profiling was performed by using commercial miRCURY LNA^TM^ Arrays (v.10.0) to determine differentially expressed miRNA molecules in stretched NRVMs. The analysis indicated that a total of 8 and 87 miRNAs had been significantly altered (P < 0.05) in samples of 1–12 h and 24–48 h, respectively, when compared to unstretched control samples (n = 3 in all groups, except n = 2 in samples from the 12 h timepoint). The heat map diagrams show the results of the two-way unsupervised hierarchical clustering of miRNAs and samples of 1–12 h (P < 0.05; Fig. 7A and Supplementary Dataset 5) and 24–48 h (P < 0.001; Fig. 7B and Supplementary Dataset 5). In response to 1 hour of mechanical stretching, only one miRNA, rno-miR-130b showed differential expression compared to controls (P < 0.05), whereas 8 miRNAs (rno-miR-322, rno-let-7f, rno-miR-103, rno-miR-126, rno-miR-494, rno-miR-126*, rno-miR-130b, rno-miR-195; P < 0.05) were dysregulated in response to 4 hours of stretch (Supplemental Dataset 5). At a later time point i.e. 12 hours of mechanical stretching, only rno-miR-126 exhibited any significantly (P < 0.05) dysregulated expression in response to unstretched cells. In the response to 24 and 48 hours of stretching, 51 and 63 miRNAs were significantly (P < 0.05) differentially expressed in stretched myocytes compared to controls (Supplementary Dataset 5). At 24 hours, five miRNAs (rno-miR-214, rno-miR-99a, rno-miR-363*, rno-miR-100 and rno-miR-340–5p) and at 48 hrs 6 miRNAs (rno-miR-34b, rno-miR-500, rno-miR-24-1*, rno-miR-29b, rno-miR-199a-3p, rno-let-7a) showed the most prominent dysregulation (P < 0.001) (Fig. 7B).

### Integration of miRNA and mRNA expression data

**Figure 8:**
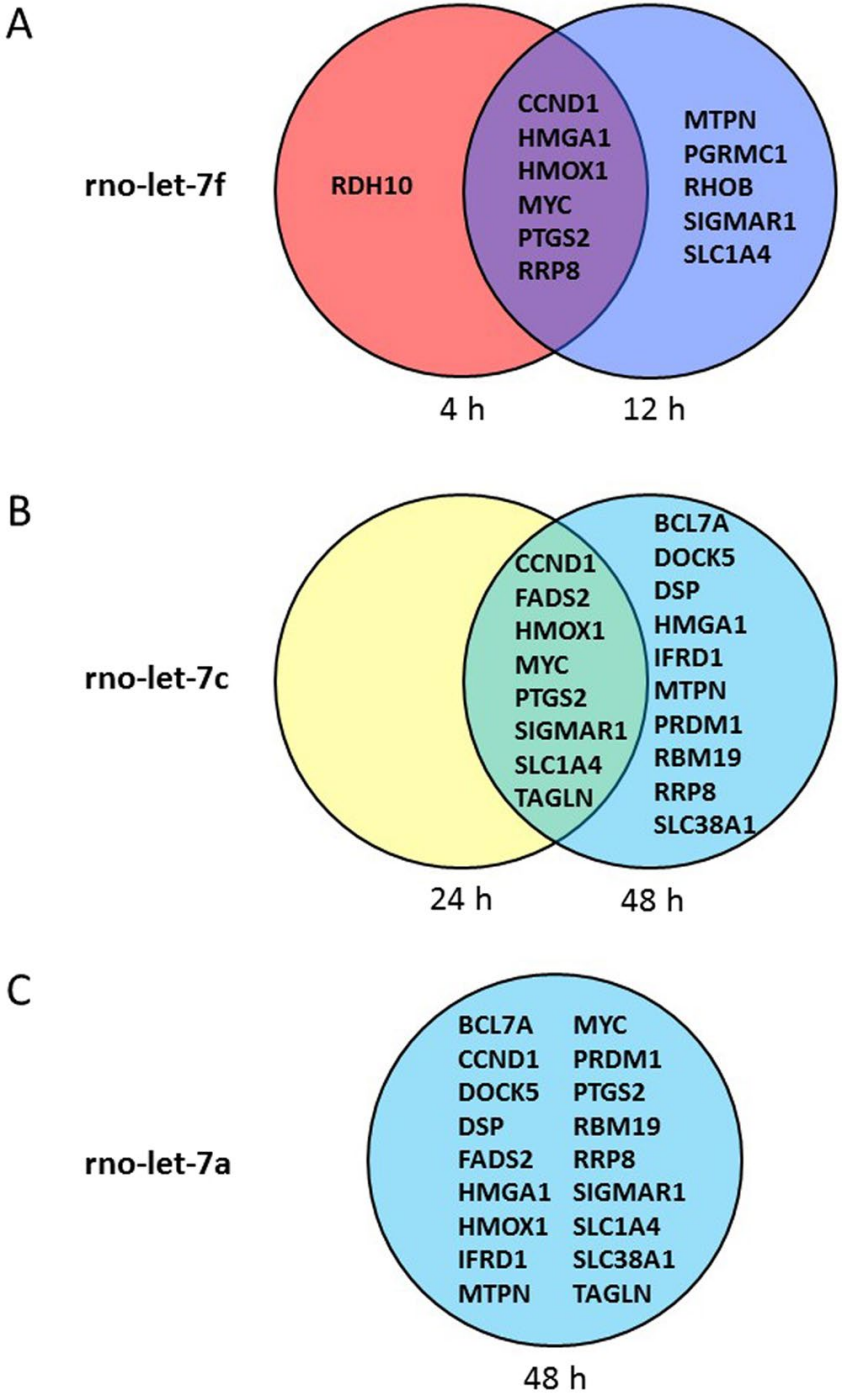
Differentially expressed targets shared by relevant miRNAs. Putative stretch regulated targets for (**A**) let-7f, (**B**) let-7c and (**C**) let-7a were obtained from the IPA-analysis.

In an attempt to characterize potential mRNA-miRNA interactions that regulate mechanical stretch activated hypertrophic response in NRVMs, we performed a correlation analysis between dysregulated miRNAs and the mRNA expression data sets. We used the microRNA Target Filter of IPA software to reveal putative relationships between significantly dysregulated miRNAs in each timepoint (Supplementary Dataset 5) and the corresponding stretch-regulated genes at the same or later timepoints (Supplementary Dataset 1). We applied this approach to all significantly up- or down-regulated miRNAs at each time point as follows: For the only dysregulated miRNA at 1 hours (rno-miR-130b), we searched through the IPA database to identify potential mRNA targets among stretch-regulated genes in response to 1, 4, and 12 hours of stretching. In response to 4 hours of mechanical stretch, rno-miR-322, let-7f, miR-103, miR-126, miR-494, miR-126*, miR-130b and miR-195 were significantly dysregulated (Fig. 7A and Supplementary Dataset 5), so we sought potential mRNA targets for these dysregulated miRNAs among the stretch-regulated genes in 4 and 12 hours timepoints. We conducted a similar comparison for each dysregulated miRNAs at each time point. The results of these analysis are shown in Supplementary Dataset 5. Based on this IPA interrogation analysis, let-7f was considered to have the highest amount of experimentally validated targets among the stretch-activated genes in response to 4 and 12 hours of stretch (7 and 11 mRNAs, respectively) (Fig. 8A). With respect to the miRNAs that were differentially regulated in response to 24 hours of stretch, let-7c had 8 mRNA targets of stretch-regulated genes at 24 hours, and 18 mRNA targets among stretch-regulated genes of 48 hours (Fig. 8B). In contrast, the dysregulated miRNA, let-7a, had 18 target mRNAs among the stretch-regulated genes at the 48 hours timepoint (Fig. 8C).

## Discussion

Cell culture is virtually the only possible approach for elucidating separately the biochemical and molecular characteristics of different components of cardiac overload under experimentally controlled conditions for long enough periods that one can detect changes in morphology and gene expression^17^. The most common *in vitro* model of hypertrophy employs primary cultures of neonatal rat ventricular cardiomyocytes^19^. The use of immortal, transformed cells has limitations since the transformation process changes the basic properties of cardiomyocytes and these might well be very relevant to cardiac biology^26^; the utilization of neonatal mouse ventricular cell cultures is limited because a phenotypic change occurs in which the cells develop autonomous hypertrophy^27^. Here, we performed the first genome-wide time series study of mechanical stretch induced gene expression changes in cardiomyocytes. We also analyzed temporal microRNA expression patterns in stretched NRVMs, and conducted an IPA analysis to predict functional pathways and upstream regulators of differentially expressed genes in order to identify regulatory networks that may lead to mechanical stretch induced hypertrophic growth of cardiomyocytes.

Our analysis identified many novel mechanical stretch responsive genes in cardiomyocytes. For example, we and others have subsequently revealed activating transcription factor 3^28^, bone morphogenetic protein 2^29^, regenerating islet-derived 3γ^30^, dyxin/LMCD1^31,32^, Fn14 (or TNFrsf12a)^33,34^, Wdr12^35^, myeloid leukemia factor-1^36^, Myomasp/LRRC39^37^ and phosphatase and actin regulator 1 (PHACTR1)^38^ to be a part of stretch activated hypertrophic gene program in NRVMs. We validated the reliability of our data by undertaking a DNA microarray analysis; this identified well-established changes of hypertrophic gene expression programme in mechanically stretched cardiomyocytes. In agreement with previous studies^39^, the increased expressions of several immediate early genes such as the fos family (FosB, c-fos, fra-1), JunB, c-myc, Egr-1 and nur77 were seen already after 1 hour of mechanical stretch. Upregulated gene expression of ANP^40^ as well as the two growth factors, TGFβ1 and TGFβ2^13^, observed in this study, are also well recognized transcriptional changes occurring during the development of cardiac hypertrophy. Moreover, there were blunted expressions of muscle creatine kinase (creatine kinase, M-type, CKM) and pyruvate dehydrokinase 4 (PDK4) and myoglobin gene expression, reflecting a switch in cardiac substrate selection from fatty acid to glucose utilization, a phenomenon associated with altered metabolism of the heart in cardiac hypertrophy and failure^41,42^.

There were distinct canonical pathways that were affected in the acute and late phase of mechanical stretch induced hypertrophic response in NRVMs. Nrf2-mediated oxidative stress response –signaling was the most significantly induced canonical pathway in response to mechanical stretch in the acute phase. Nrf2 activation has been shown to reduce myocardial infarct size and cardiac hypertrophy in mice exposed to myocardial infarction and pressure overload, respectively^43,44^. In cultured cells, Nrf2 conferred protection against increased ROS production and growth of NRVMs and cardiac fibroblasts in response to hypertrophic stimuli^44^. Our pathway analysis identified several well-known members of Nrf2 antioxidant signaling in cardiac hypertrophy, such as glutamate cysteine ligase subunits (GCLM and GCLC) and glutathione-disulfide reductase (GSR)^45,46^, but we also detected several Nrf2 targets not previously associated with myocyte hypertrophy, including flavin-containing monooxygenase 1 (FMO1), ferritin light chain (FTL) and aflatoxin B1-aldehyde reductase (AFAR/AKR7A2). In the late phase, the majority of the significantly altered pathways were downregulated including Nf-kB, eNOS and LPS/IL-1 mediated inhibition of RXR function signaling pathways. The interactions between dysregulated genes were further elucidated using networks analysis. Well-established transcriptional regulators of hypertrophic gene response such as MYC proto-oncogene^7^ and TGFβ1^13^ were central regulator molecules in many of these networks.

Our upstream regulator analysis highlighted the importance of mitogen-activated protein kinases as regulators of the hypertrophic gene response. We demonstrated that mechanical stretch activates all three MAPKs (ERK, JNK and p38 MAPK), and in the IPA analysis, MEK, MAPK, JNK, MEK1 and MEK2 inhibitors were found among the predicted inhibited upstream regulators at 1, 4, and 48 hours after the start of mechanical stretch, reflecting a multiple peak activation of these kinases^11^. In addition, TNF and the PKC activator PMA, well-known inducers of hypertrophic gene expression^47,48^, were among the anticipated activated upstream regulators^12,49^. Another novel finding was that two members of IRF family of transcription factors, IRF3 and IRF7 were among the top five predicted inhibited upstream regulators at 24 hours, and IRF7 also at 48 hours. IRF3 and IRF7 have been reported to act as negative regulators of pathological cardiac hypertrophy by inhibiting ERK1/2^50^ and nuclear factor-κB signaling^51^, respectively, although in another study, IRF3 was claimed to regulate cardiac fibrosis but not hypertrophy in mice subjected to angiotensin II-induced hypertension^52^. IRF3 and IRF7 have a role in regulating the transcriptional activity of IFNα and IFNβ^53,54^, i.e. these are other members of the IFN-signaling pathway that were among activated upstream regulators in response to 48 hours of stretching. Binding of IFNβ to its receptor is known to activate the JAK-STAT signaling pathway that has a significant role also in controlling the expression of hypertrophic gene response^11,55^. Interestingly, three other members of the IRF-family, IRF1, IRF4 and IRF9, have also been shown to regulate pathological cardiac hypertrophy^56–58^, making this family of transcription factors a potentially important regulatory mechanism mediating the load responsive genetic response in cardiac hypertrophy.

Our study is the first to identify temporal miRNA expression profiles in response to mechanical stretch in cardiac myocytes. MicroRNA profiling identified several miRNAs that have been previously associated with cardiac hypertrophy such as miR-214, miR-23b, miR-15b, rno-miR-26b, rno-miR-221, rno-miR-222, rno-miR-107^59^, miR-23a, miR-208, rno-miR-133b, miR-19a and mi-r133a^60^. We integrated miRNA and gene expression profiles and by using miRNA target prediction program of IPA software, we could identify miRNAs that were highly correlated with differentially expressed predicted gene targets. In our miRNA-analysis, let-7a, let 7c and let-7f had the greatest amount of experimentally verified target mRNAs among the dysregulated genes supporting the role of Let-7 family of miRNAs as a putatively important regulator of cardiac hypertrophic response through their gene target^59^. At present, little is known about the role of Let-7 family of miRNAs in stretched cardiomyocytes but analyses of mechanosensitive miRNAs associated with muscular dystrophies indicated that the let-7 family was dysregulated in mice with muscular dystrophies with myositis (mdm-mice)^61^.

Although we characterized the mechanical stretch regulated gene response in cardiomyocytes, the *in vivo* mechanical load affects also other cell types present in the heart e.g. connective tissue cells and vascular cells. The important role of MAPKs converting mechanically induced signals into physiological responses has been widely established in several cell types, including fibroblasts^62^, endothelial cells^63^ and vascular smooth muscle cells^64^. Similarly, the role of proto-oncogenes in the stretch-response has been characterized in several cell types in addition to cardiac myocytes^64–66^. With respect to the novel potential regulators identifed in our study, Nrf2 has been shown to exert a protective role against mechanical stretch induced apoptosis in mouse fibroblasts^67^, and mechanical stretch was reported to induce IRF1 binding activity in vascular smooth muscle cells^68^. In conclusion, in addition to cell-type specific factors, there seem to be conserved mechanisms of mechanotransduction.

There are certain limitations to our study. We used neonatal ventricular myocytes that were isolated from neonatal pups of both sexes. It is noteworthy that although the primary cultures derived from an adult rat heart would reflect better the situation in the mature heart, the limitation is that these adult cultured cells either lose their morphology or they lose their ability to undergo spontaneous contractions, when cultured under conditions where the rod-shaped morphology is retained^69,70^. In addition, cardiomyocytes differentiated from human induced pluripotent stem cells (hiPSCs) are relatively immature^71^ but when it becomes possible to culture human cells with a mature adult cardiomyocyte phenotype, it would be interesting to reproduce these stretch experiments in conjunction with next generation sequencing techniques since this would provide a more comprehensive and unbiased analysis of both coding and non-coding RNAs. Recently, Trexler *et al*. (2017) observed sex-specific differences in gene expression along with functional differences in isolated adult rat ventricular myocytes (ARVMs)^72^. Although not analyzed in this study, the mechanical stretch induced gene response most probably occurs in a sexually dimorphic manner. Furthermore, we did not confirm the miRNA profiling results with another method and all the effects on miRNAs on gene targets are only predicted; the validation of miRNA-mRNA regulation in stretched myocytes will have to be the subject of future work. Finally, all the results reflect the situation in isolated cardiomyocytes and do not take into account the contribution of other cell types in the development of cardiac hypertrophy.

In conclusion, our study reveals temporal gene and miRNA expression profiles in response to mechanical stretch in cardiac myocytes and represents a valuable resource of transcriptional responses. In addition, the analysis of regulatory pathways as well as miRNA-mRNA interactions predict that Nrf2 and IRF transcription factors as well as the let-7 family of miRNAs are playing roles in the regulation of mechanical stretch induced gene expression response in cardiomyocytes and these may help in elucidating the genes and regulatory pathways underlying cardiac hypertrophy.

## Methods

### Cell culture

Neonatal rat ventricular myocytes were prepared from 2–4 day old Sprague-Dawley rats from the colony of the Center for Experimental Animals in the University of Oulu as previously described^73^. The experimental design was approved by the Animal Use and Care Committee of the University of Oulu or the National Animal Experiment Board and all experiments were performed in accordance with relevant guidelines and regulations. Briefly, after digestion of ventricular tissue with collagenase (2 mg/ml), the cell suspension was pre-plated for 30–45 minutes in Dulbecco’s modification of Eagle’s medium/Ham’s F12 medium (DMEM/F12) containing 10% of fetal bovine serum (FBS). The non-attached myocyte-enriched cell fraction was plated at a density of 2 × 10^5^/cm^2^ on flexible bottomed collagen I-coated 6-well elastomere plates (Bioflex, Flexcell International Corporation, Hillsborough, NC, USA.), and cultured overnight with DMEM/F12 10% FBS, and thereafter in complete serum free medium (CSFM). The cells were exposed for 15 minutes to 48 hours to cyclic mechanical stretch as previously described^73^. Stretch was introduced onto the cells by applying a cyclic vacuum suction under the flexible bottomed plates by computer-controlled equipment (Flexercell Strain Unit FX-3000, Flexcell). The vacuum varied in two-second cycles at a level sufficient to promote cyclic 10 to 25% elongation of the cardiomyocytes at the point of maximal distension of the culture surface. Within each culture, the stretch was started stepwise in the experimetal groups and finished simultaneously to avoid the effect of “ageing” of the cells in culture. In the microarray analysis, the experiments were carried out in two separate sets, each having their own control; I) 1, 4 and 12 hours, and II) 24 and 48 hours of stretch. After the experiments, the cells were washed twice with PBS and quickly frozen at −70 °C or lysed in protein lysis buffer.

### Protein extraction

For the total protein extracts, the cells were lysed in ice-cold lysis buffer (20 mM Tris-HCl, 150 mM NaCl, 1 mM ethylene diaminetetraacetate (EDTA), 1 mM ethylene glycol tetraacetic acid (EGTA), 1% (vol/vol) Triton-X100, 2.5 mM sodium pyrophosphate, 1 mM β-glycerophosphate, 1 mM Na_3_VO_4_) supplemented with 20 μg/ml leupeptin, 2 μg/ml pepstatin, 20 μg/ml aprotinin, 1 mM phenylmethylsulfonyl fluoride (PMSF), 50 mM NaF, 1 mM dithiothreitol (DTT), 6 μg/ml 1-chloro-3-tosylamido-7-phenyl-2-butanone (TPCK), and 6 μg/ml L-1-tosylamido-2-phenylethyl chloromethyl ketone (TLCK)). The lysate was cleared by 10 min centrifugation at +4 °C and the supernatant was transferred into a new tube as the total protein extract. The protein concentration of each sample was determined with a colorimetric assay (Bio-Rad Laboratories).

### Western Blot Analysis

Samples containing 18–25 µg of total protein (n = 5–6 biological replicates at each time point) were boiled in Laemmli buffer, resolved by sodium dodecyl sulphate polyacrylamide gel electrophoresis (SDS-PAGE), and transferred to Optitran BA-S 85 nitrocellulose membranes (Schleicher & Schuell). The membranes were blocked in 5% nonfat milk and then incubated with primary antibody p38 MAPK [#9212], p44/42 (ERK1/2) [#9102], Thr202/Tyr204 phosphorylated ERK (p-ERK1/2) [#9106] and SAPK/JNK (JNK1/2) [#9252] from Cell Signalling Technology (San Diego, CA, USA); Thr180/Tyr182 phosphorylated p38 MAPK (p-p38 MAPK) [AB3828] from Millipore, Thr183/Tyr185 phosphorylated JNK (p-JNK1/2) [sc-6254] from Santa Cruz Biotechnology (Santa Cruz, CA, USA) in 0, 5–1% milk in Tris-buffered saline −0.05% Tween 20 overnight at 4 °C. The antibody concentration in the dilution varied from 1:1000 to 1:2000, depending on the signal strength. On the following day, antibody binding was detected with horseradish peroxidase -linked anti-rabbit or anti-mouse IgG (Cell Signalling Technology) at a 1:2000 dilution and revealed using ECL Plus™ Western Blotting Detection Reagents as described by the manufacturer (GE Healthcare Bio-Sciences Corp. Piscataway, NJ, USA). The chemiluminescence was detected using a Fuji LAS-3000 luminescent image analyzer (Fujifilm, Tokyo, Japan) and bands were quantified with Quantity One software (Bio-Rad).

### Analysis of RNA

Total RNA from cultured myocytes was isolated with TRIzol Reagent according to the manufacturer’s protocol (Invitrogen) by using the Phase Lock Gel system (Eppendorf). In the Northern blot analyzes, 5 μg of RNA were separated by agarose-formaldehyde gel electrophoresis and transferred to a MAGNA nylon membrane (Osmonics Inc.). PCR-amplified probes corresponding to rat genes for BNP and ribosomal 18 S RNA and full-length ANP were random prime-labeled with [α^32^P]dCTP, and the northern blot membranes were hybridized and washed as described previously^74^ and exposed to Phosphor screens (GE Healthcare Life Sciences). The signal was detected with Molecular Imager FX Pro Plus (Bio-Rad) equipment and measured using Quantity One software. The signals of mRNA were normalized to 18 S in each sample to correct for potential differences in loading and/or transfer.

Adrenomedullin (AM), angiotensin Receptor 1 (AT1-R), bone morphogenetic protein-2 (BMP-2), *c-fos*, corin, ET-1, endothelin Receptor Type A (EDNRA), interleukin 6 (IL6), L-type calcium channel α1c-subunit, osteopontin, sarco/endoplasmic reticulum Ca^2+^-ATPase 2a (Serca2a), transforming growth factor β_1_ (TGFβ_1_), TGFβ_2_, xanthine dehydrogenase and 18 S mRNA levels were measured by RT-PCR using TaqMan chemistry on an ABI 7700 Sequence Detection System (Applied Biosystems) as previously described^75^. The sequences of the forward (F) and reverse (R) primers as well as the fluorogenic probes (P) for RNA detection are shown in Supplementary Tables S3. The results were normalized to 18S RNA quantified from the same samples.

### Statistical analysis

Statistical analysis for RNA and protein measurements was performed by One-way Analysis of variance (ANOVA) followed by LSD (least significant difference) post hoc for multiple comparisons. A value of P < 0.05 was considered statistically significant. The results are expressed as mean±SEM.

### DNA microarray analysis

The quality and integrity of the isolated RNA was monitored by gel electrophoresis. Total RNA (n = 5 biological replicates in each group) was used as a template for synthesizing cDNA and for making biotinylated cRNA according to the manufacturer’s instructions (Affymetrix, Santa Clara, CA) as previously described^76^. Briefly, cDNA was reverse-transcripted from 2 μg of total RNA with a T7-(dT)24-primer by means of the One-cycle cDNA synthesis kit (Affymetrix). The DNA was purified using the GeneChip Sample Cleanup Module (Qiagen). The cRNA was prepared and biotin-labeled with *in vitro* transcription (Affymetrix) and fragmented before hybridization. The biotinylated cRNA was hybridized to the GeneChip Rat Expression Set 230_2.0 Arrays, which represents approximately 30,000 rat transcripts. After hybridization, GeneChips were washed and stained with streptavidin-phycoerythrin (Molecular Probes). Staining signal was amplified by biotinylated anti-streptavidin (Vector Laboratories) and the second staining with streptavidin-phycoerythrin was conducted using the Affymetrix Fluidics station according to the standard protocol. A GeneChip Scanner 3000 with GeneChip Operating Software (GCOS) v. 1.2 (Affymetrix) was used in scanning. Affymetrix CEL files were imported into GeneSpring^TM^ GX 12.6 software (Agilent Technologies) and Robust Multichip Average (RMA) normalization was performed. Genes were defined as differentially expressed if the fold change was at least 2.0-fold and statistically significant (*P* < 0.05, One-way ANOVA and Benjamini and Hochberg false discovery rate).

### IPA pathway analysis of differentially expressed genes

Differentially expressed genes (absolute fold-change >1.5; P < 0.05) and their corresponding expression values were uploaded into the Ingenuity Pathway Analysis (IPA) software (Qiagen, Redwood City, CA, USA), and a core analysis was performed separately for each timepoint. The following parameters were used: core analysis, reference set user-defined (i.e., only the set of differentially expressed genes by GeneSpring-software mapped to the IPA database), direct and indirect relationships included, endogenous chemicals included, confidence = experimentally observed.

Next, IPA-software was used to analyze significant associations of biological functions, diseases and well-documented canonical pathways in the Ingenuity Pathway Knowledge database and the differentially expressed genes (Supplementary Dataset 1). The IPA-analysis calculates whether there is an over-representation of significantly up- or down-regulated genes and a particular functional annotation/canonical pathway compared to what is expected by chance alone measured by using Fischer’s exact test. Benjamini–Hochberg procedure was used for multiple testing corrections. An activation z-score was generated for each functional category. This provides a prediction of activation (a positive z-score) or inhibition (negative z-score) of each function annotation. Z-scores ≥2 or ≤−2 indicate statistically significant activation or inhibition, respectively. The entries that had a -Log(P-value) and an absolute z-score value 2 were considered significant. In addition, an IPA-analysis was performed to identify upstream transcriptional regulators that were involved in the stretch activated response at each time point and the activation Z-score was used to predict whether they were likely to be activated or inhibited in order to obtain the observed changes in gene expression in the datasets.

### MicroRNA analysis

MicroRNA array analysis was done by using miRCURY LNA^TM^ Array microRNA profiling services (Exiqon, Vedbaek, Denmark). The sample quality of total RNAs from three samples of both the controls and the stretched samples at each time point was assessed using Bioanalyser 2100 (Agilent), and those RNA integrity number (RIN) values that were greater than 7.6. Samples (n = 3 biological replicates in all timepoints in all groups except n = 2 at 1-hour time point) were labeled with miRCURY LNA^TM^ Hy3^TM^/Hy5^TM^ Power labeling kit and hybridized on the miRCURY LNA^TM^ Array (v.10.0) probes based on miRBase 11.0 annotation. Quantified signals were normalized using the global Locally WEighted Scatterplot Smoothing (LOWESS) regression algorithm. MicroRNAs were defined as differentially expressed by using two-tailed t-test (P < 0.05) for comparing experimental groups of I) 1, 4 and 12 hours, and II) 24 and 48 hours of stretch against their own control.

### Integration of miRNA and mRNA expression data

To identify potential miRNA-mRNA interactions, we correlated differentially regulated miRNAs at each timepoint (Supplementary Datafile 5) with observed gene expression differences at the same or at a later timepoint (Supplementary Datafile 1), and then these were compared with experimentally demonstrated targets of the miRNA using the microRNA Target Filter of IPA software. This analysis provides experimentally validated interactions from TarBase and miRecords, as well as predicted microRNA-mRNA interactions from TargetScan and a large number of microRNA-related findings from the peer-reviewed literature.

### Data availability

Gene expression and miRNA profiling data are publicly available on Gene Expression Omnibus database GEO, http://www.ncbi.nlm.nih.gov/geo/) under the GEO IDs: GSE107551 (gene expression data) and GSE107380 (miRNA expression data).

## Electronic supplementary material

Supplementary File 1

Supplementary File 2

Dataset 1

Dataset 2

Dataset 3

Dataset 5

Dataset 4
