# Supplementary material for: Mechanical stretch induced transcriptomic profiles in cardiac myocytes
Source: Sci Rep. 2018 Mar 16;8:4733. doi: 10.1038/s41598-018-23042-w (PMC5856749; doi:10.1038/s41598-018-23042-w)
Supplement: Supplementary file 1 [file 41598_2018_23042_MOESM1_ESM.pdf]

Supplementary File 1

Mechanical stretch induced transcriptomic profiles in cardiac myocytes

Jaana Rysä[1,2,*], Heikki Tokola[2,3], Heikki Ruskoaho[2,4]

[1]School of Pharmacy, University of Eastern Finland, Kuopio, Finland

[2]Research Unit of Biomedicine, Pharmacology and Toxicology, University of Oulu, Oulu, Finland

[3]Department of Pathology, Cancer Research and Translational Medicine Research Unit, University of Oulu and Oulu University Hospital, Oulu, Finland

[4]Drug Research Program, Division of Pharmacology and Pharmacotherapy, University of Helsinki, Finland

*Corresponding author email: jaana.rysa@uef.fi

Supplementary Figure S1

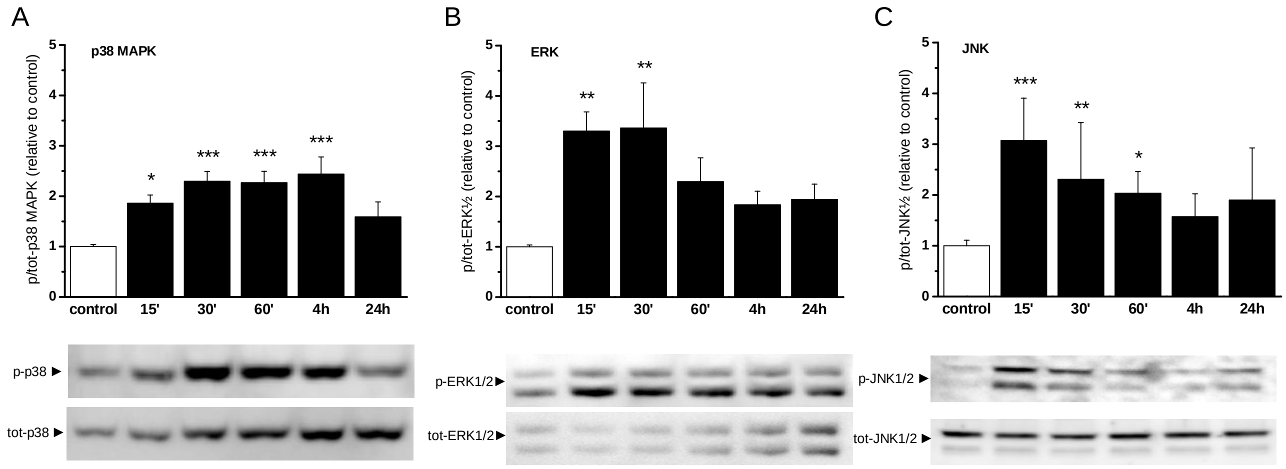

Supplementary Figure S1. Mechanical Stretch Activates Phosphorylation of Mitogen Activated Kinases (MAPKs). (A) p38 MAPK (B), ERK1/2 and (C) JNK1/2. Myocytes were subjected to cyclic stretch for up to 24 hours. After extraction, the total cellular protein was boiled in Laemmli buffer, resolved by SDS-PAGE, and blotted on nitrocellulose membranes. The blots were incubated with p-p38 MAPK-, p-ERK1/2-, p-JNK1/2-, total p38 MAPK-, total ERK1/2- and total-JNK1/2-specific antibodies and detected by enhanced chemiluminescence. The results in bar graphs are expressed as the ratio of the phosphorylated protein kinase to total protein kinase. Open bars indicate control and solid bars indicate stretched cells. Results are the mean ± SEM, n=5-6 (biological replicates). *P<0.05; **P<0.01 and ***P<0.001 vs. non-stretched control (One-way ANOVA followed by LSD post hoc test).

Supplementary Figure S2

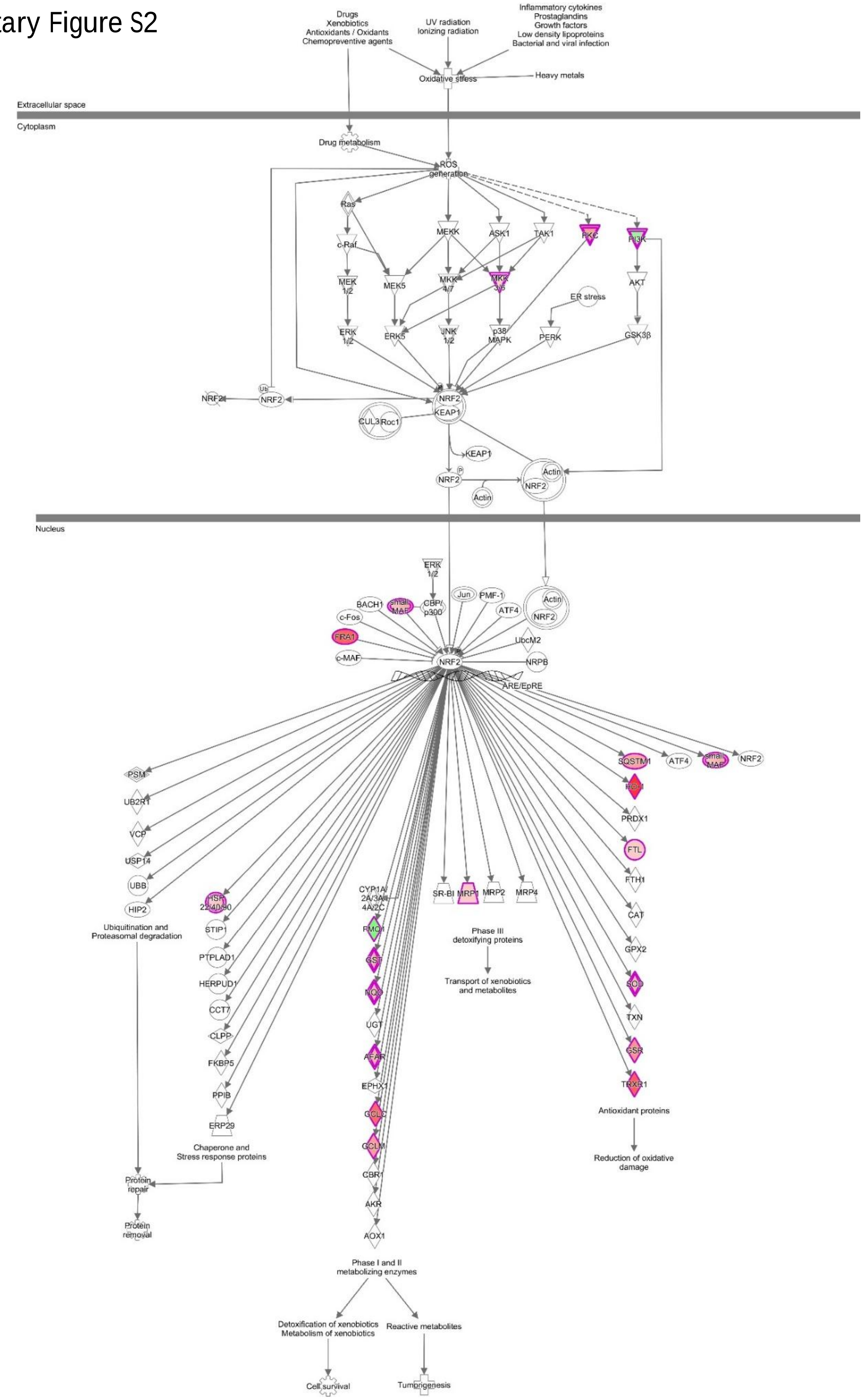

Supplementary Figure S2. Nrf2-mediated oxidative stress response –signalling. Significant canonical pathway associated with differentially expressed genes in response to 12 hours of mechanical stretch in neonatal rat cardiac myocytes. Up- and down-regulated genes in red and green, respectively. The intensity of the color indicates the degree of up-regulation or down regulation. Genes are represented as various shapes that represent the functional class of the gene product.

Supplementary Figure S3

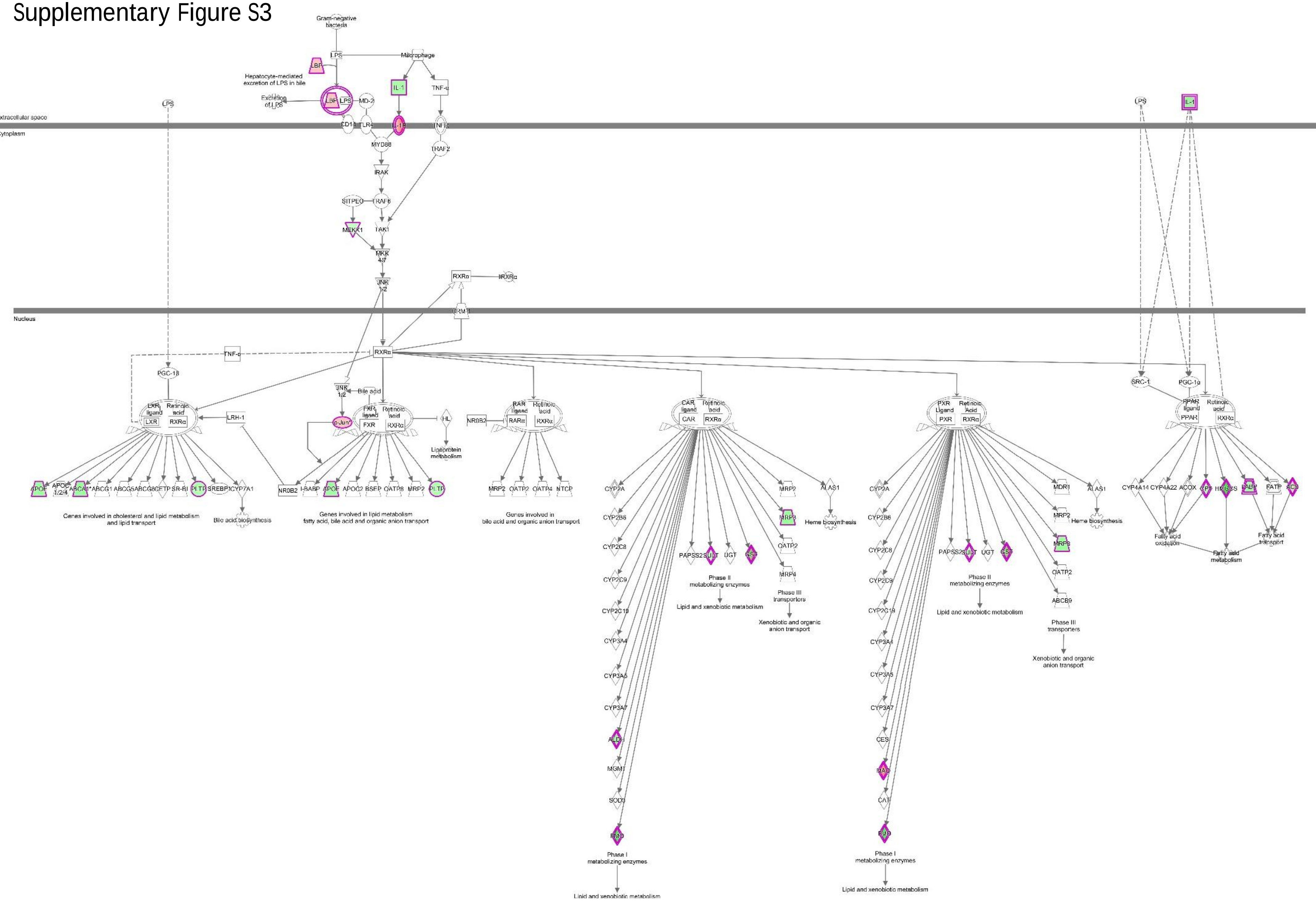

Supplementary Figure S3. LPS/IL-1 mediated inhibition of RXR function –signalling. Significant canonical pathway associated with differentially expressed genes in response to 48 hours of mechanical stretch in neonatal rat cardiac myocytes. Up- and down-regulated genes in red and green, respectively. The intensity of the color indicates the degree of up-regulation or down-regulation. Genes are represented as various shapes that represent the functional class of the gene product.

Table S1. Comparison of microarray results (1-12 h) with Real-time quantitative RT-PCR.

GenBank accession number	Description	Fold Change[a] Microarray 1 h	4 h	12 h	qRT-PCR 1 h	4 h	12 h
NM_012715	Adrenomedullin	1.3**	1.8**	2.4**	1.1	2.1***	2.0**
BI290306	Angiotensin II type 1 receptor	0.7	0.4**	0.2*	0.7	0.6**	0.2*
NM_017178	Bone morphogenetic protein-2	1.7	2.0*	1.8	1.5**	3.1**	1.3*
BF415939	c-fos	2.4***	1.5	1.3	2.3**	2.0**	1.4*
NM_012548	Endothelin-1	0.9	1.0	0.6***	0.8	1.3	0.5**
AB001382	Osteopontin	1.1	1.4	1.7***	1.2	2.1***	2.0**
J04024	Sarco/endoplasmic reticulum Ca2+-ATPase 2a	0.6	0.7	0.6	0.7	0.9	0.7
NM_021578	Transforming growth factor β1	1.3	1.6*	1.5*	1.5***	2.2**	1.5**
AF135598	Transforming growth factor β2	1.2	1.9*	1.3	1.5*	2.8**	1.4*
NM_017154	Xanthine reductase	0.8**	0.6**	0.5***	0.8	0.8**	0.5**

[a]Fold change represents statistically significant increase (*P<0.05, **P<0.01, ***P<0.001) in gene expression in stretched cells after 1 hour, 4 hours or 12 hours of mechanical stretch in stretched cells compared with non-stretched control cells. Measurements of mRNA with real time quantitative RT-PCR (qRTPCR). Serca2a; Sarco/endoplasmic reticulum Ca^{2+}-ATPase 2a.

Table S2. Comparison of microarray results (24-48 h) with Real-time quantitative RT-PCR/Northern blot analysis

GenBank accession number	Description	Fold Change* Microarray 24 h	Microarray 48 h	qRT-PCR 24 h	qRT-PCR 48 h
NM_030985	Angiotensin II type 1 receptor	0.4	0.2**	0.4**	0.2**
NM_012612	Atrial natriuretic peptide	2.6*	2.6*	5.6**[n]	5.2***[n]
NM_017178	Bone morfogenetic protein-2	1.5	2.6*	2.4***	3.2***
NM_031545	B-type natriuretic peptide	1.5*	1.7*	2.3*[n]	3.6***[n]
AI577630	Corin	0.4	0.3*	0.4*	0.4**
M64711	Endothelin-1	1.2	1.2	1.8*	2.0***
NM_012550	Endothelin Receptor Type A	0.6	0.4*	0.6	0.3**
NM_012589	Interleukin-6	3.4*	2.5*	4.5*	3.6*
M59786	L-type calcium channel α1c-subunit	0.7	0.6*	0.7	0.4**
NM_021578	Transforming Growth Factor β1	1.2	1.2	1.4	1.3*
NM_031131	Transforming Growth Factor β2	2.4*	3.8*	2.2**	4.7***

[*a]Fold change represents statistically significant increase (*P<0.05, **P<0.01, ***P<0.001) in gene expression at 24 or 48 hours of mechanical stretch in stretched cells compared with non-stretched control cells. Measurements of mRNA with real time quantitative RT-PCR (qRT-PCR) or [n]Northern blot.

Supplementary Table S3. Oligonucleotide sequences for real time quantitative RT-PCR

Gene	Oligonucleotide sequence
Adrenomedullin	(F) CATTGAACAGTCGGGCGAGT
	(R) CAGGGTGCGAAGCTCTCTG
	(P) CCCATTGGCGCCTGCGGA
Apelin	(F) CAAGGATCCCTTTGGCCC
	(R) AGGAGAAGCTGGGTCTCCAAG
	(P) TCTTCCTGGCCACTCCTTGGACTGC
Angiotensin Receptor 1	(F) GTGGCCAAAGTCACCTGCA
	(R) GTGGATGACAGCTGGCAAACT
	(P) CATCTGGCTGATGGCTGGCTTGG
Bone morphogenetic protein-2	(F) ACACCGTGCTCAGCTTCCAT
	(R) GTCGGGAAGTTTTCCCACTCA
	(P) ACGAAGAAGCCATCGAGGAACTTTCAGAA
c-fos	(F) GGCTGAACCCTTTGATGACTTC
	(R) GGGCAGTCTCCGAGCCA
	(P) TGTTTCCGGCATCATCTAGGC
Corin	(F) CCCAGTGGACATATCTGTGGC
	(R) TTCAAAGCAATGGGCAACTGT
	(P) TGTCCTCATTGCCAAGAAGTGGGTCC
Endothelin-1	(F) ATGGACAAGGAGTGTGTCTACTTCTG
	(R) GGGACGACGCGCTCG
	(P) CACCTGGACATCATCTGGGTCAACACTC
Endothelin receptor type A	(F) GGAATGGGAGCTTGCGG
	(R) TTTGCCACCTCTCGACGC
	(P) TTGCCCTCAGCGAACACCTCAAGC
Inteleukin-6	(F) ATATGTTCTCAGGGAGATCTTGGAA
	(R) TGCATCATCGCTGTTCATACAA
	(P) CAGAATTGCCATTGCACAACTCTTTTCTCA
Osteopontin	(F) AATCGCCCCCACAGTCG
	(R) CCTCAGTCCGTAAGCCAAGC
	(P) TGTCCCTGACGGCCGAGGTGA
Sarco/endoplasmic reticulum	(F) CAGCCATGGAGAACGCTCA
Ca^{2+}-ATPase 2a	(R) CGTTGACGCCGAAGTGG
	(P) ACAAAGACCGTGGAGGAGGTGCTGG
Transforming growth factor β1	(F) CATCGACATGGAGCTGGTGA
	(R) TTGGACAGGATCTGGCCAC
	(P) ACGGAAGCGCATCGAAGCCATC
Transforming growth factor β2	(F) ACCTTTTTGCTCCTGCATCTG
	(R) GTCGAGGGTGCTGCAGGTA
	(P) TCCCGGTGGCGCTCAGTCTGT
Xanthine dehydrogenase	(F) CCTCCAGGGATTCCGGAC
	(R) TGGGTTGTTTCCACTTCCTCC
	(P) TTTGCCAAGGATGGTGGGTGCTG
18S	(F) TGGTTGCAAAGCTGAAACTTAAAG
	(R) AGTCAAATTAAGCCGCAGGC
	(P) CCTGGTGGTGCCCTTCCGTCA

F, forward primer; R, reverse primer; P, fluorogenic probe
