# Supplementary material for: Mechanical stretch induced transcriptomic profiles in cardiac myocytes
Source: Sci Rep. 2018 Mar 16;8:4733. doi: 10.1038/s41598-018-23042-w (PMC5856749; doi:10.1038/s41598-018-23042-w)
Supplement: Supplementary file 2 [file 41598_2018_23042_MOESM2_ESM.pdf]

# Supplementary File 2

Mechanical stretch induced transcriptomic profiles in cardiac myocytes

Jaana Rysä[1,2,*], Heikki Tokola[2,3], Heikki Ruskoaho[2,4]

[1]School of Pharmacy, University of Eastern Finland, Kuopio, Finland

[2]Research Unit of Biomedicine, Pharmacology and Toxicology, University of Oulu, Oulu, Finland

[3]Department of Pathology, Cancer Research and Translational Medicine Research Unit, University of Oulu and Oulu University Hospital, Oulu, Finland

[4]Drug Research Program, Division of Pharmacology and Pharmacotherapy, University of Helsinki, Finland

*Corresponding author email: jaana.rysa@uef.fi

# 1 h of stretching, network 1

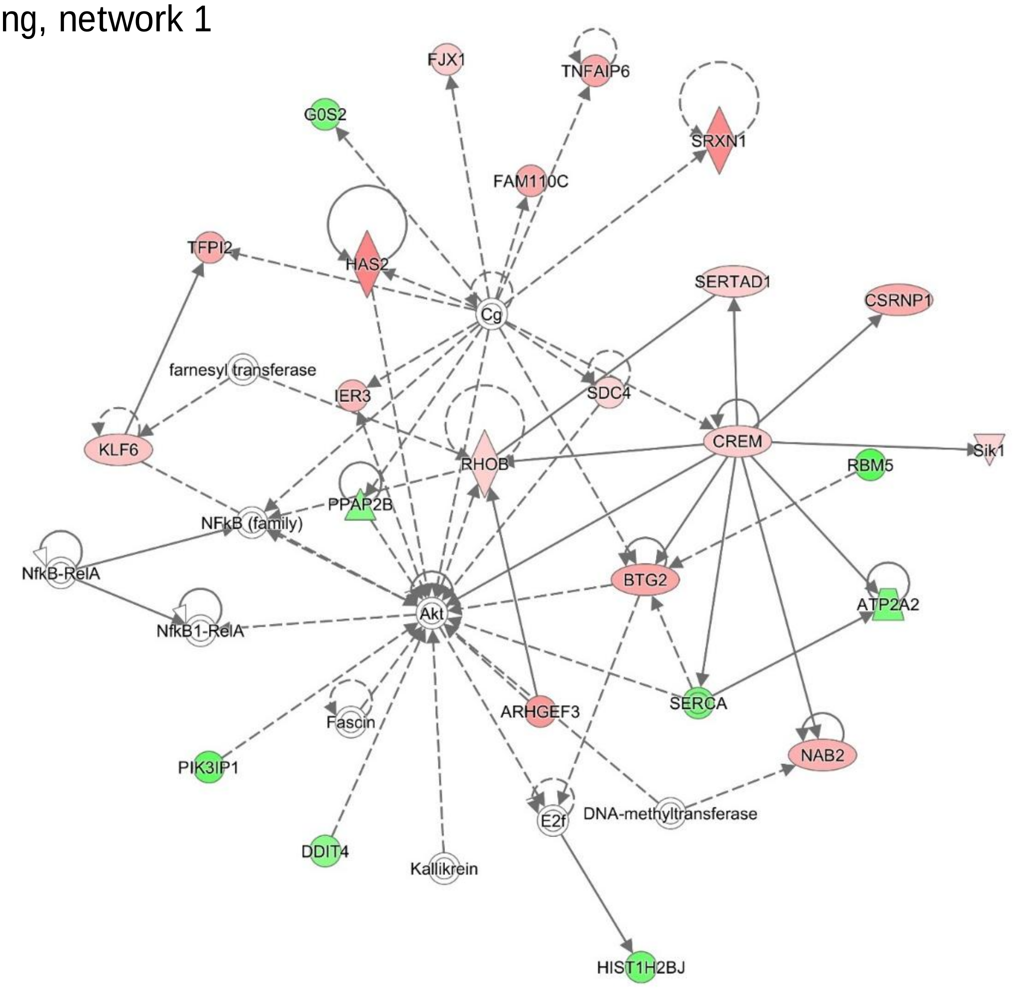


| Symbol | Gene Name | Fold change | Location | Family |
|---|---|---|---|---|
| Akt | | | Cytoplasm | group |
| ARHGEF3 | Rho guanine nucleotide exchange factor 3 | 2.8 | Cytoplasm | other |
| ATP2A2 | ATPase sarcoplasmic/endoplasmic reticulum Ca2+ transporting 2 | 0.6 | Cytoplasm | transporter |
| BTG2 | BTG anti-proliferation factor 2 | 2.4 | Nucleus | transcription regulator |
| Cg | | | Other | complex |
| CREM | cAMP responsive element modulator | 1.7 | Nucleus | transcription regulator |
| CSRNP1 | cysteine and serine rich nuclear protein 1 | 2.3 | Nucleus | transcription regulator |
| DDIT4 | DNA damage inducible transcript 4 | 0.7 | Cytoplasm | other |
| DNA-methyltransferase | | | Other | group |
| E2f | | | Nucleus | group |
| FAM110C | family with sequence similarity 110 member C | 2.4 | Cytoplasm | other |
| farnesyl transferase | | | Other | complex |
| Fascin | | | Cytoplasm | group |
| FJX1 | four jointed box 1 | 1.6 | Extracellular Space | other |
| G0S2 | G0/G1 switch 2 | 0.6 | Cytoplasm | other |
| HAS2 | hyaluronan synthase 2 | 3.4 | Plasma Membrane | enzyme |
| HIST1H2BJ | histone cluster 1 H2B family member j | 0.6 | Nucleus | other |
| IER3 | immediate early response 3 | 2.0 | Cytoplasm | other |
| Kallikrein | | | Extracellular Space | group |
| KLF6 | Kruppel like factor 6 | 1.8 | Nucleus | transcription regulator |
| NAB2 | NGFI-A binding protein 2 | 2.2 | Nucleus | transcription regulator |
| NFkB (family) | | | Nucleus | group |
| NfkB-RelA | | | Nucleus | complex |
| NfkB1-RelA | | | Nucleus | complex |
| PIK3IP1 | phosphoinositide-3-kinase interacting protein 1 | 0.6 | Cytoplasm | other |
| PPAP2B | phospholipid phosphatase 3 | 0.6 | Plasma Membrane | phosphatase |
| RBM5 | RNA binding motif protein 5 | 0.5 | Nucleus | other |
| RHOB | ras homolog family member B | 1.6 | Cytoplasm | enzyme |
| SDC4 | syndecan 4 | 1.5 | Plasma Membrane | other |
| SERCA | | | Cytoplasm | group |
| SERTAD1 | SERTA domain containing 1 | 1.6 | Nucleus | transcription regulator |
| Sik1 | salt inducible kinase 1 | 1.5 | Other | kinase |
| SRXN1 | sulfiredoxin 1 | 3.2 | Cytoplasm | enzyme |
| TFPI2 | tissue factor pathway inhibitor 2 | 2.3 | Extracellular Space | other |
| TNFAIP6 | TNF alpha induced protein 6 | 2.4 | Extracellular Space | other |

# 1 h of stretching, network 2

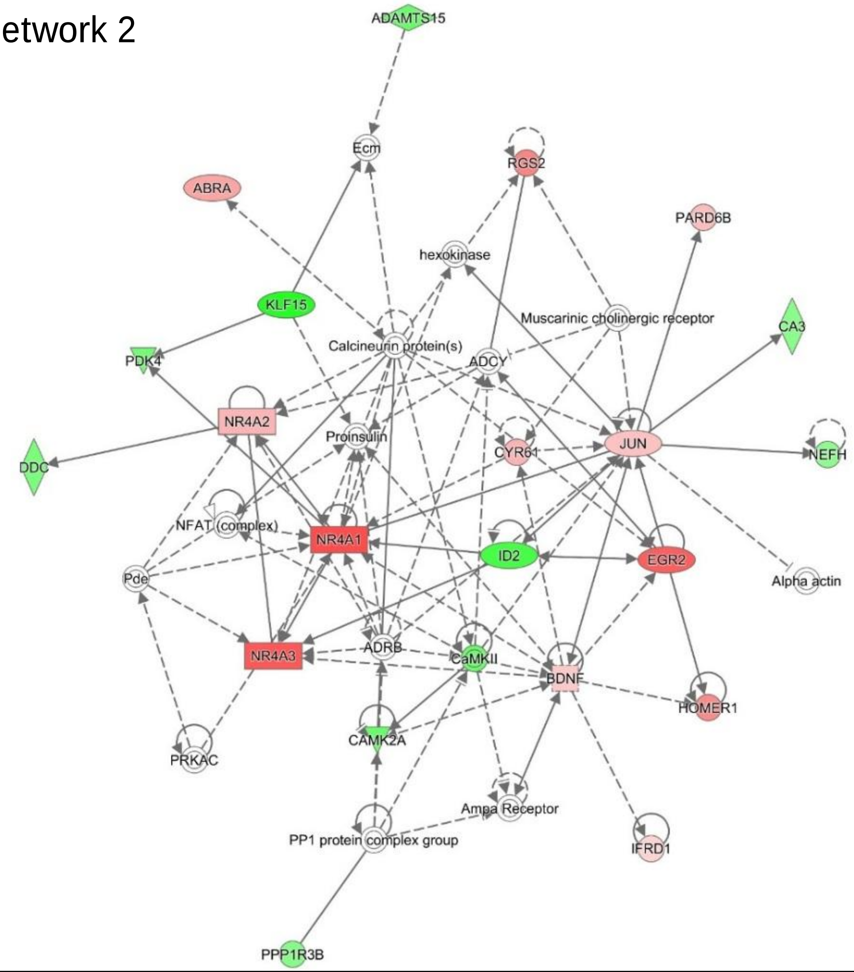


| Symbol | Gene Name | Fold change | Location | Family |
|---|---|---|---|---|
| ABRA | actin binding Rho activating protein | 2.5 | Cytoplasm | transcription regulator |
| ADAMTS15 | ADAM metallopeptidase with thrombospondin type 1 motif 15 | 0.6 | Extracellular Space | peptidase |
| ADCY | | | Cytoplasm | group |
| ADRB | | | Plasma Membrane | group |
| Alpha actin | | | Cytoplasm | group |
| Ampa Receptor | | | Other | complex |
| BDNF | brain derived neurotrophic factor | 1.7 | Extracellular Space | growth factor |
| CA3 | carbonic anhydrase 3 | 0.7 | Cytoplasm | enzyme |
| Calcineurin protein(s) | | | Cytoplasm | complex |
| CAMK2A | calcium/calmodulin dependent protein kinase II alpha | 0.6 | Cytoplasm | kinase |
| CaMKII | | | Cytoplasm | complex |
| CYR61 | cysteine rich angiogenic inducer 61 | 2.1 | Extracellular Space | other |
| DDC | dopa decarboxylase | 0.6 | Cytoplasm | enzyme |
| Ecm | | | Other | group |
| EGR2 | early growth response 2 | 5.0 | Nucleus | transcription regulator |
| hexokinase | | | Other | group |
| HOMER1 | homer scaffolding protein 1 | 3.2 | Plasma Membrane | other |
| ID2 | inhibitor of DNA binding 2. HLH protein | 0.5 | Nucleus | transcription regulator |
| IFRD1 | interferon related developmental regulator 1 | 1.5 | Nucleus | other |
| JUN | Jun proto-oncogene. AP-1 transcription factor subunit | 1.8 | Nucleus | transcription regulator |
| KLF15 | Kruppel like factor 15 | 0.5 | Nucleus | transcription regulator |
| Muscarinic cholinergic receptor | | | Plasma Membrane | group |
| NEFH | neurofilament heavy polypeptide | 0.7 | Cytoplasm | other |
| NFAT (complex) | | | Cytoplasm | complex |
| NR4A1 | nuclear receptor subfamily 4 group A member 1 | 6.0 | Nucleus | ligand-dependent nuclear receptor |
| NR4A2 | nuclear receptor subfamily 4 group A member 2 | 2.1 | Nucleus | ligand-dependent nuclear receptor |
| NR4A3 | nuclear receptor subfamily 4 group A member 3 | 5.3 | Nucleus | ligand-dependent nuclear receptor |
| PARD6B | par-6 family cell polarity regulator beta | 1.9 | Plasma Membrane | other |
| Pde | | | Cytoplasm | group |
| PDK4 | pyruvate dehydrogenase kinase 4 | 0.6 | Cytoplasm | kinase |
| PP1 protein complex group | | | Cytoplasm | complex |
| PPP1R3B | protein phosphatase 1 regulatory subunit 3B | 0.6 | Cytoplasm | other |
| PRKAC | PRKAC | | Cytoplasm | group |
| Proinsulin | | | Other | group |
| RGS2 | regulator of G-protein signaling 2 | 3.4 | Nucleus | other |

# 1 h of stretching, network 3

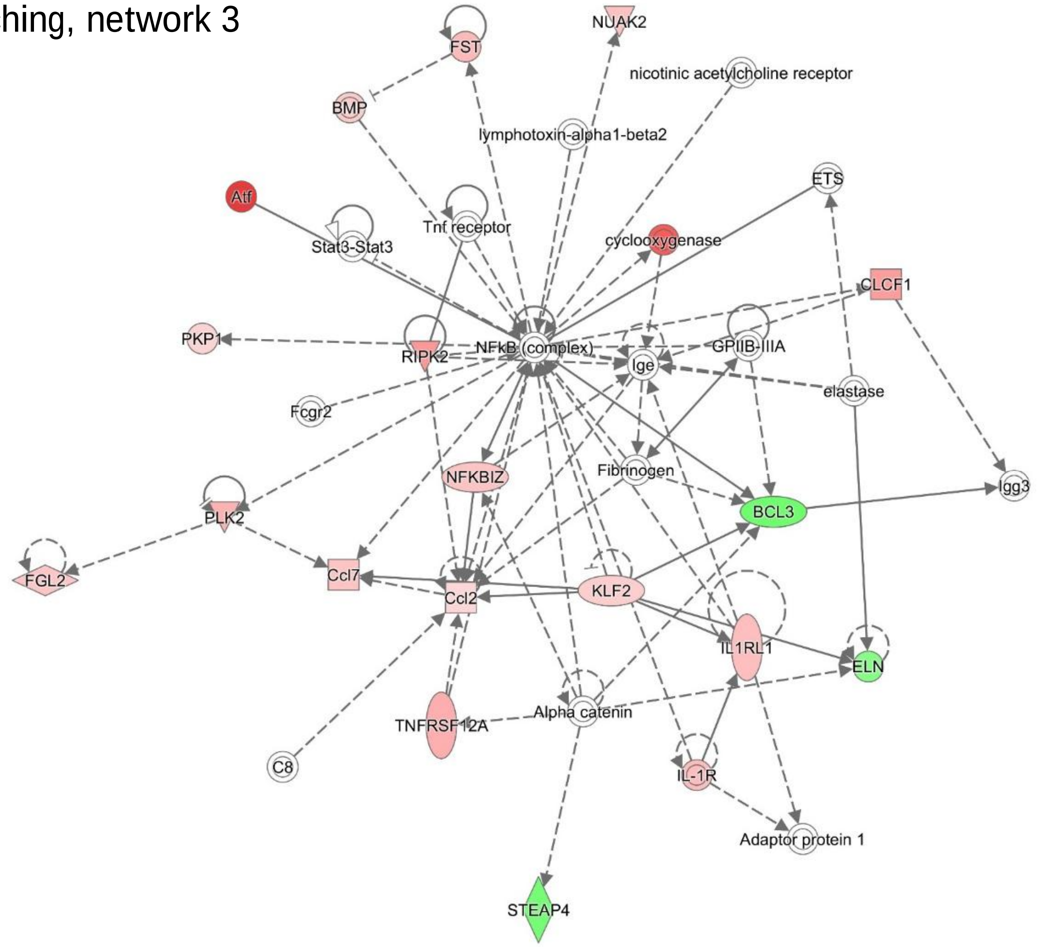


| Symbol | Gene Name | Fold change | Location | Family |
|---|---|---|---|---|
| Adaptor protein 1 | | | Cytoplasm | complex |
| Alpha catenin | | | Cytoplasm | group |
| Atf | | | Nucleus | group |
| BCL3 | B-cell CLL/lymphoma 3 | 0.6 | Nucleus | transcription regulator |
| BMP | | | Extracellular Space | group |
| C8 | | | Cytoplasm | complex |
| Ccl2 | chemokine (C-C motif) ligand 2 | 1.5 | Extracellular Space | cytokine |
| Ccl7 | chemokine (C-C motif) ligand 7 | 1.8 | Extracellular Space | cytokine |
| CLCF1 | cardiotrophin-like cytokine factor 1 | 2.7 | Extracellular Space | cytokine |
| cyclooxygenase | | | Cytoplasm | group |
| elastase | | | Other | group |
| ELN | elastin | 0.6 | Extracellular Space | other |
| ETS | | | Nucleus | group |
| Fcgr2 | | | Plasma Membrane | group |
| FGL2 | fibrinogen like 2 | 1.7 | Extracellular Space | peptidase |
| Fibrinogen | | | Plasma Membrane | complex |
| FST | follistatin | 2.0 | Extracellular Space | other |
| GPIIB-IIIA | | | Plasma Membrane | complex |
| Ige | | | Extracellular Space | complex |
| Igg3 | | | Other | complex |
| IL-1R | | | Plasma Membrane | group |
| IL1RL1 | interleukin 1 receptor like 1 | 1.9 | Plasma Membrane | transmembrane receptor |
| KLF2 | Kruppel like factor 2 | 1.6 | Nucleus | transcription regulator |
| lymphotoxin-alpha1-beta2 | lymphotoxin-alpha1-beta2 | | Extracellular Space | complex |
| NFkB (complex) | | | Nucleus | complex |
| NFKBIZ | NFKB inhibitor zeta | 1.8 | Nucleus | transcription regulator |
| nicotinic acetylcholine receptor | | | Plasma Membrane | complex |
| NUAK2 | NUAK family kinase 2 | 1.8 | Other | kinase |
| PKP1 | plakophilin 1 | 1.5 | Plasma Membrane | other |
| PLK2 | polo like kinase 2 | 2.1 | Nucleus | kinase |
| RIPK2 | receptor interacting serine/threonine kinase 2 | 2.8 | Plasma Membrane | kinase |
| Stat3-Stat3 | | | Nucleus | complex |
| STEAP4 | STEAP4 metalloreductase | 0.6 | Plasma Membrane | enzyme |
| Tnf receptor | | | Plasma Membrane | group |
| TNFRSF12A | TNF receptor superfamily member 12A | 2.3 | Plasma Membrane | transmembrane receptor |

# 1 h of stretching, network 4

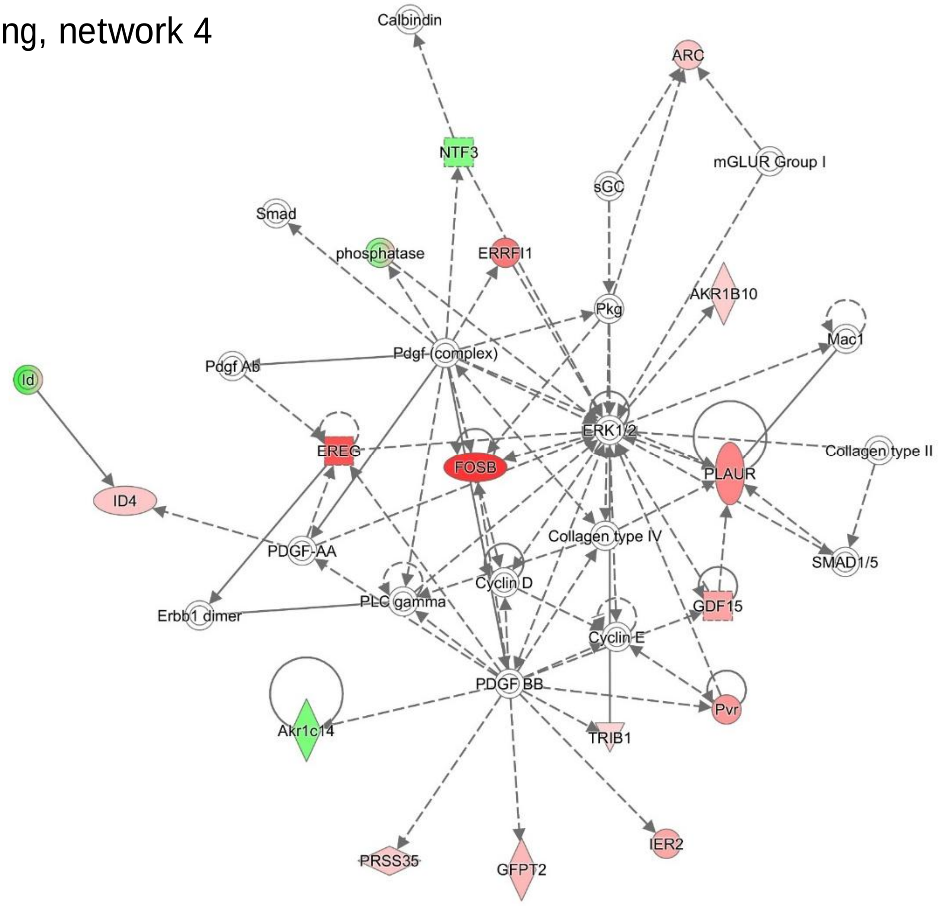


| Symbol | Gene Name | Fold change | Location | Family |
|---|---|---|---|---|
| AKR1B10 | aldo-keto reductase family 1 member B10 | 1.6 | Cytoplasm | enzyme |
| Akr1c14 | aldo-keto reductase family 1. member C14 | 0.6 | Cytoplasm | enzyme |
| ARC | activity regulated cytoskeleton associated protein | 1.8 | Cytoplasm | other |
| Calbindin | | | Other | group |
| Collagen type II | | | Other | complex |
| Collagen type IV | | | Other | complex |
| Cyclin D | | | Nucleus | group |
| Cyclin E | | | Nucleus | group |
| Erbb1 dimer | | | Plasma Membrane | complex |
| EREG | epiregulin | 5.8 | Extracellular Space | growth factor |
| ERK1/2 | | | Cytoplasm | group |
| ERRFI1 | ERBB receptor feedback inhibitor 1 | 4.0 | Cytoplasm | other |
| FOSB | FosB proto-oncogene. AP-1 transcription factor subunit | 8.0 | Nucleus | transcription regulator |
| GDF15 | growth differentiation factor 15 | 2.5 | Extracellular Space | growth factor |
| GFPT2 | glutamine-fructose-6-phosphate transaminase 2 | 2.0 | Cytoplasm | enzyme |
| Id | | | Other | group |
| ID4 | inhibitor of DNA binding 4. HLH protein | 1.7 | Nucleus | transcription regulator |
| IER2 | immediate early response 2 | 2.4 | Cytoplasm | other |
| Mac1 | Mac1 | | Plasma Membrane | complex |
| mGLUR Group I | | | Plasma Membrane | group |
| NTF3 | neurotrophin 3 | 0.6 | Extracellular Space | growth factor |
| Pdgf (complex) | | | Extracellular Space | complex |
| Pdgf Ab | | | Plasma Membrane | complex |
| PDGF BB | | | Extracellular Space | complex |
| PDGF-AA | | | Extracellular Space | complex |
| phosphatase | | | Other | group |
| Pkg | | | Cytoplasm | group |
| PLAUR | plasminogen activator. urokinase receptor | 3.4 | Plasma Membrane | transmembrane receptor |
| PLC gamma | | | Cytoplasm | group |
| PRSS35 | protease. serine 35 | 1.7 | Extracellular Space | peptidase |
| Pvr | poliovirus receptor | 2.8 | Plasma Membrane | other |
| sGC | | | Cytoplasm | complex |
| Smad | | | Nucleus | complex |
| SMAD1/5 | | | Cytoplasm | group |
| TRIB1 | tribbles pseudokinase 1 | 1.5 | Cytoplasm | kinase |

1 h of stretching, network 5

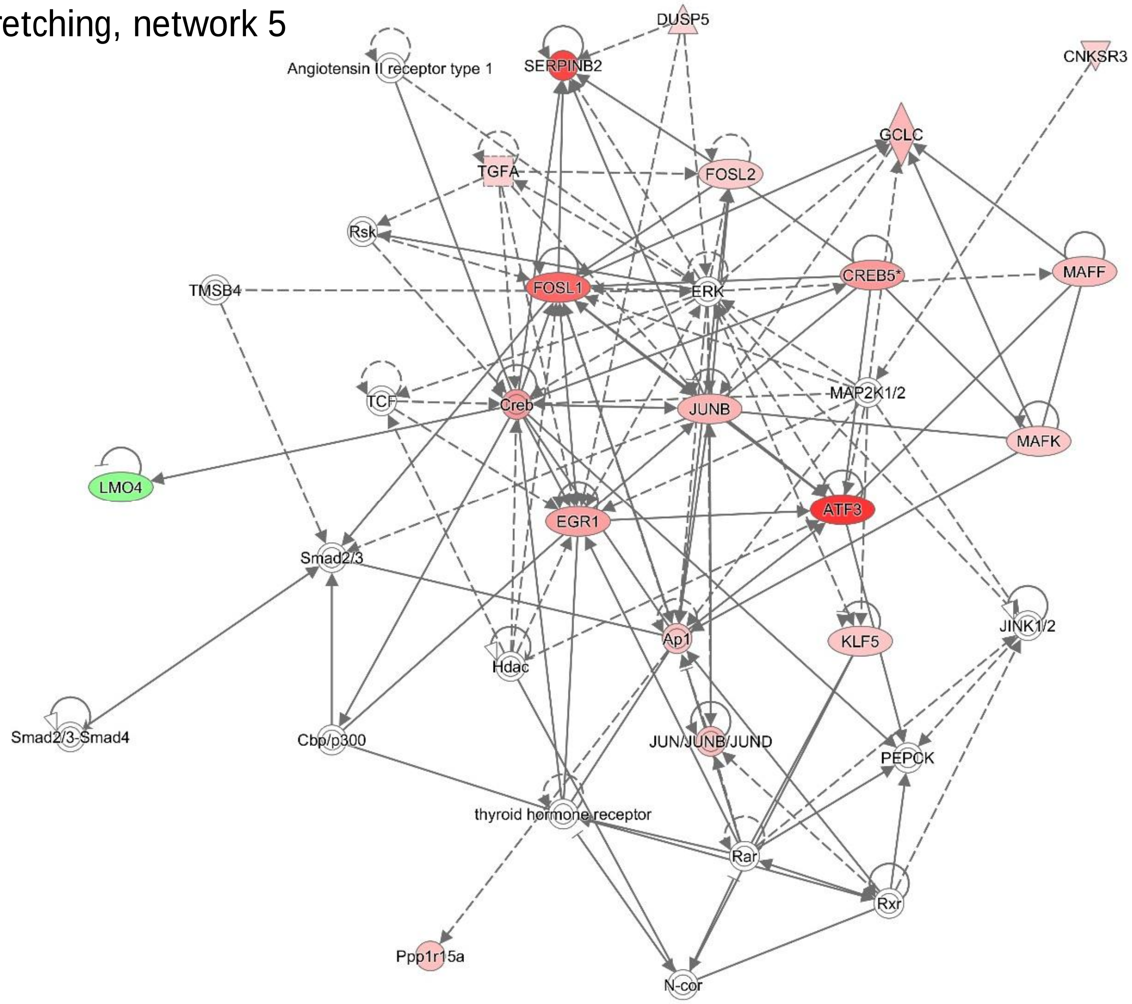


| Symbol | Gene Name | Fold change | Location | Family |
|---|---|---|---|---|
| Angiotensin II receptor type 1 | | | Plasma Membrane | group |
| Ap1 | | | Nucleus | complex |
| ATF3 | activating transcription factor 3 | 8.6 | Nucleus | transcription regulator |
| Cbp/p300 | | | Nucleus | group |
| CNKSR3 | CNKSR family member 3 | 1.6 | Plasma Membrane | kinase |
| Creb | | | Nucleus | group |
| CREB5 | cAMP responsive element binding protein 5 | 2.8 | Nucleus | transcription regulator |
| DUSP5 | dual specificity phosphatase 5 | 1.5 | Nucleus | phosphatase |
| EGR1 | early growth response 1 | 2.6 | Nucleus | transcription regulator |
| ERK | | | Other | group |
| FOSL1 | FOS like 1. AP-1 transcription factor subunit | 4.7 | Nucleus | transcription regulator |
| FOSL2 | FOS like 2. AP-1 transcription factor subunit | 1.7 | Nucleus | transcription regulator |
| GCLC | glutamate-cysteine ligase catalytic subunit | 2.1 | Cytoplasm | enzyme |
| Hdac | | | Nucleus | group |
| JINK1/2 | | | Cytoplasm | group |
| JUN/JUNB/JUND | | | Nucleus | group |
| JUNB | JunB proto-oncogene. AP-1 transcription factor subunit | 2.1 | Nucleus | transcription regulator |
| KLF5 | Kruppel like factor 5 | 1.8 | Nucleus | transcription regulator |
| LMO4 | LIM domain only 4 | 0.7 | Nucleus | transcription regulator |
| MAFF | MAF bZIP transcription factor F | 1.9 | Nucleus | transcription regulator |
| MAFK | MAF bZIP transcription factor K | 1.7 | Nucleus | transcription regulator |
| MAP2K1/2 | | | Cytoplasm | group |
| N-cor | | | Nucleus | group |
| PEPCK | | | Nucleus | group |
| Ppp1r15a | protein phosphatase 1. regulatory subunit 15A | 1.9 | Cytoplasm | other |
| Rar | | | Nucleus | group |
| Rsk | | | Cytoplasm | group |
| Rxr | | | Nucleus | group |
| SERPINB2 | serpin family B member 2 | 6.6 | Extracellular Space | other |
| Smad2/3 | | | Cytoplasm | group |
| Smad2/3-Smad4 | | | Nucleus | complex |
| TCF | | | Other | group |
| TGFA | transforming growth factor alpha | 1.6 | Extracellular Space | growth factor |
| thyroid hormone receptor | | | Nucleus | group |
| TMSB4 | | | Cytoplasm | group |

4 hrs of stretching, network 1

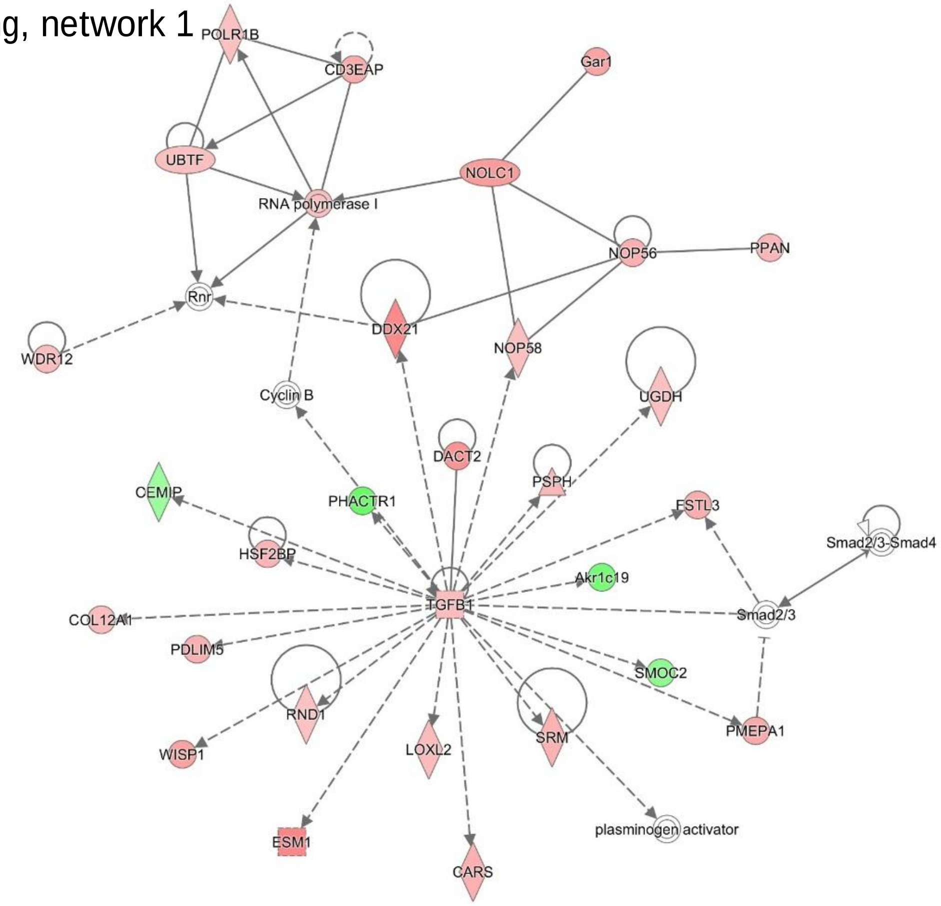


| Symbol | Gene Name | Fold change | Location | Family |
|---|---|---|---|---|
| Akr1c19 | aldo-keto reductase family 1. member C19 | 0.5 | Other | other |
| CARS | cysteinyl-tRNA synthetase | 1.7 | Cytoplasm | enzyme |
| CD3EAP | CD3e molecule associated protein | 1.8 | Nucleus | other |
| CEMIP | cell migration inducing hyaluronan binding protein | 0.6 | Cytoplasm | enzyme |
| COL12A1 | collagen type XII alpha 1 chain | 1.6 | Extracellular Space | other |
| Cyclin B | | | Cytoplasm | group |
| DACT2 | dishevelled binding antagonist of beta catenin 2 | 2.1 | Cytoplasm | other |
| DDX21 | DExD-box helicase 21 | 2.2 | Nucleus | enzyme |
| ESM1 | endothelial cell specific molecule 1 | 2.3 | Extracellular Space | growth factor |
| FSTL3 | follistatin like 3 | 1.8 | Extracellular Space | other |
| Gar1 | GAR1 ribonucleoprotein | 1.9 | Nucleus | other |
| HSF2BP | heat shock transcription factor 2 binding protein | 1.7 | Cytoplasm | other |
| LOXL2 | lysyl oxidase like 2 | 1.6 | Extracellular Space | enzyme |
| NOLC1 | nucleolar and coiled-body phosphoprotein 1 | 2.0 | Nucleus | transcription regulator |
| NOP56 | NOP56 ribonucleoprotein | 1.7 | Nucleus | other |
| NOP58 | NOP58 ribonucleoprotein | 1.6 | Nucleus | enzyme |
| PDLIM5 | PDZ and LIM domain 5 | 1.7 | Cytoplasm | other |
| PHACTR1 | phosphatase and actin regulator 1 | 0.5 | Cytoplasm | other |
| plasminogen activator | | | Extracellular Space | group |
| PMEPA1 | prostate transmembrane protein. androgen induced 1 | 1.8 | Plasma Membrane | other |
| POLR1B | RNA polymerase I subunit B | 1.5 | Nucleus | enzyme |
| PPAN | peter pan homolog (Drosophila) | 1.7 | Nucleus | other |
| PSPH | phosphoserine phosphatase | 1.7 | Cytoplasm | phosphatase |
| RNA polymerase I | | | Nucleus | complex |
| RND1 | Rho family GTPase 1 | 1.5 | Cytoplasm | enzyme |
| Rnr | | | Other | group |
| Smad2/3 | | | Cytoplasm | group |
| Smad2/3-Smad4 | | | Nucleus | complex |
| SMOC2 | SPARC related modular calcium binding 2 | 0.6 | Extracellular Space | other |
| SRM | spermidine synthase | 1.7 | Cytoplasm | enzyme |
| TGFB1 | transforming growth factor beta 1 | 1.6 | Extracellular Space | growth factor |
| UBTF | upstream binding transcription factor. RNA polymerase I | 1.6 | Nucleus | transcription regulator |
| UGDH | UDP-glucose 6-dehydrogenase | 1.5 | Nucleus | enzyme |
| WDR12 | WD repeat domain 12 | 1.6 | Cytoplasm | other |
| WISP1 | WNT1 inducible signaling pathway protein 1 | 1.9 | Extracellular Space | other |

# 4 hrs of stretching, network 2

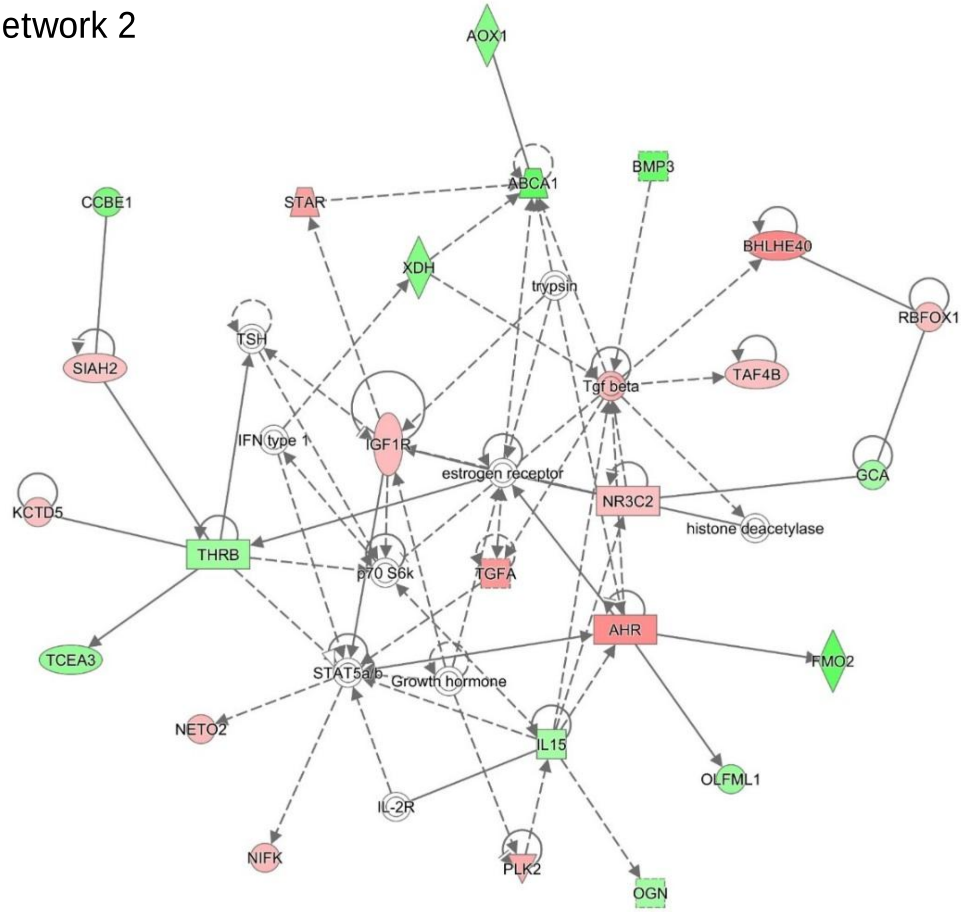


| Symbol | Gene Name | Fold change | Location | Family |
|---|---|---|---|---|
| ABCA1 | ATP binding cassette subfamily A member 1 | 0.5 | Plasma Membrane | transporter |
| AHR | aryl hydrocarbon receptor | 2.2 | Nucleus | ligand-dependent nuclear receptor |
| AOX1 | aldehyde oxidase 1 | 0.6 | Cytoplasm | enzyme |
| BHLHE40 | basic helix-loop-helix family member e40 | 2.3 | Nucleus | transcription regulator |
| BMP3 | bone morphogenetic protein 3 | 0.5 | Extracellular Space | growth factor |
| CCBE1 | collagen and calcium binding EGF domains 1 | 0.5 | Extracellular Space | other |
| estrogen receptor | | 1.0 | Nucleus | group |
| FMO2 | flavin containing monooxygenase 2 | 0.5 | Cytoplasm | enzyme |
| GCA | grancalcin | 0.7 | Cytoplasm | other |
| Growth hormone | | 1.0 | Extracellular Space | group |
| histone deacetylase | | 1.0 | Other | complex |
| IFN type 1 | | 1.0 | Other | group |
| IGF1R | insulin like growth factor 1 receptor | 1.6 | Plasma Membrane | transmembrane receptor |
| IL-2R | | 1.0 | Plasma Membrane | complex |
| IL15 | interleukin 15 | 0.7 | Extracellular Space | cytokine |
| KCTD5 | potassium channel tetramerization domain containing 5 | 1.5 | Cytoplasm | other |
| NETO2 | neuropilin and tolloid like 2 | 1.6 | Plasma Membrane | other |
| NIFK | nucleolar protein interacting with the FHA domain of MKI67 | 1.6 | Nucleus | other |
| NR3C2 | nuclear receptor subfamily 3 group C member 2 | 1.5 | Nucleus | ligand-dependent nuclear receptor |
| OGN | osteoglycin | 0.7 | Extracellular Space | growth factor |
| OLFML1 | olfactomedin like 1 | 0.6 | Extracellular Space | other |
| p70 S6k | | 1.0 | Cytoplasm | group |
| PLK2 | polo like kinase 2 | 1.8 | Nucleus | kinase |
| RBFOX1 | RNA binding protein. fox-1 homolog 1 | 1.6 | Cytoplasm | other |
| SIAH2 | siah E3 ubiquitin protein ligase 2 | 1.5 | Nucleus | transcription regulator |
| STAR | steroidogenic acute regulatory protein | 1.9 | Cytoplasm | transporter |
| STAT5a/b | | 1.0 | Cytoplasm | group |
| TAF4B | TATA-box binding protein associated factor 4b | 1.5 | Nucleus | transcription regulator |
| TCEA3 | transcription elongation factor A3 | 0.6 | Nucleus | transcription regulator |
| Tgf beta | | 1.0 | Extracellular Space | group |
| TGFA | transforming growth factor alpha | 2.0 | Extracellular Space | growth factor |
| THRB | thyroid hormone receptor beta | 0.6 | Nucleus | ligand-dependent nuclear receptor |
| trypsin | | 1.0 | Extracellular Space | group |
| TSH | | 1.0 | Plasma Membrane | complex |
| XDH | xanthine dehydrogenase | 0.6 | Cytoplasm | enzyme |

# 4 hrs of stretching, network 3

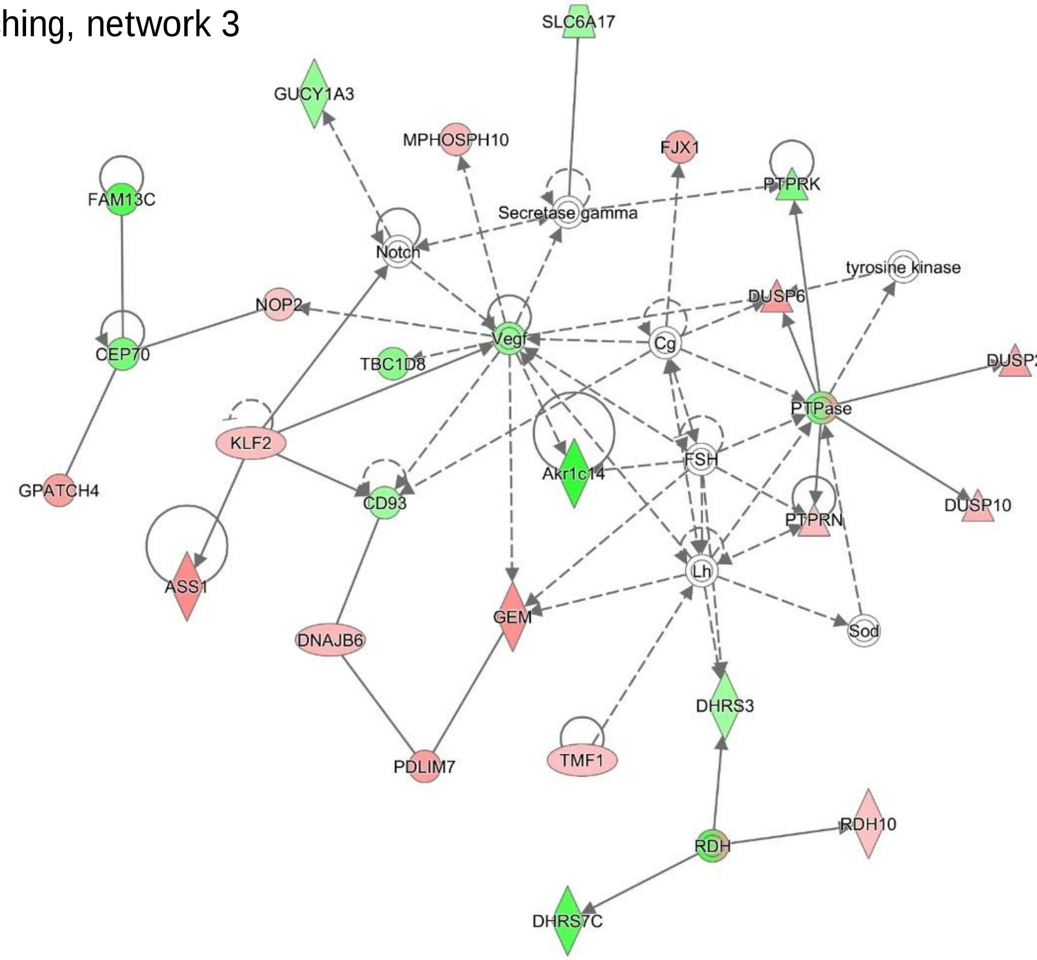


| Symbol | Gene Name | Fold change | Location | Family |
|---|---|---|---|---|
| Akr1c14 | aldo-keto reductase family 1. member C14 | 0.4 | Cytoplasm | enzyme |
| ASS1 | argininosuccinate synthase 1 | 2.2 | Cytoplasm | enzyme |
| CD93 | CD93 molecule | 0.6 | Plasma Membrane | other |
| CEP70 | centrosomal protein 70 | 0.5 | Cytoplasm | other |
| Cg | | 1.0 | Other | complex |
| DHRS3 | dehydrogenase/reductase 3 | 0.6 | Cytoplasm | enzyme |
| DHRS7C | dehydrogenase/reductase 7C | 0.5 | Cytoplasm | enzyme |
| DNAJB6 | DnaJ heat shock protein family (Hsp40) member B6 | 1.6 | Nucleus | transcription regulator |
| DUSP10 | dual specificity phosphatase 10 | 1.7 | Nucleus | phosphatase |
| DUSP2 | dual specificity phosphatase 2 | 1.9 | Nucleus | phosphatase |
| DUSP6 | dual specificity phosphatase 6 | 2.0 | Cytoplasm | phosphatase |
| FAM13C | family with sequence similarity 13 member C | 0.4 | Other | other |
| FJX1 | four jointed box 1 | 1.8 | Extracellular Space | other |
| FSH | | 1.0 | Plasma Membrane | complex |
| GEM | GTP binding protein overexpressed in skeletal muscle | 2.1 | Plasma Membrane | enzyme |
| GPATCH4 | G-patch domain containing 4 | 1.9 | Other | other |
| GUCY1A3 | guanylate cyclase 1 soluble subunit alpha | 0.6 | Cytoplasm | enzyme |
| KLF2 | Kruppel like factor 2 | 1.6 | Nucleus | transcription regulator |
| Lh | | 1.0 | Plasma Membrane | complex |
| MPHOSPH10 | M-phase phosphoprotein 10 | 1.7 | Nucleus | other |
| NOP2 | NOP2 nucleolar protein | 1.5 | Nucleus | other |
| Notch | | 1.0 | Plasma Membrane | group |
| PDLIM7 | PDZ and LIM domain 7 | 1.9 | Cytoplasm | other |
| PTPase | | 1.0 | Cytoplasm | group |
| PTPRK | protein tyrosine phosphatase. receptor type K | 0.6 | Plasma Membrane | phosphatase |
| PTPRN | protein tyrosine phosphatase. receptor type N | 1.6 | Plasma Membrane | phosphatase |
| RDH | | 1.0 | Cytoplasm | group |
| RDH10 | retinol dehydrogenase 10 (all-trans) | 1.5 | Nucleus | enzyme |
| Secretase gamma | | 1.0 | Plasma Membrane | complex |
| SLC6A17 | solute carrier family 6 member 17 | 0.6 | Cytoplasm | transporter |
| Sod | | 1.0 | Cytoplasm | group |
| TBC1D8 | TBC1 domain family member 8 | 0.6 | Plasma Membrane | other |
| TMF1 | TATA element modulatory factor 1 | 1.5 | Cytoplasm | transcription regulator |
| tyrosine kinase | | 1.0 | Other | group |
| Vegf | | 1.0 | Extracellular Space | group |

# 4 hrs of stretching, network 4

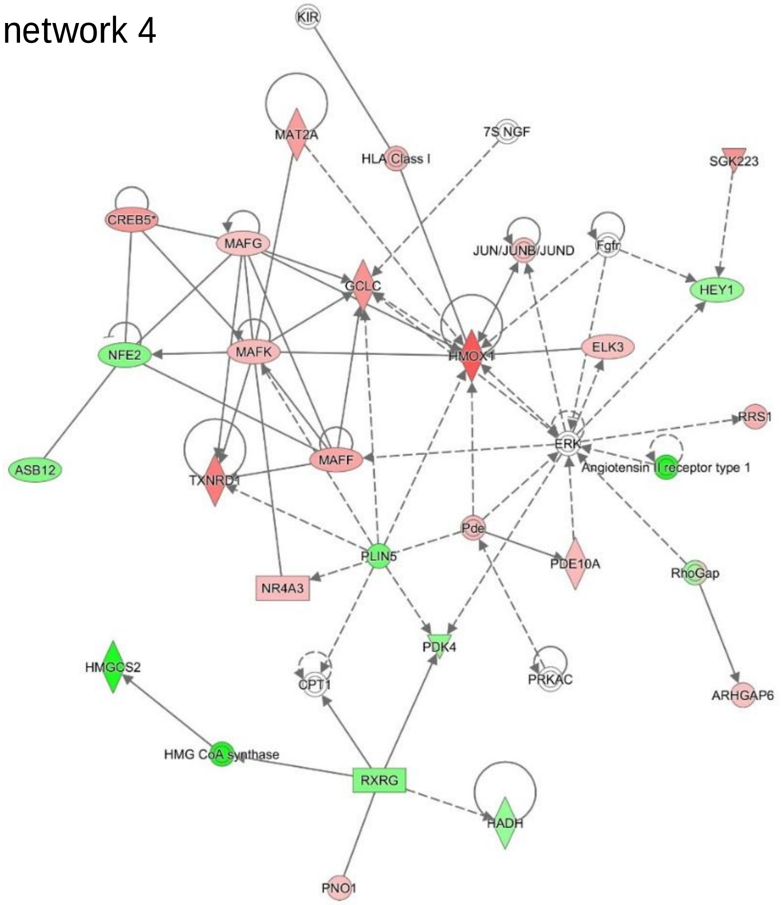


| Symbol | Gene Name | Fold change | Location | Family |
|---|---|---|---|---|
| 7S NGF | | | Other | complex |
| Angiotensin II receptor type 1 | | | Plasma Membrane | group |
| ARHGAP6 | Rho GTPase activating protein 6 | 1.5 | Cytoplasm | other |
| ASB12 | ankyrin repeat and SOCS box containing 12 | 0.6 | Nucleus | transcription regulator |
| CPT1 | | | Cytoplasm | group |
| CREB5 | cAMP responsive element binding protein 5 | 2.0 | Nucleus | transcription regulator |
| ELK3 | ELK3. ETS transcription factor | 1.6 | Nucleus | transcription regulator |
| ERK | | | Other | group |
| Fgfr | | | Plasma Membrane | group |
| GCLC | glutamate-cysteine ligase catalytic subunit | 2.1 | Cytoplasm | enzyme |
| HADH | hydroxyacyl-CoA dehydrogenase | 0.6 | Cytoplasm | enzyme |
| HEY1 | hes related family bHLH transcription factor with YRPW motif 1 | 0.6 | Nucleus | transcription regulator |
| HLA Class I | | | Plasma Membrane | complex |
| HMG CoA synthase | | | Nucleus | group |
| HMGCS2 | 3-hydroxy-3-methylglutaryl-CoA synthase 2 | 0.3 | Cytoplasm | enzyme |
| HMOX1 | heme oxygenase 1 | 3.2 | Cytoplasm | enzyme |
| JUN/JUNB/JUND | | | Nucleus | group |
| KIR | | | Other | group |
| MAFF | MAF bZIP transcription factor F | 1.9 | Nucleus | transcription regulator |
| MAFG | MAF bZIP transcription factor G | 1.5 | Nucleus | transcription regulator |
| MAFK | MAF bZIP transcription factor K | 1.6 | Nucleus | transcription regulator |
| MAT2A | methionine adenosyltransferase 2A | 2.0 | Cytoplasm | enzyme |
| NFE2 | nuclear factor. erythroid 2 | 0.6 | Nucleus | transcription regulator |
| NR4A3 | nuclear receptor subfamily 4 group A member 3 | 1.6 | Nucleus | ligand-dependent nuclear receptor |
| Pde | | | Cytoplasm | group |
| PDE10A | phosphodiesterase 10A | 1.6 | Cytoplasm | enzyme |
| PDK4 | pyruvate dehydrogenase kinase 4 | 0.6 | Cytoplasm | kinase |
| PLIN5 | perilipin 5 | 0.5 | Plasma Membrane | other |
| PNO1 | partner of NOB1 homolog | 1.5 | Nucleus | other |
| PRKAC | PRKAC | | Cytoplasm | group |
| RhoGap | | | Cytoplasm | group |
| RRS1 | ribosome biogenesis regulator homolog | 1.8 | Nucleus | other |
| RXRG | retinoid X receptor gamma | 0.6 | Nucleus | ligand-dependent nuclear receptor |
| SGK223 | PEAK1 related kinase activating pseudokinase 1 | 2.1 | Nucleus | kinase |
| TXNRD1 | thioredoxin reductase 1 | 2.5 | Cytoplasm | enzyme |

# 4 hrs of stretching, network 5

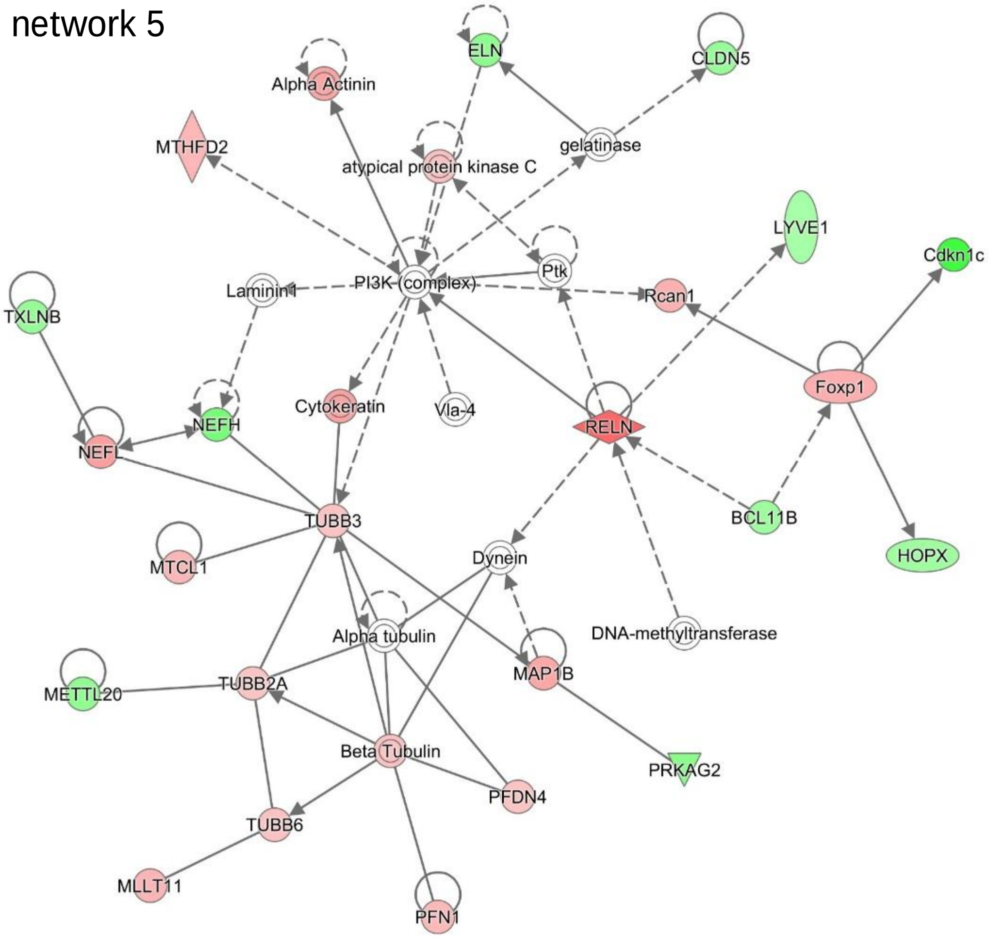


| Symbol | Gene Name | Fold change | Location | Family |
|---|---|---|---|---|
| Alpha Actinin | | | Cytoplasm | group |
| Alpha tubulin | | | Cytoplasm | group |
| atypical protein kinase C | | | Cytoplasm | group |
| BCL11B | B-cell CLL/lymphoma 11B | 0.6 | Nucleus | other |
| Beta Tubulin | | | Other | group |
| Cdkn1c | cyclin-dependent kinase inhibitor 1C (P57) | 0.4 | Nucleus | other |
| CLDN5 | claudin 5 | 0.6 | Plasma Membrane | other |
| Cytokeratin | | | Other | group |
| DNA-methyltransferase | | | Other | group |
| Dynein | | | Cytoplasm | complex |
| ELN | elastin | 0.6 | Extracellular Space | other |
| Foxp1 | forkhead box P1 | 1.8 | Nucleus | transcription regulator |
| gelatinase | | | Nucleus | group |
| HOPX | HOP homeobox | 0.6 | Nucleus | transcription regulator |
| Laminin1 | | | Other | complex |
| LYVE1 | lymphatic vessel endothelial hyaluronan receptor 1 | 0.7 | Plasma Membrane | transmembrane receptor |
| MAP1B | microtubule associated protein 1B | 1.8 | Cytoplasm | other |
| METTL20 | electron transfer flavoprotein beta subunit lysine methyltransferase | 0.6 | Cytoplasm | other |
| MLLT11 | myeloid/lymphoid or mixed-lineage leukemia; translocated to. 11 | 1.7 | Cytoplasm | other |
| MTCL1 | microtubule crosslinking factor 1 | 1.7 | Cytoplasm | other |
| MTHFD2 | methylenetetrahydrofolate dehydrogenase (NADP+ dependent) 2. methenyltetrahydrofolate cyclohydrolase | 1.6 | Cytoplasm | enzyme |
| NEFH | neurofilament heavy polypeptide | 0.5 | Cytoplasm | other |
| NEFL | neurofilament. light polypeptide | 1.9 | Cytoplasm | other |
| PFDN4 | prefoldin subunit 4 | 1.5 | Cytoplasm | other |
| PFN1 | profilin 1 | 1.6 | Cytoplasm | other |
| PI3K (complex) | | | Cytoplasm | complex |
| PRKAG2 | protein kinase AMP-activated non-catalytic subunit gamma 2 | 0.6 | Cytoplasm | kinase |
| Ptk | | | Cytoplasm | group |
| Rcan1 | regulator of calcineurin 1 | 1.7 | Nucleus | other |
| RELN | reelin | 2.9 | Extracellular Space | peptidase |
| TUBB2A | tubulin beta 2A class IIa | 1.5 | Cytoplasm | other |
| TUBB3 | tubulin beta 3 class III | 1.5 | Cytoplasm | other |
| TUBB6 | tubulin beta 6 class V | 1.5 | Cytoplasm | other |
| TXLNB | taxilin beta | 0.6 | Cytoplasm | other |
| Vla-4 | | | Plasma Membrane | complex |

12 hrs of stretching, network 1

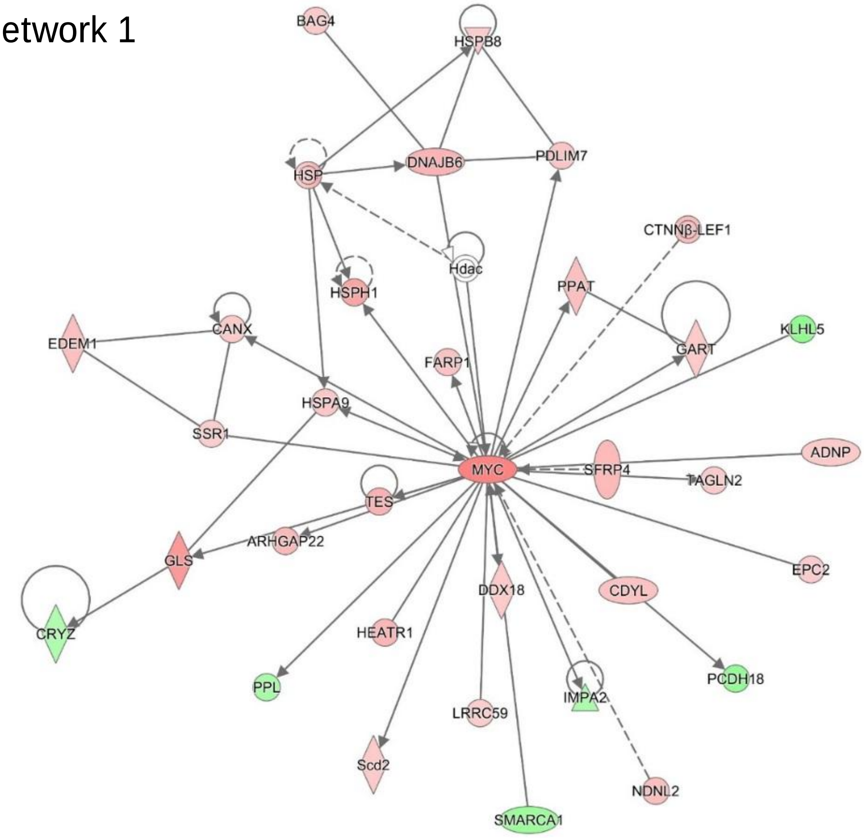


| Symbol | Gene Name | Fold change | Location | Family |
|---|---|---|---|---|
| ADNP | activity dependent neuroprotector homeobox | 1.5 | Nucleus | transcription regulator |
| ARHGAP22 | Rho GTPase activating protein 22 | 1.8 | Cytoplasm | other |
| BAG4 | BCL2 associated athanogene 4 | 1.5 | Cytoplasm | other |
| CANX | calnexin | 1.6 | Cytoplasm | other |
| CDYL | chromodomain Y-like | 1.6 | Nucleus | transcription regulator |
| CRYZ | crystallin zeta | 0.7 | Cytoplasm | enzyme |
| CTNN**β**-LEF1 | | | Nucleus | complex |
| DDX18 | DEAD-box helicase 18 | 1.5 | Nucleus | enzyme |
| DNAJB6 | DnaJ heat shock protein family (Hsp40) member B6 | 1.9 | Nucleus | transcription regulator |
| EDEM1 | ER degradation enhancing alpha-mannosidase like protein 1 | 1.7 | Cytoplasm | enzyme |
| EPC2 | enhancer of polycomb homolog 2 | 1.5 | Other | other |
| FARP1 | FERM. ARH/RhoGEF and pleckstrin domain protein 1 | 1.6 | Plasma Membrane | other |
| GART | phosphoribosylglycinamide formyltransferase. phosphoribosylglycinamide synthetase. phosphoribosylaminoimidazole synthetase | 1.5 | Cytoplasm | enzyme |
| GLS | glutaminase | 2.3 | Cytoplasm | enzyme |
| Hdac | | | Nucleus | group |
| HEATR1 | HEAT repeat containing 1 | 1.8 | Nucleus | other |
| HSP | | | Cytoplasm | group |
| HSPA9 | heat shock protein family A (Hsp70) member 9 | 1.6 | Cytoplasm | other |
| HSPB8 | heat shock protein family B (small) member 8 | 1.5 | Cytoplasm | kinase |
| HSPH1 | heat shock protein family H (Hsp110) member 1 | 2.1 | Cytoplasm | other |
| IMPA2 | inositol monophosphatase 2 | 0.6 | Cytoplasm | phosphatase |
| KLHL5 | kelch like family member 5 | 0.5 | Extracellular Space | other |
| LRRC59 | leucine rich repeat containing 59 | 1.5 | Cytoplasm | other |
| MYC | v-myc avian myelocytomatosis viral oncogene homolog | 2.8 | Nucleus | transcription regulator |
| NDNL2 | NSE3 homolog. SMC5-SMC6 complex component | 1.7 | Nucleus | other |
| PCDH18 | protocadherin 18 | 0.6 | Extracellular Space | other |
| PDLIM7 | PDZ and LIM domain 7 | 1.6 | Cytoplasm | other |
| PPAT | phosphoribosyl pyrophosphate amidotransferase | 1.7 | Cytoplasm | enzyme |
| PPL | periplakin | 0.6 | Cytoplasm | other |
| Scd2 | stearoyl-Coenzyme A desaturase 2 | 1.5 | Cytoplasm | enzyme |
| SFRP4 | secreted frizzled related protein 4 | 1.8 | Plasma Membrane | transmembrane receptor |
| SMARCA1 | SWI/SNF related. matrix associated. actin dependent regulator of chromatin. subfamily a. member 1 | 0.6 | Nucleus | transcription regulator |
| SSR1 | signal sequence receptor subunit 1 | 1.5 | Cytoplasm | other |
| TAGLN2 | transgelin 2 | 1.5 | Cytoplasm | other |
| TES | testin LIM domain protein | 1.9 | Plasma Membrane | other |

12 hrs of stretching, network 2

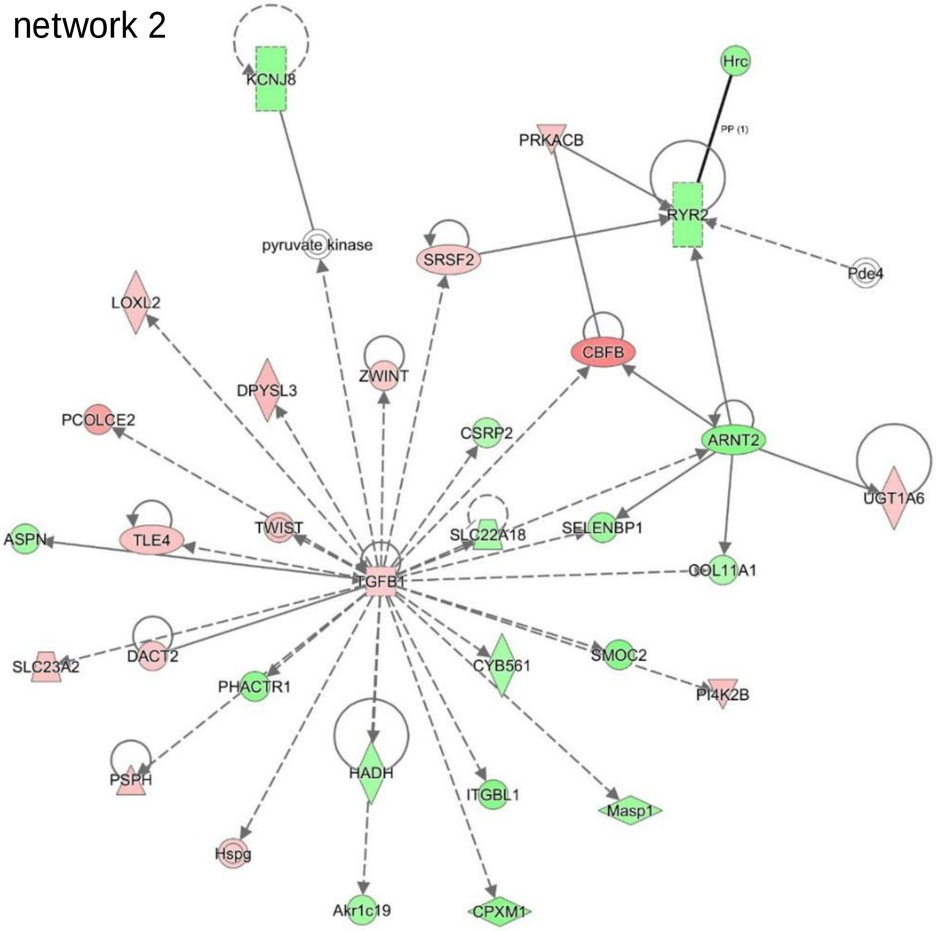


| Symbol | Gene Name | Fold change | Location | Family |
|---|---|---|---|---|
| Akr1c19 | aldo-keto reductase family 1. member C19 | 0.6 | Other | other |
| ARNT2 | aryl hydrocarbon receptor nuclear translocator 2 | 0.5 | Nucleus | transcription regulator |
| ASPN | asporin | 0.6 | Extracellular Space | other |
| CBFB | core-binding factor beta subunit | 2.8 | Nucleus | transcription regulator |
| COL11A1 | collagen type XI alpha 1 chain | 0.7 | Extracellular Space | other |
| CPXM1 | carboxypeptidase X. M14 family member 1 | 0.5 | Extracellular Space | peptidase |
| CSRP2 | cysteine and glycine rich protein 2 | 0.7 | Nucleus | other |
| CYB561 | cytochrome b561 | 0.6 | Cytoplasm | enzyme |
| DACT2 | dishevelled binding antagonist of beta catenin 2 | 1.6 | Cytoplasm | other |
| DPYSL3 | dihydropyrimidinase like 3 | 1.8 | Cytoplasm | enzyme |
| HADH | hydroxyacyl-CoA dehydrogenase | 0.6 | Cytoplasm | enzyme |
| Hrc | histidine rich calcium binding protein | 0.6 | Cytoplasm | other |
| Hspg | | | Plasma Membrane | group |
| ITGBL1 | integrin subunit beta like 1 | 0.5 | Other | other |
| KCNJ8 | potassium voltage-gated channel subfamily J member 8 | 0.6 | Plasma Membrane | ion channel |
| LOXL2 | lysyl oxidase like 2 | 1.6 | Extracellular Space | enzyme |
| Masp1 | mannan-binding lectin serine peptidase 1 | 0.6 | Extracellular Space | peptidase |
| PCOLCE2 | procollagen C-endopeptidase enhancer 2 | 2.2 | Extracellular Space | other |
| Pde4 | | | Other | group |
| PHACTR1 | phosphatase and actin regulator 1 | 0.5 | Cytoplasm | other |
| PI4K2B | phosphatidylinositol 4-kinase type 2 beta | 1.7 | Cytoplasm | kinase |
| PRKACB | protein kinase cAMP-activated catalytic subunit beta | 1.6 | Cytoplasm | kinase |
| PSPH | phosphoserine phosphatase | 1.7 | Cytoplasm | phosphatase |
| pyruvate kinase | | | Cytoplasm | group |
| RYR2 | ryanodine receptor 2 | 0.6 | Plasma Membrane | ion channel |
| SELENBP1 | selenium binding protein 1 | 0.6 | Cytoplasm | other |
| SLC22A18 | solute carrier family 22 member 18 | 0.6 | Plasma Membrane | transporter |
| SLC23A2 | solute carrier family 23 member 2 | 1.6 | Plasma Membrane | transporter |
| SMOC2 | SPARC related modular calcium binding 2 | 0.5 | Extracellular Space | other |
| SRSF2 | serine and arginine rich splicing factor 2 | 1.5 | Nucleus | transcription regulator |
| TGFB1 | transforming growth factor beta 1 | 1.5 | Extracellular Space | growth factor |
| TLE4 | transducin like enhancer of split 4 | 1.6 | Nucleus | transcription regulator |
| TWIST | | | Other | group |
| UGT1A6 | UDP glucuronosyltransferase family 1 member A6 | 1.6 | Cytoplasm | enzyme |
| ZWINT | ZW10 interacting kinetochore protein | 1.6 | Nucleus | other |

# 12 hrs of stretching, network 3

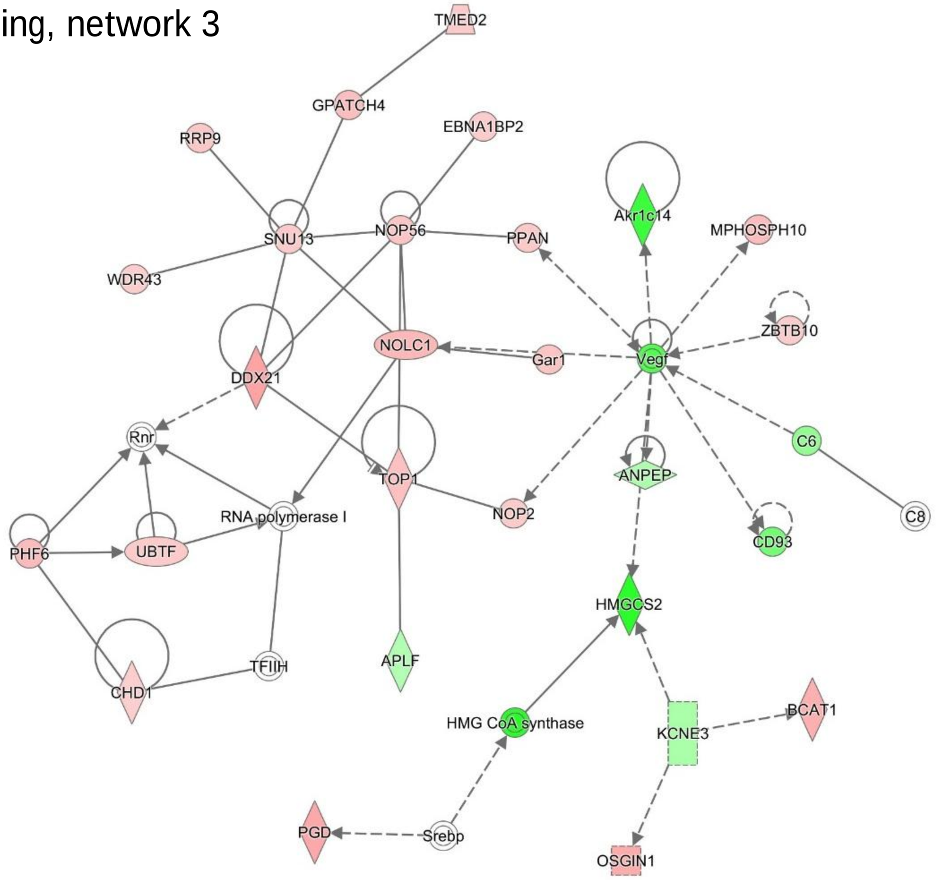


| Symbol | Gene Name | Fold change | Location | Family |
|---|---|---|---|---|
| Akr1c14 | aldo-keto reductase family 1. member C14 | 0.4 | Cytoplasm | enzyme |
| ANPEP | alanyl aminopeptidase. membrane | 0.7 | Plasma Membrane | peptidase |
| APLF | aprataxin and PNKP like factor | 0.7 | Cytoplasm | enzyme |
| BCAT1 | branched chain amino acid transaminase 1 | 1.9 | Cytoplasm | enzyme |
| C6 | complement C6 | 0.6 | Extracellular Space | other |
| C8 | | | Cytoplasm | complex |
| CD93 | CD93 molecule | 0.5 | Plasma Membrane | other |
| CHD1 | chromodomain helicase DNA binding protein 1 | 1.5 | Nucleus | enzyme |
| DDX21 | DExD-box helicase 21 | 2.1 | Nucleus | enzyme |
| EBNA1BP2 | EBNA1 binding protein 2 | 1.5 | Nucleus | other |
| Gar1 | GAR1 ribonucleoprotein | 1.6 | Nucleus | other |
| GPATCH4 | G-patch domain containing 4 | 1.7 | Other | other |
| HMG CoA synthase | | | Nucleus | group |
| HMGCS2 | 3-hydroxy-3-methylglutaryl-CoA synthase 2 | 0.3 | Cytoplasm | enzyme |
| KCNE3 | potassium voltage-gated channel subfamily E regulatory subunit 3 | 0.6 | Plasma Membrane | ion channel |
| MPHOSPH10 | M-phase phosphoprotein 10 | 1.7 | Nucleus | other |
| NOLC1 | nucleolar and coiled-body phosphoprotein 1 | 1.8 | Nucleus | transcription regulator |
| NOP2 | NOP2 nucleolar protein | 1.5 | Nucleus | other |
| NOP56 | NOP56 ribonucleoprotein | 1.6 | Nucleus | other |
| OSGIN1 | oxidative stress induced growth inhibitor 1 | 2.0 | Other | growth factor |
| PGD | phosphogluconate dehydrogenase | 2.1 | Cytoplasm | enzyme |
| PHF6 | PHD finger protein 6 | 1.8 | Nucleus | other |
| PPAN | peter pan homolog (Drosophila) | 1.6 | Nucleus | other |
| RNA polymerase I | | | Nucleus | complex |
| Rnr | | | Other | group |
| RRP9 | ribosomal RNA processing 9. small subunit (SSU) processome component. homolog (yeast) | 1.6 | Nucleus | other |
| SNU13 | SNU13 homolog. small nuclear ribonucleoprotein (U4/U6.U5) | 1.5 | Nucleus | other |
| Srebp | | | Other | group |
| TFIIH | | | Nucleus | complex |
| TMED2 | transmembrane p24 trafficking protein 2 | 1.6 | Cytoplasm | transporter |
| TOP1 | topoisomerase (DNA) I | 1.7 | Nucleus | enzyme |
| UBTF | upstream binding transcription factor. RNA polymerase I | 1.5 | Nucleus | transcription regulator |
| Vegf | | | Extracellular Space | group |
| WDR43 | WD repeat domain 43 | 1.5 | Nucleus | other |
| ZBTB10 | zinc finger and BTB domain containing 10 | 1.5 | Nucleus | other |

# 12 hrs of stretching, network 4

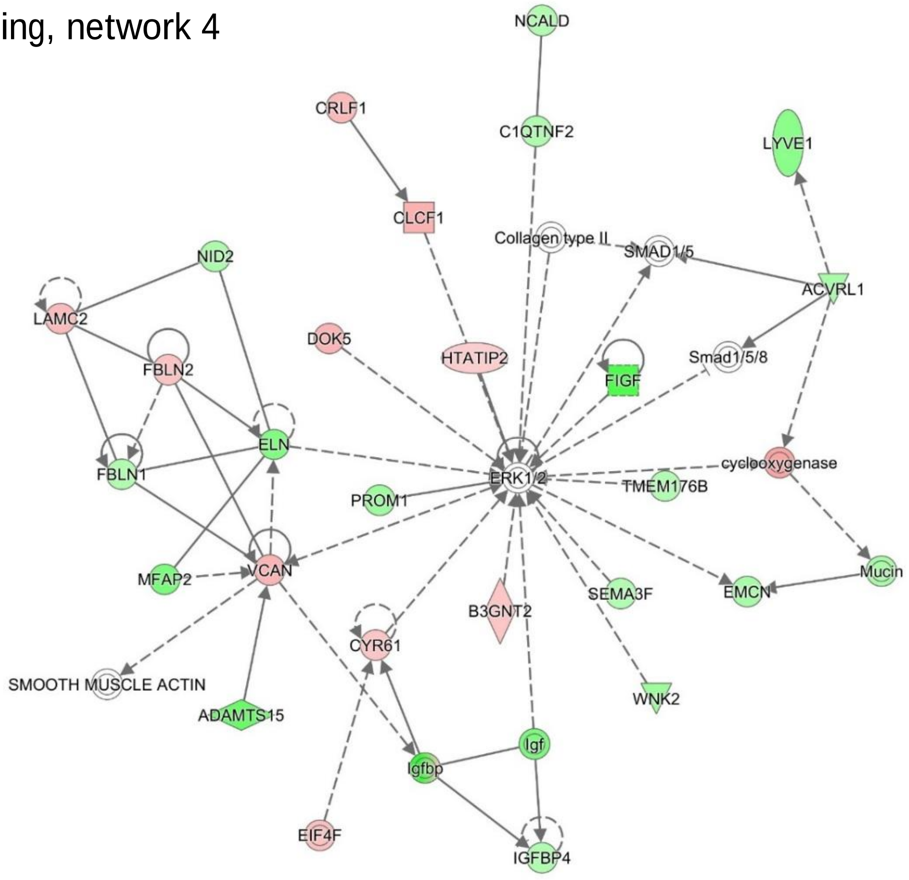


| Symbol | Gene Name | Fold change | Location | Family |
|---|---|---|---|---|
| ACVRL1 | activin A receptor like type 1 | 0.6 | Plasma Membrane | kinase |
| ADAMTS15 | ADAM metallopeptidase with thrombospondin type 1 motif 15 | 0.4 | Extracellular Space | peptidase |
| B3GNT2 | UDP-GlcNAc:betaGal beta-1.3-N-acetylglucosaminyltransferase 2 | 1.6 | Cytoplasm | enzyme |
| C1QTNF2 | C1q and tumor necrosis factor related protein 2 | 0.6 | Extracellular Space | other |
| CLCF1 | cardiotrophin-like cytokine factor 1 | 1.9 | Extracellular Space | cytokine |
| Collagen type II | | | Other | complex |
| CRLF1 | cytokine receptor like factor 1 | 1.8 | Extracellular Space | other |
| cyclooxygenase | | | Cytoplasm | group |
| CYR61 | cysteine rich angiogenic inducer 61 | 1.6 | Extracellular Space | other |
| DOK5 | docking protein 5 | 1.9 | Plasma Membrane | other |
| EIF4F | | | Cytoplasm | complex |
| ELN | elastin | 0.5 | Extracellular Space | other |
| EMCN | endomucin | 0.6 | Extracellular Space | other |
| ERK1/2 | | | Cytoplasm | group |
| FBLN1 | fibulin 1 | 0.7 | Extracellular Space | other |
| FBLN2 | fibulin 2 | 1.6 | Extracellular Space | other |
| FIGF | vascular endothelial growth factor D | 0.4 | Extracellular Space | growth factor |
| HTATIP2 | HIV-1 Tat interactive protein 2 | 1.5 | Nucleus | transcription regulator |
| Igf | | | Extracellular Space | group |
| Igfbp | | | Other | group |
| IGFBP4 | insulin like growth factor binding protein 4 | 0.7 | Extracellular Space | other |
| LAMC2 | laminin subunit gamma 2 | 1.8 | Extracellular Space | other |
| LYVE1 | lymphatic vessel endothelial hyaluronan receptor 1 | 0.5 | Plasma Membrane | transmembrane receptor |
| MFAP2 | microfibrillar associated protein 2 | 0.5 | Extracellular Space | other |
| Mucin | | | Other | group |
| NCALD | neurocalcin delta | 0.7 | Cytoplasm | other |
| NID2 | nidogen 2 | 0.6 | Extracellular Space | other |
| PROM1 | prominin 1 | 0.6 | Plasma Membrane | other |
| SEMA3F | semaphorin 3F | 0.7 | Extracellular Space | other |
| SMAD1/5 | | | Cytoplasm | group |
| Smad1/5/8 | | | Cytoplasm | group |
| SMOOTH MUSCLE ACTIN | | | Other | group |
| TMEM176B | transmembrane protein 176B | 0.7 | Other | other |
| VCAN | versican | 1.8 | Extracellular Space | other |
| WNK2 | WNK lysine deficient protein kinase 2 | 0.6 | Cytoplasm | kinase |

# 12 hrs of stretching, network 5

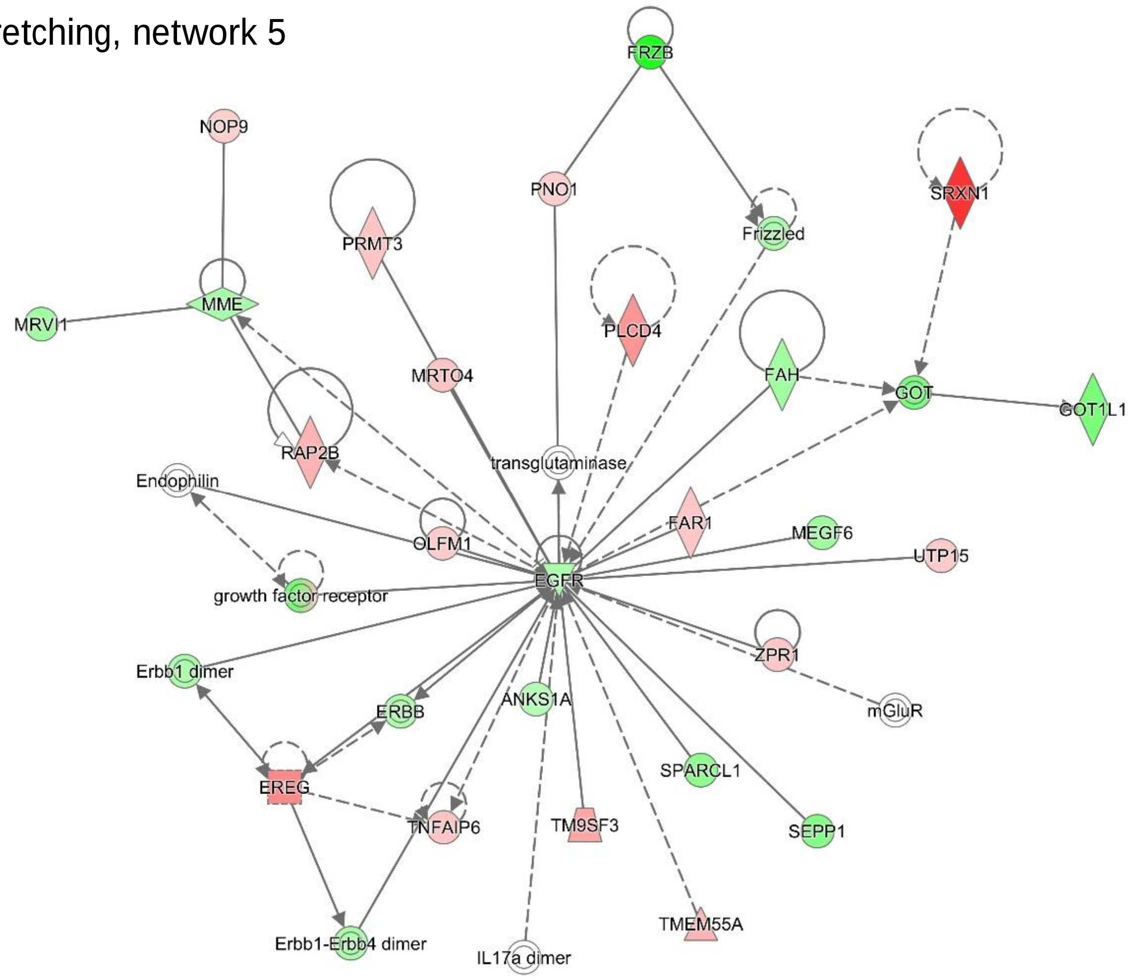


| Symbol | Gene Name | Fold change | Location | Family |
|---|---|---|---|---|
| ANKS1A | ankyrin repeat and sterile alpha motif domain containing 1A | 0.7 | Cytoplasm | other |
| EGFR | epidermal growth factor receptor | 0.6 | Plasma Membrane | kinase |
| Endophilin | | | Cytoplasm | group |
| ERBB | | | Plasma Membrane | group |
| Erbb1 dimer | | | Plasma Membrane | complex |
| Erbb1-Erbb4 dimer | | | Plasma Membrane | complex |
| EREG | epiregulin | 2.7 | Extracellular Space | growth factor |
| FAH | fumarylacetoacetate hydrolase | 0.6 | Cytoplasm | enzyme |
| FAR1 | fatty acyl-CoA reductase 1 | 1.6 | Cytoplasm | enzyme |
| Frizzled | | | Plasma Membrane | group |
| FRZB | frizzled-related protein | 0.3 | Extracellular Space | other |
| GOT | | | Other | group |
| GOT1L1 | glutamic-oxaloacetic transaminase 1-like 1 | 0.5 | Other | enzyme |
| growth factor receptor | | | Plasma Membrane | group |
| IL17a dimer | | | Extracellular Space | complex |
| MEGF6 | multiple EGF like domains 6 | 0.6 | Cytoplasm | other |
| mGluR | | | Plasma Membrane | group |
| MME | membrane metalloendopeptidase | 0.6 | Plasma Membrane | peptidase |
| MRTO4 | MRT4 homolog. ribosome maturation factor | 1.6 | Cytoplasm | other |
| MRVI1 | murine retrovirus integration site 1 homolog | 0.6 | Cytoplasm | other |
| NOP9 | NOP9 nucleolar protein | 1.5 | Nucleus | other |
| OLFM1 | olfactomedin 1 | 1.5 | Cytoplasm | other |
| PLCD4 | phospholipase C delta 4 | 2.4 | Cytoplasm | enzyme |
| PNO1 | partner of NOB1 homolog | 1.5 | Nucleus | other |
| PRMT3 | protein arginine methyltransferase 3 | 1.6 | Nucleus | enzyme |
| RAP2B | RAP2B. member of RAS oncogene family | 1.9 | Plasma Membrane | enzyme |
| SEPP1 | selenoprotein P | 0.5 | Extracellular Space | other |
| SPARCL1 | SPARC like 1 | 0.5 | Extracellular Space | other |
| SRXN1 | sulfiredoxin 1 | 6.3 | Cytoplasm | enzyme |
| TM9SF3 | transmembrane 9 superfamily member 3 | 2.1 | Cytoplasm | transporter |
| TMEM55A | transmembrane protein 55A | 1.9 | Cytoplasm | phosphatase |
| TNFAIP6 | TNF alpha induced protein 6 | 1.6 | Extracellular Space | other |
| transglutaminase | | | Other | group |
| UTP15 | UTP15. small subunit processome component | 1.6 | Nucleus | other |
| ZPR1 | ZPR1 zinc finger | 1.6 | Nucleus | other |

# 24 hrs of stretching, network 1

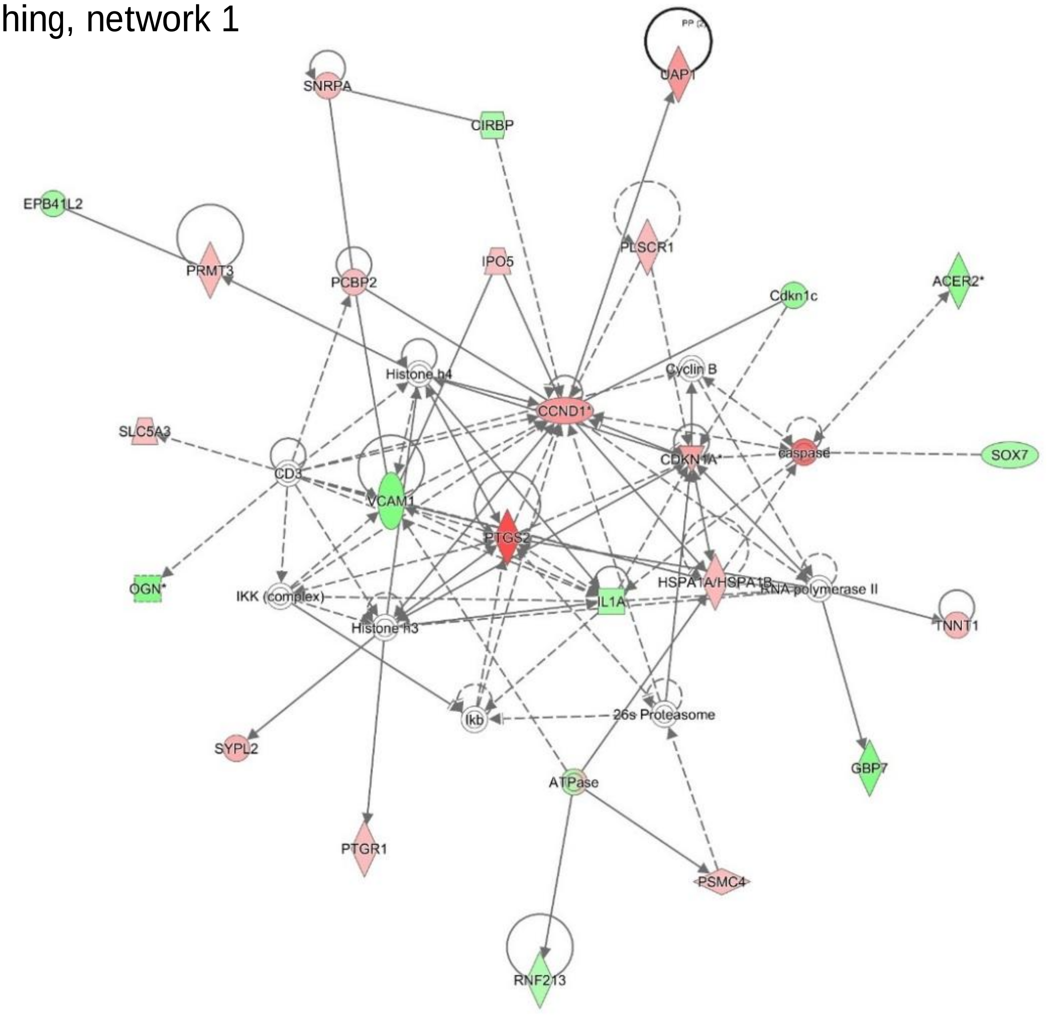


| Symbol | Gene Name | Fold change | Location | Family |
|---|---|---|---|---|
| 26s Proteasome | | | Cytoplasm | complex |
| ACER2 | alkaline ceramidase 2 | 0.6 | Cytoplasm | enzyme |
| ATPase | | | Other | group |
| caspase | | | Cytoplasm | group |
| CCND1 | cyclin D1 | 2.0 | Nucleus | transcription regulator |
| CD3 | | | Plasma Membrane | complex |
| CDKN1A | cyclin dependent kinase inhibitor 1A | 1.9 | Nucleus | kinase |
| Cdkn1c | cyclin-dependent kinase inhibitor 1C (P57) | 0.6 | Nucleus | other |
| CIRBP | cold inducible RNA binding protein | 0.6 | Nucleus | translation regulator |
| Cyclin B | | | Cytoplasm | group |
| EPB41L2 | erythrocyte membrane protein band 4.1 like 2 | 0.6 | Plasma Membrane | other |
| GBP7 | guanylate binding protein 7 | 0.5 | Cytoplasm | enzyme |
| Histone h3 | | | Nucleus | group |
| Histone h4 | | | Nucleus | group |
| HSPA1A/HSPA1B | heat shock protein family A (Hsp70) member 1A | 1.6 | Cytoplasm | enzyme |
| Ikb | | | Cytoplasm | group |
| IKK (complex) | | | Cytoplasm | complex |
| IL1A | interleukin 1 alpha | 0.6 | Extracellular Space | cytokine |
| IPO5 | importin 5 | 1.5 | Nucleus | transporter |
| OGN | osteoglycin | 0.5 | Extracellular Space | growth factor |
| PCBP2 | poly(rC) binding protein 2 | 1.6 | Nucleus | other |
| PLSCR1 | phospholipid scramblase 1 | 1.6 | Plasma Membrane | enzyme |
| PRMT3 | protein arginine methyltransferase 3 | 1.6 | Nucleus | enzyme |
| PSMC4 | proteasome 26S subunit. ATPase 4 | 1.5 | Nucleus | peptidase |
| PTGR1 | prostaglandin reductase 1 | 1.5 | Cytoplasm | enzyme |
| PTGS2 | prostaglandin-endoperoxide synthase 2 | 3.3 | Cytoplasm | enzyme |
| RNA polymerase II | | | Nucleus | complex |
| RNF213 | ring finger protein 213 | 0.7 | Cytoplasm | enzyme |
| SLC5A3 | solute carrier family 5 member 3 | 1.5 | Plasma Membrane | transporter |
| SNRPA | small nuclear ribonucleoprotein polypeptide A | 1.6 | Nucleus | other |
| SOX7 | SRY-box 7 | 0.6 | Nucleus | transcription regulator |
| SYPL2 | synaptophysin like 2 | 1.7 | Other | other |
| TNNT1 | troponin T1. slow skeletal type | 1.6 | Cytoplasm | other |
| UAP1 | UDP-N-acetylglucosamine pyrophosphorylase 1 | 2.0 | Nucleus | enzyme |
| VCAM1 | vascular cell adhesion molecule 1 | 0.5 | Plasma Membrane | transmembrane receptor |

# 24 hrs of stretching, network 2

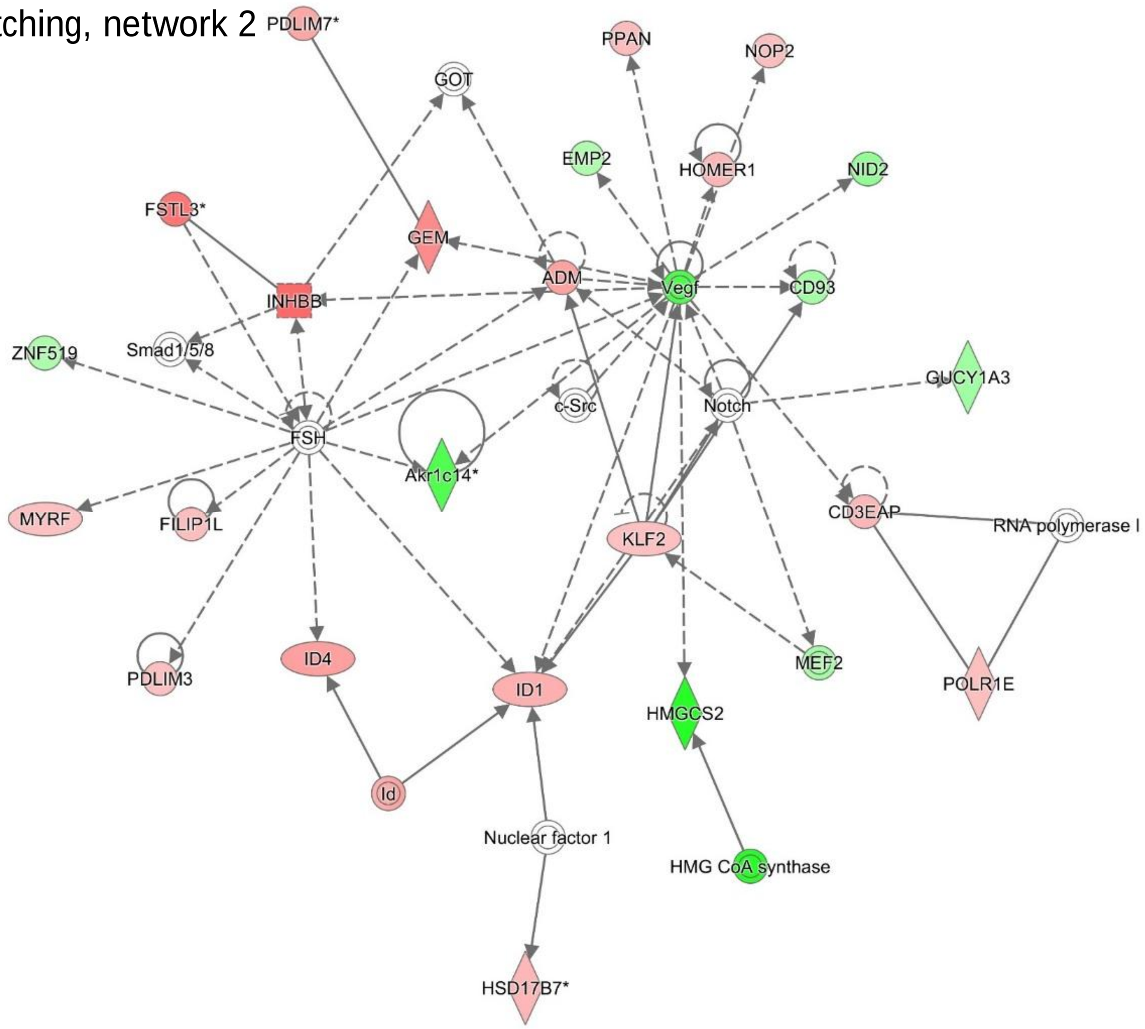


| Symbol | Gene Name | Fold change | Location | Family |
|---|---|---|---|---|
| ADM | adrenomedullin | 1.9 | Extracellular Space | other |
| Akr1c14 | aldo-keto reductase family 1. member C14 | 0.4 | Cytoplasm | enzyme |
| c-Src | | | Cytoplasm | group |
| CD3EAP | CD3e molecule associated protein | 1.6 | Nucleus | other |
| CD93 | CD93 molecule | 0.6 | Plasma Membrane | other |
| EMP2 | epithelial membrane protein 2 | 0.7 | Plasma Membrane | other |
| FILIP1L | filamin A interacting protein 1 like | 1.5 | Nucleus | other |
| FSH | | | Plasma Membrane | complex |
| FSTL3 | follistatin like 3 | 2.5 | Extracellular Space | other |
| GEM | GTP binding protein overexpressed in skeletal muscle | 2.1 | Plasma Membrane | enzyme |
| GOT | | | Other | group |
| GUCY1A3 | guanylate cyclase 1 soluble subunit alpha | 0.6 | Cytoplasm | enzyme |
| HMG CoA synthase | | | Nucleus | group |
| HMGCS2 | 3-hydroxy-3-methylglutaryl-CoA synthase 2 | 0.3 | Cytoplasm | enzyme |
| HOMER1 | homer scaffolding protein 1 | 1.6 | Plasma Membrane | other |
| HSD17B7 | hydroxysteroid 17-beta dehydrogenase 7 | 1.6 | Cytoplasm | enzyme |
| Id | | | Other | group |
| ID1 | inhibitor of DNA binding 1. HLH protein | 1.7 | Nucleus | transcription regulator |
| ID4 | inhibitor of DNA binding 4. HLH protein | 1.9 | Nucleus | transcription regulator |
| INHBB | inhibin beta B subunit | 2.7 | Extracellular Space | growth factor |
| KLF2 | Kruppel like factor 2 | 1.5 | Nucleus | transcription regulator |
| MEF2 | | | Nucleus | group |
| MYRF | myelin regulatory factor | 1.5 | Nucleus | transcription regulator |
| NID2 | nidogen 2 | 0.6 | Extracellular Space | other |
| NOP2 | NOP2 nucleolar protein | 1.5 | Nucleus | other |
| Notch | | | Plasma Membrane | group |
| Nuclear factor 1 | | | Nucleus | group |
| PDLIM3 | PDZ and LIM domain 3 | 1.5 | Cytoplasm | other |
| PDLIM7 | PDZ and LIM domain 7 | 1.8 | Cytoplasm | other |
| POLR1E | RNA polymerase I subunit E | 1.5 | Nucleus | enzyme |
| PPAN | peter pan homolog (Drosophila) | 1.5 | Nucleus | other |
| RNA polymerase I | | | Nucleus | complex |
| Smad1/5/8 | | | Cytoplasm | group |
| Vegf | | | Extracellular Space | group |
| ZNF519 | zinc finger protein 519 | 0.6 | Nucleus | other |

24 hrs of stretching, network 3

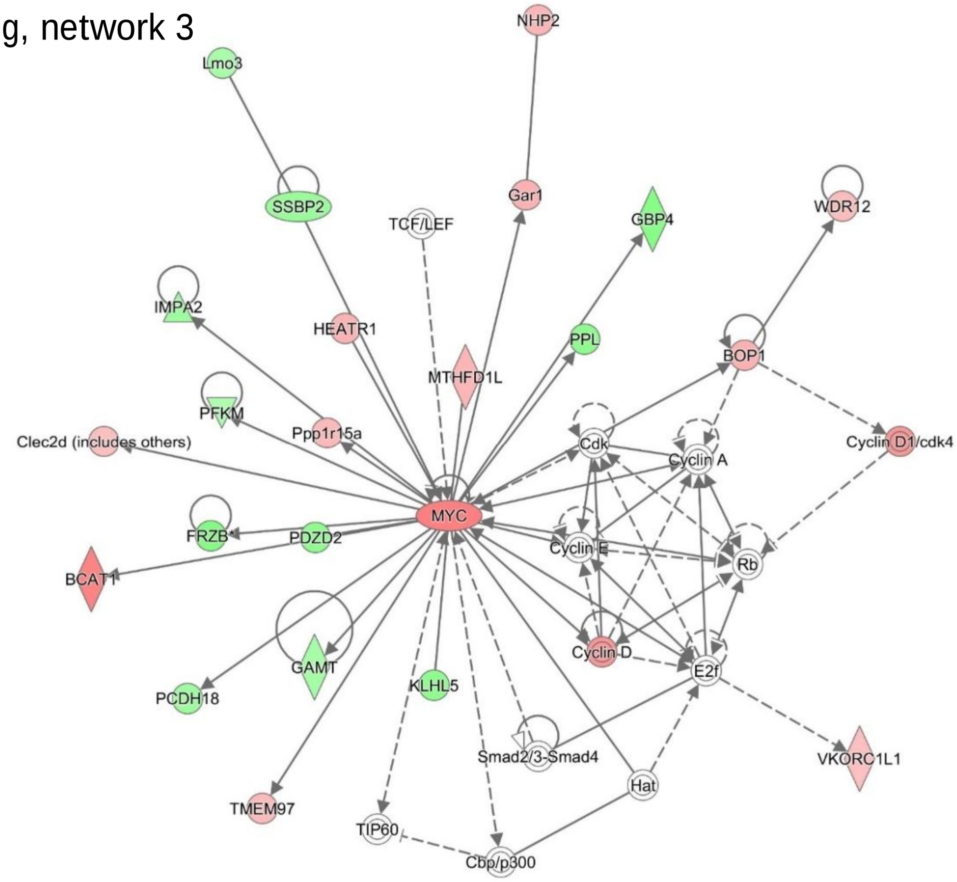


| Symbol | Gene Name | Fold change | Location | Family |
|---|---|---|---|---|
| BCAT1 | branched chain amino acid transaminase 1 | 2.3 | Cytoplasm | enzyme |
| BOP1 | block of proliferation 1 | 1.7 | Nucleus | other |
| Cbp/p300 | | | Nucleus | group |
| Cdk | | | Nucleus | group |
| Clec2d (includes others) | C-type lectin domain family 2. member D | 1.5 | Plasma Membrane | other |
| Cyclin A | | | Nucleus | group |
| Cyclin D | | | Nucleus | group |
| Cyclin D1/cdk4 | | | Nucleus | complex |
| Cyclin E | | | Nucleus | group |
| E2f | | | Nucleus | group |
| FRZB | frizzled-related protein | 0.5 | Extracellular Space | other |
| GAMT | guanidinoacetate N-methyltransferase | 0.6 | Cytoplasm | enzyme |
| Gar1 | GAR1 ribonucleoprotein | 1.6 | Nucleus | other |
| GBP4 | guanylate binding protein 4 | 0.5 | Cytoplasm | enzyme |
| Hat | | | Nucleus | complex |
| HEATR1 | HEAT repeat containing 1 | 1.6 | Nucleus | other |
| IMPA2 | inositol monophosphatase 2 | 0.6 | Cytoplasm | phosphatase |
| KLHL5 | kelch like family member 5 | 0.6 | Extracellular Space | other |
| Lmo3 | LIM domain only 3 | 0.6 | Cytoplasm | other |
| MTHFD1L | methylenetetrahydrofolate dehydrogenase (NADP+ dependent) 1-like | 1.6 | Cytoplasm | enzyme |
| MYC | v-myc avian myelocytomatosis viral oncogene homolog | 2.3 | Nucleus | transcription regulator |
| NHP2 | NHP2 ribonucleoprotein | 1.6 | Nucleus | other |
| PCDH18 | protocadherin 18 | 0.6 | Extracellular Space | other |
| PDZD2 | PDZ domain containing 2 | 0.6 | Plasma Membrane | other |
| PFKM | phosphofructokinase. muscle | 0.7 | Cytoplasm | kinase |
| PPL | periplakin | 0.5 | Cytoplasm | other |
| Ppp1r15a | protein phosphatase 1. regulatory subunit 15A | 1.6 | Cytoplasm | other |
| Rb | | | Nucleus | group |
| Smad2/3-Smad4 | | | Nucleus | complex |
| SSBP2 | single stranded DNA binding protein 2 | 0.6 | Nucleus | transcription regulator |
| TCF/LEF | | | Nucleus | group |
| TIP60 | | | Other | complex |
| TMEM97 | transmembrane protein 97 | 1.6 | Extracellular Space | other |
| VKORC1L1 | vitamin K epoxide reductase complex subunit 1 like 1 | 1.5 | Cytoplasm | enzyme |
| WDR12 | WD repeat domain 12 | 1.5 | Cytoplasm | other |

# 24 hrs of stretching, network 4

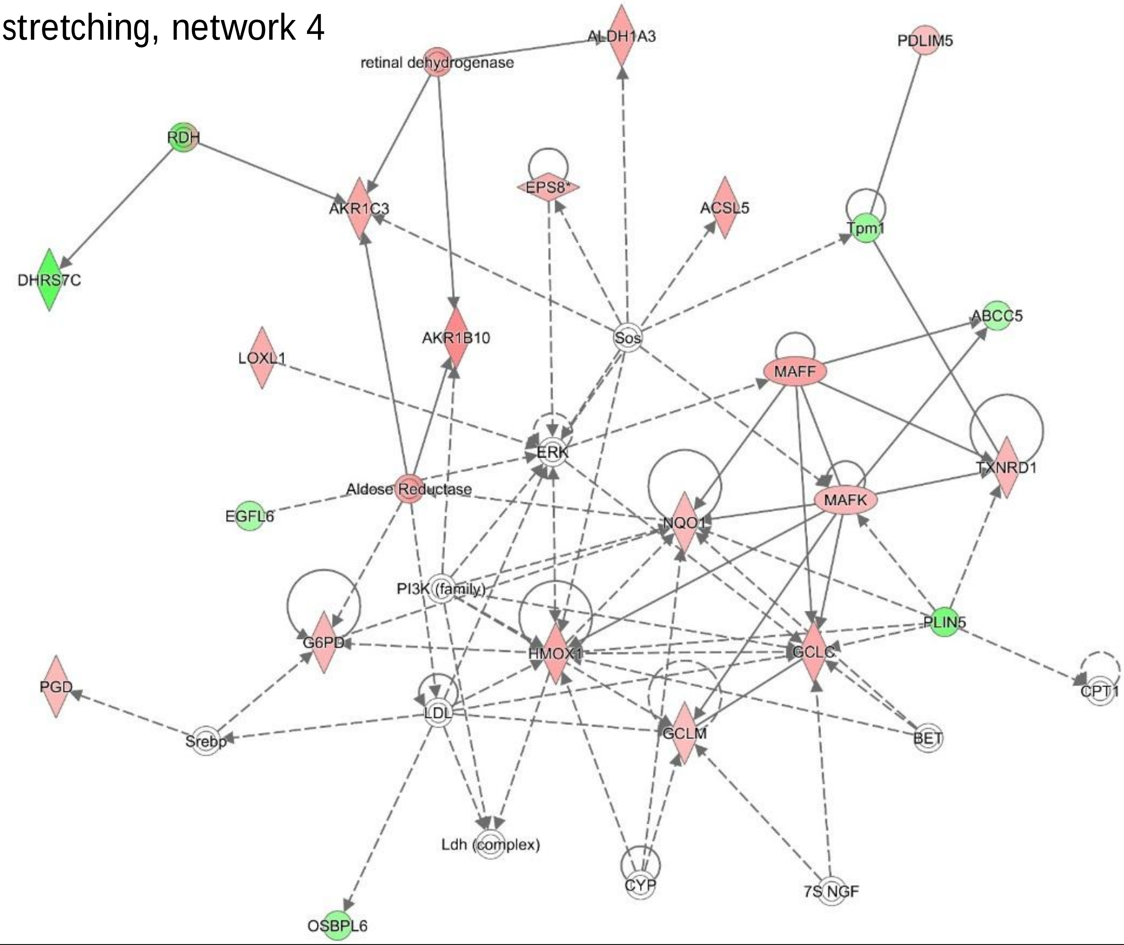


| Symbol | Gene Name | Fold change | Location | Family |
|---|---|---|---|---|
| 7S NGF | | | Other | complex |
| ABCC5 | ATP binding cassette subfamily C member 5 | 0.6 | Plasma Membrane | other |
| ACSL5 | acyl-CoA synthetase long-chain family member 5 | 1.8 | Cytoplasm | enzyme |
| AKR1B10 | aldo-keto reductase family 1 member B10 | 2.2 | Cytoplasm | enzyme |
| AKR1C3 | aldo-keto reductase family 1 member C3 | 1.8 | Cytoplasm | enzyme |
| ALDH1A3 | aldehyde dehydrogenase 1 family member A3 | 1.8 | Cytoplasm | enzyme |
| Aldose Reductase | | | Other | group |
| BET | | | Other | group |
| CPT1 | | | Cytoplasm | group |
| CYP | | | Other | group |
| DHRS7C | dehydrogenase/reductase 7C | 0.4 | Cytoplasm | enzyme |
| EGFL6 | EGF like domain multiple 6 | 0.6 | Extracellular Space | other |
| EPS8 | epidermal growth factor receptor pathway substrate 8 | 1.7 | Plasma Membrane | peptidase |
| ERK | | | Other | group |
| G6PD | glucose-6-phosphate dehydrogenase | 1.6 | Cytoplasm | enzyme |
| GCLC | glutamate-cysteine ligase catalytic subunit | 1.8 | Cytoplasm | enzyme |
| GCLM | glutamate-cysteine ligase modifier subunit | 1.5 | Cytoplasm | enzyme |
| HMOX1 | heme oxygenase 1 | 1.8 | Cytoplasm | enzyme |
| Ldh (complex) | | | Cytoplasm | complex |
| LDL | | | Plasma Membrane | complex |
| LOXL1 | lysyl oxidase like 1 | 1.7 | Extracellular Space | enzyme |
| MAFF | MAF bZIP transcription factor F | 1.9 | Nucleus | transcription regulator |
| MAFK | MAF bZIP transcription factor K | 1.6 | Nucleus | transcription regulator |
| NQO1 | NAD(P)H quinone dehydrogenase 1 | 1.6 | Cytoplasm | enzyme |
| OSBPL6 | oxysterol binding protein like 6 | 0.6 | Cytoplasm | other |
| PDLIM5 | PDZ and LIM domain 5 | 1.5 | Cytoplasm | other |
| PGD | phosphogluconate dehydrogenase | 1.6 | Cytoplasm | enzyme |
| PI3K (family) | | | Other | group |
| PLIN5 | perilipin 5 | 0.5 | Plasma Membrane | other |
| RDH | | | Cytoplasm | group |
| retinal dehydrogenase | | | Other | group |
| Sos | | | Cytoplasm | group |
| Srebp | | | Other | group |
| Tpm1 | tropomyosin 1. alpha | 0.6 | Plasma Membrane | other |
| TXNRD1 | thioredoxin reductase 1 | 1.6 | Cytoplasm | enzyme |

24 hrs of stretching, network 5

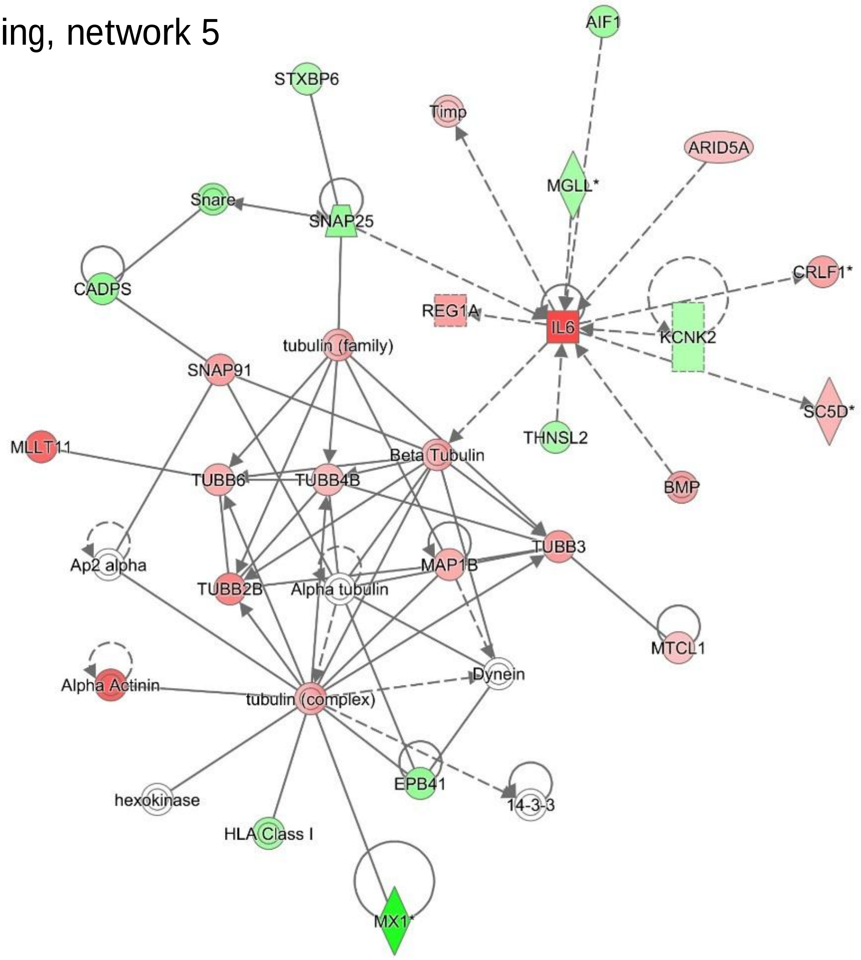


| Symbol | Gene Name | Fold change | Location | Family |
|---|---|---|---|---|
| 14-3-3 | | | Cytoplasm | group |
| AIF1 | allograft inflammatory factor 1 | 0.6 | Nucleus | other |
| Alpha Actinin | | | Cytoplasm | group |
| Alpha tubulin | | | Cytoplasm | group |
| Ap2 alpha | | | Plasma Membrane | group |
| ARID5A | AT-rich interaction domain 5A | 1.5 | Nucleus | transcription regulator |
| Beta Tubulin | | | Other | group |
| BMP | | | Extracellular Space | group |
| CADPS | calcium dependent secretion activator | 0.5 | Plasma Membrane | other |
| CRLF1 | cytokine receptor like factor 1 | 1.8 | Extracellular Space | other |
| Dynein | | | Cytoplasm | complex |
| EPB41 | erythrocyte membrane protein band 4.1 | 0.6 | Plasma Membrane | other |
| hexokinase | | | Other | group |
| HLA Class I | | | Plasma Membrane | complex |
| IL6 | interleukin 6 | 3.4 | Extracellular Space | cytokine |
| KCNK2 | potassium two pore domain channel subfamily K member 2 | 0.7 | Plasma Membrane | ion channel |
| MAP1B | microtubule associated protein 1B | 1.7 | Cytoplasm | other |
| MGLL | monoglyceride lipase | 0.6 | Plasma Membrane | enzyme |
| MLLT11 | myeloid/lymphoid or mixed-lineage leukemia; translocated to. 11 | 2.7 | Cytoplasm | other |
| MTCL1 | microtubule crosslinking factor 1 | 1.5 | Cytoplasm | other |
| MX1 | MX dynamin like GTPase 1 | 0.3 | Cytoplasm | enzyme |
| REG1A | regenerating family member 1 alpha | 1.9 | Extracellular Space | growth factor |
| SC5D | sterol-C5-desaturase | 1.6 | Cytoplasm | enzyme |
| SNAP25 | synaptosome associated protein 25 | 0.6 | Plasma Membrane | transporter |
| SNAP91 | synaptosome associated protein 91 | 1.9 | Plasma Membrane | other |
| Snare | | | Cytoplasm | complex |
| STXBP6 | syntaxin binding protein 6 | 0.7 | Cytoplasm | other |
| THNSL2 | threonine synthase like 2 | 0.6 | Other | other |
| Timp | | | Extracellular Space | group |
| TUBB2B | tubulin beta 2B class IIb | 2.3 | Cytoplasm | other |
| TUBB3 | tubulin beta 3 class III | 2.0 | Cytoplasm | other |
| TUBB4B | tubulin beta 4B class IVb | 1.6 | Cytoplasm | other |
| TUBB6 | tubulin beta 6 class V | 1.7 | Cytoplasm | other |
| tubulin (complex) | | | Cytoplasm | complex |
| tubulin (family) | | | Cytoplasm | group |

# 48 hrs of stretching, network 1

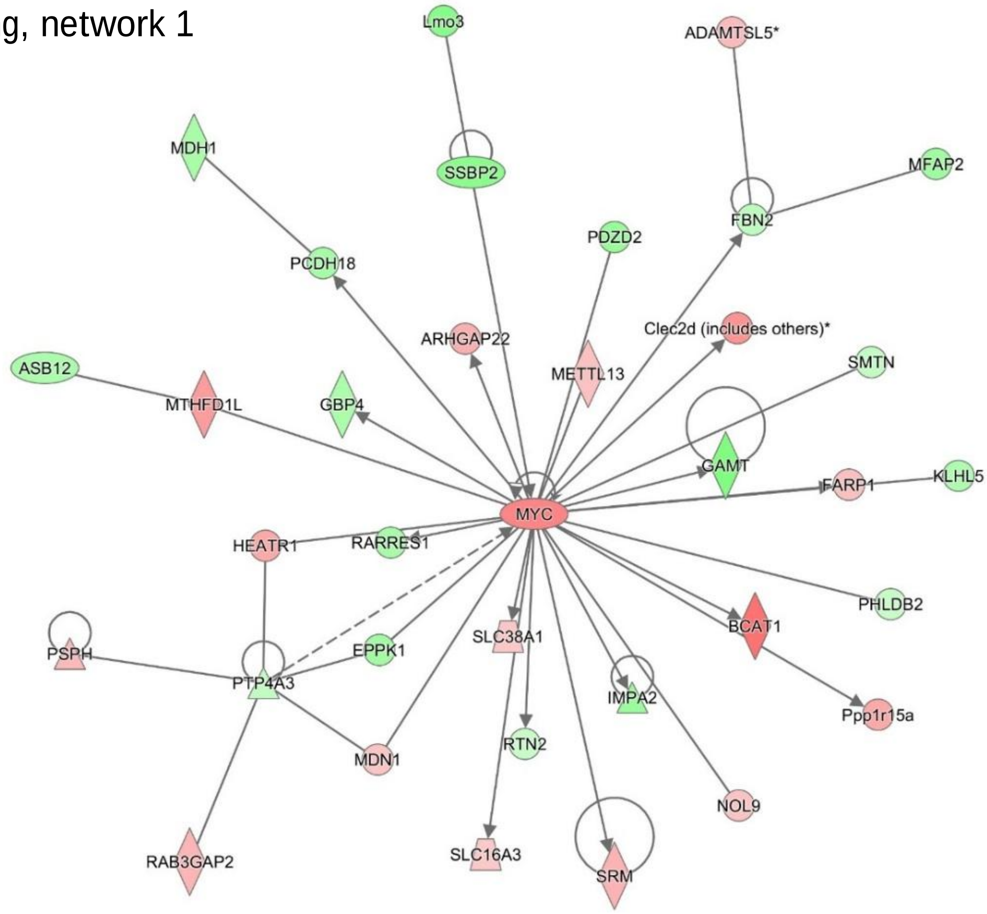


| Symbol | Gene Name | Fold change | Location | Family |
|---|---|---|---|---|
| ADAMTSL5 | ADAMTS like 5 | 1.7 | Extracellular Space | other |
| ARHGAP22 | Rho GTPase activating protein 22 | 1.8 | Cytoplasm | other |
| ASB12 | ankyrin repeat and SOCS box containing 12 | 0.5 | Nucleus | transcription regulator |
| BCAT1 | branched chain amino acid transaminase 1 | 3.0 | Cytoplasm | enzyme |
| Clec2d (includes others) | C-type lectin domain family 2. member D | 2.3 | Plasma Membrane | other |
| EPPK1 | epiplakin 1 | 0.5 | Cytoplasm | other |
| FARP1 | FERM. ARH/RhoGEF and pleckstrin domain protein 1 | 1.6 | Plasma Membrane | other |
| FBN2 | fibrillin 2 | 0.6 | Extracellular Space | other |
| GAMT | guanidinoacetate N-methyltransferase | 0.4 | Cytoplasm | enzyme |
| GBP4 | guanylate binding protein 4 | 0.5 | Cytoplasm | enzyme |
| HEATR1 | HEAT repeat containing 1 | 1.9 | Nucleus | other |
| IMPA2 | inositol monophosphatase 2 | 0.5 | Cytoplasm | phosphatase |
| KLHL5 | kelch like family member 5 | 0.5 | Extracellular Space | other |
| Lmo3 | LIM domain only 3 | 0.4 | Cytoplasm | other |
| MDH1 | malate dehydrogenase 1 | 0.5 | Cytoplasm | enzyme |
| MDN1 | midasin AAA ATPase 1 | 1.6 | Nucleus | other |
| METTL13 | methyltransferase like 13 | 1.6 | Other | enzyme |
| MFAP2 | microfibrillar associated protein 2 | 0.5 | Extracellular Space | other |
| MTHFD1L | methylenetetrahydrofolate dehydrogenase (NADP+ dependent) 1-like | 2.1 | Cytoplasm | enzyme |
| MYC | v-myc avian myelocytomatosis viral oncogene homolog | 2.6 | Nucleus | transcription regulator |
| NOL9 | nucleolar protein 9 | 1.6 | Nucleus | other |
| PCDH18 | protocadherin 18 | 0.5 | Extracellular Space | other |
| PDZD2 | PDZ domain containing 2 | 0.5 | Plasma Membrane | other |
| PHLDB2 | pleckstrin homology like domain family B member 2 | 0.7 | Cytoplasm | other |
| Ppp1r15a | protein phosphatase 1. regulatory subunit 15A | 1.9 | Cytoplasm | other |
| PSPH | phosphoserine phosphatase | 1.7 | Cytoplasm | phosphatase |
| PTP4A3 | protein tyrosine phosphatase type IVA. member 3 | 0.6 | Plasma Membrane | phosphatase |
| RAB3GAP2 | RAB3 GTPase activating non-catalytic protein subunit 2 | 1.8 | Cytoplasm | enzyme |
| RARRES1 | retinoic acid receptor responder 1 | 0.5 | Plasma Membrane | other |
| RTN2 | reticulon 2 | 0.7 | Cytoplasm | other |
| SLC16A3 | solute carrier family 16 member 3 | 1.5 | Plasma Membrane | transporter |
| SLC38A1 | solute carrier family 38 member 1 | 1.5 | Plasma Membrane | transporter |
| SMTN | smoothelin | 0.6 | Extracellular Space | other |
| SRM | spermidine synthase | 1.8 | Cytoplasm | enzyme |
| SSBP2 | single stranded DNA binding protein 2 | 0.4 | Nucleus | transcription regulator |

48 hrs of stretching, network 2

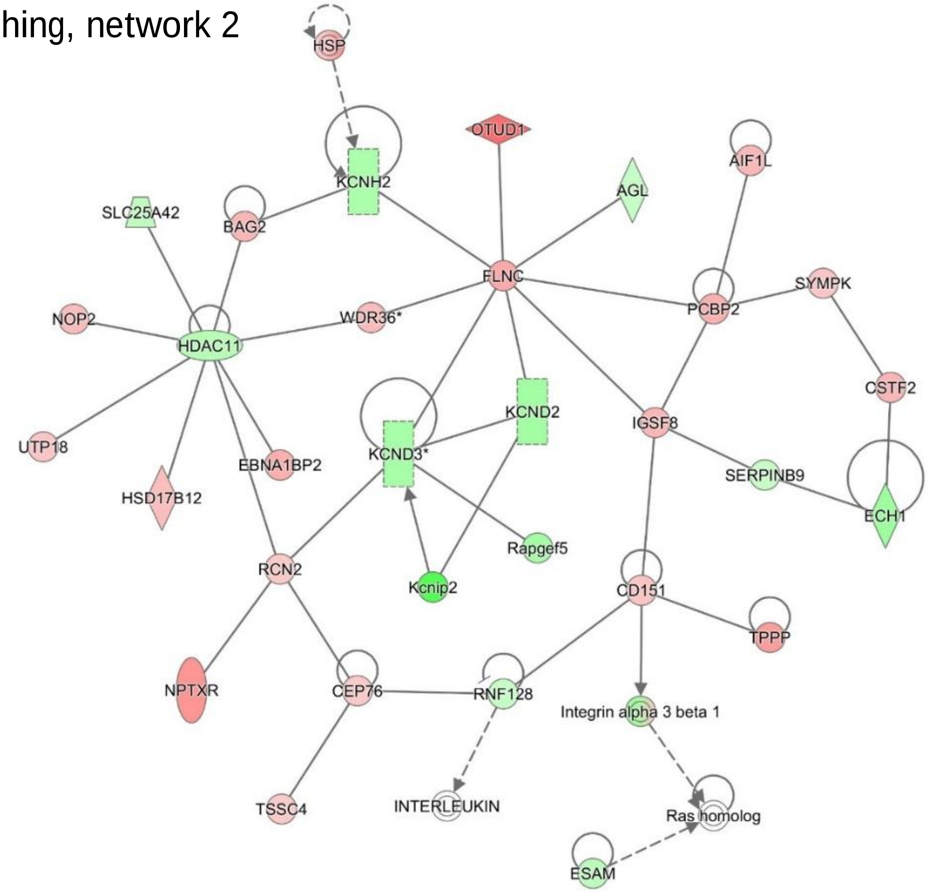


| Symbol | Gene Name | Fold change | Location | Family |
|---|---|---|---|---|
| AGL | amylo-alpha-1. 6-glucosidase. 4-alpha-glucanotransferase | 0.7 | Cytoplasm | enzyme |
| AIF1L | allograft inflammatory factor 1 like | 1.7 | Plasma Membrane | other |
| BAG2 | BCL2 associated athanogene 2 | 1.7 | Cytoplasm | other |
| CD151 | CD151 molecule (Raph blood group) | 1.6 | Plasma Membrane | other |
| CEP76 | centrosomal protein 76 | 1.5 | Cytoplasm | other |
| CSTF2 | cleavage stimulation factor subunit 2 | 1.8 | Nucleus | other |
| EBNA1BP2 | EBNA1 binding protein 2 | 1.8 | Nucleus | other |
| ECH1 | enoyl-CoA hydratase 1 | 0.5 | Cytoplasm | enzyme |
| ESAM | endothelial cell adhesion molecule | 0.6 | Plasma Membrane | other |
| FLNC | filamin C | 1.9 | Cytoplasm | other |
| HDAC11 | histone deacetylase 11 | 0.6 | Nucleus | transcription regulator |
| HSD17B12 | hydroxysteroid 17-beta dehydrogenase 12 | 1.7 | Cytoplasm | enzyme |
| HSP | | | Cytoplasm | group |
| IGSF8 | immunoglobulin superfamily member 8 | 1.7 | Plasma Membrane | other |
| Integrin alpha 3 beta 1 | | | Plasma Membrane | complex |
| INTERLEUKIN | | | Other | group |
| KCND2 | potassium voltage-gated channel subfamily D member 2 | 0.5 | Plasma Membrane | ion channel |
| KCND3 | potassium voltage-gated channel subfamily D member 3 | 0.5 | Plasma Membrane | ion channel |
| KCNH2 | potassium voltage-gated channel subfamily H member 2 | 0.5 | Plasma Membrane | ion channel |
| Kcnip2 | Kv channel-interacting protein 2 | 0.3 | Plasma Membrane | other |
| NOP2 | NOP2 nucleolar protein | 1.7 | Nucleus | other |
| NPTXR | neuronal pentraxin receptor | 2.3 | Plasma Membrane | transmembrane receptor |
| OTUD1 | OTU deubiquitinase 1 | 3.1 | Other | peptidase |
| PCBP2 | poly(rC) binding protein 2 | 1.9 | Nucleus | other |
| Rapgef5 | Rap guanine nucleotide exchange factor 5 | 0.5 | Nucleus | other |
| Ras homolog | | | Cytoplasm | group |
| RCN2 | reticulocalbin 2 | 1.5 | Cytoplasm | other |
| RNF128 | ring finger protein 128. E3 ubiquitin protein ligase | 0.7 | Cytoplasm | other |
| SERPINB9 | serpin family B member 9 | 0.7 | Cytoplasm | other |
| SLC25A42 | solute carrier family 25 member 42 | 0.6 | Cytoplasm | transporter |
| SYMPK | symplekin | 1.5 | Cytoplasm | other |
| TPPP | tubulin polymerization promoting protein | 2.1 | Cytoplasm | other |
| TSSC4 | tumor suppressing subtransferable candidate 4 | 1.5 | Other | other |
| UTP18 | UTP18. small subunit processome component | 1.5 | Nucleus | other |
| WDR36 | WD repeat domain 36 | 1.7 | Extracellular Space | other |

48 hrs of stretching, network 3

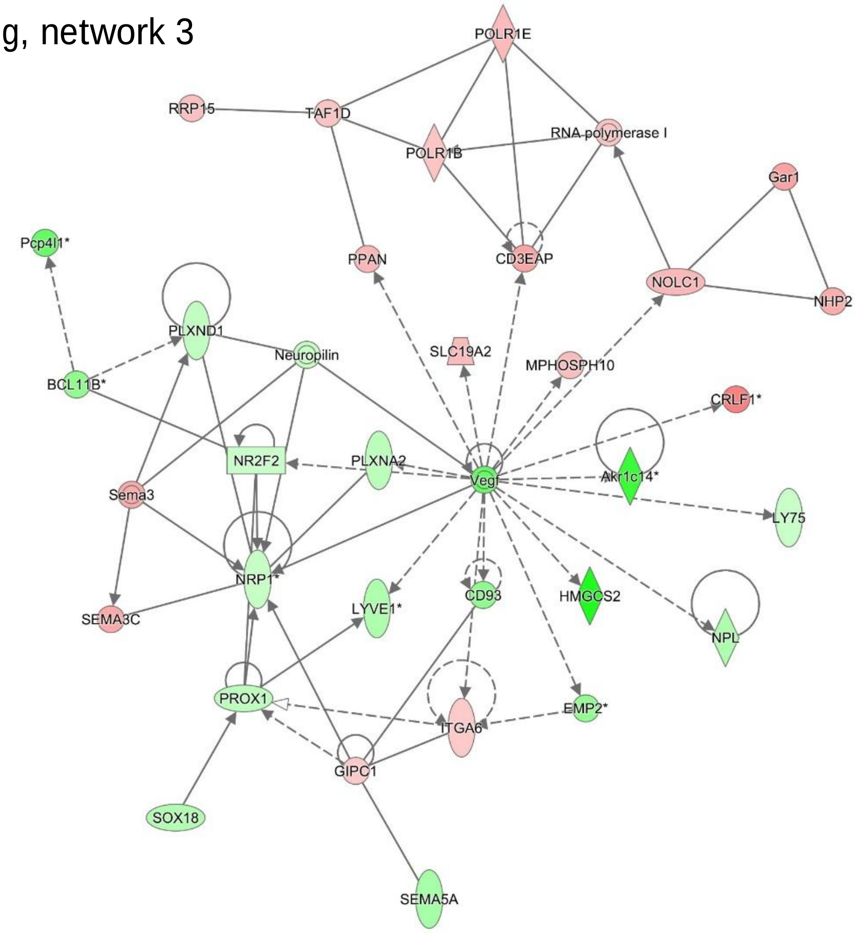


| Symbol | Gene Name | Fold change | Location | Family |
|---|---|---|---|---|
| Akr1c14 | aldo-keto reductase family 1. member C14 | 0.2 | Cytoplasm | enzyme |
| BCL11B | B-cell CLL/lymphoma 11B | 0.4 | Nucleus | other |
| CD3EAP | CD3e molecule associated protein | 2.0 | Nucleus | other |
| CD93 | CD93 molecule | 0.4 | Plasma Membrane | other |
| CRLF1 | cytokine receptor like factor 1 | 2.7 | Extracellular Space | other |
| EMP2 | epithelial membrane protein 2 | 0.4 | Plasma Membrane | other |
| Gar1 | GAR1 ribonucleoprotein | 2.0 | Nucleus | other |
| GIPC1 | GIPC PDZ domain containing family member 1 | 1.5 | Cytoplasm | other |
| HMGCS2 | 3-hydroxy-3-methylglutaryl-CoA synthase 2 | 0.2 | Cytoplasm | enzyme |
| ITGA6 | integrin subunit alpha 6 | 1.5 | Plasma Membrane | transmembrane receptor |
| LY75 | lymphocyte antigen 75 | 0.7 | Plasma Membrane | transmembrane receptor |
| LYVE1 | lymphatic vessel endothelial hyaluronan receptor 1 | 0.5 | Plasma Membrane | transmembrane receptor |
| MPHOSPH10 | M-phase phosphoprotein 10 | 1.7 | Nucleus | other |
| Neuropilin | | | Plasma Membrane | group |
| NHP2 | NHP2 ribonucleoprotein | 1.9 | Nucleus | other |
| NOLC1 | nucleolar and coiled-body phosphoprotein 1 | 1.7 | Nucleus | transcription regulator |
| NPL | N-acetylneuraminate pyruvate lyase | 0.5 | Cytoplasm | enzyme |
| NR2F2 | nuclear receptor subfamily 2 group F member 2 | 0.7 | Nucleus | ligand-dependent nuclear receptor |
| NRP1 | neuropilin 1 | 0.7 | Plasma Membrane | transmembrane receptor |
| Pcp4l1 | Purkinje cell protein 4-like 1 | 0.3 | Other | other |
| PLXNA2 | plexin A2 | 0.6 | Plasma Membrane | transmembrane receptor |
| PLXND1 | plexin D1 | 0.6 | Plasma Membrane | transmembrane receptor |
| POLR1B | RNA polymerase I subunit B | 1.6 | Nucleus | enzyme |
| POLR1E | RNA polymerase I subunit E | 1.7 | Nucleus | enzyme |
| PPAN | peter pan homolog (Drosophila) | 1.8 | Nucleus | other |
| PROX1 | prospero homeobox 1 | 0.6 | Nucleus | transcription regulator |
| RNA polymerase I | | | Nucleus | complex |
| RRP15 | ribosomal RNA processing 15 homolog | 1.6 | Nucleus | other |
| Sema3 | | | Extracellular Space | group |
| SEMA3C | semaphorin 3C | 1.9 | Extracellular Space | other |
| SEMA5A | semaphorin 5A | 0.5 | Plasma Membrane | transmembrane receptor |
| SLC19A2 | solute carrier family 19 member 2 | 1.7 | Plasma Membrane | transporter |
| SOX18 | SRY-box 18 | 0.6 | Nucleus | transcription regulator |
| TAF1D | TATA-box binding protein associated factor. RNA polymerase I subunit D | 1.6 | Nucleus | other |
| Vegf | | | Extracellular Space | group |

48 hrs of stretching, network 4

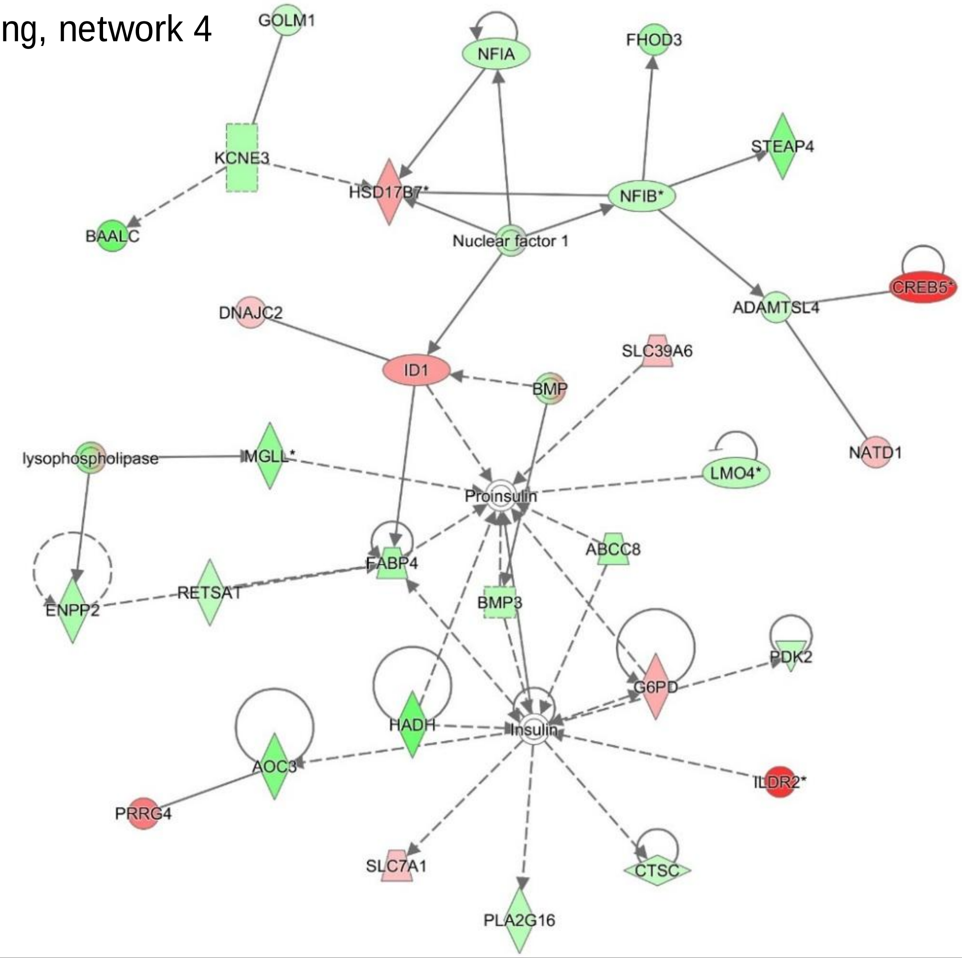


| Symbol | Gene Name | Fold change | Location | Family |
|---|---|---|---|---|
| ABCC8 | ATP binding cassette subfamily C member 8 | 0.5 | Plasma Membrane | transporter |
| ADAMTSL4 | ADAMTS like 4 | 0.7 | Extracellular Space | other |
| AOC3 | amine oxidase. copper containing 3 | 0.4 | Plasma Membrane | enzyme |
| BAALC | brain and acute leukemia. cytoplasmic | 0.3 | Cytoplasm | other |
| BMP | | | Extracellular Space | group |
| BMP3 | bone morphogenetic protein 3 | 0.6 | Extracellular Space | growth factor |
| CREB5 | cAMP responsive element binding protein 5 | 4.9 | Nucleus | transcription regulator |
| CTSC | cathepsin C | 0.6 | Cytoplasm | peptidase |
| DNAJC2 | DnaJ heat shock protein family (Hsp40) member C2 | 1.5 | Nucleus | other |
| ENPP2 | ectonucleotide pyrophosphatase/phosphodiesterase 2 | 0.5 | Plasma Membrane | enzyme |
| FABP4 | fatty acid binding protein 4 | 0.5 | Cytoplasm | transporter |
| FHOD3 | formin homology 2 domain containing 3 | 0.5 | Nucleus | other |
| G6PD | glucose-6-phosphate dehydrogenase | 1.9 | Cytoplasm | enzyme |
| GOLM1 | golgi membrane protein 1 | 0.7 | Cytoplasm | other |
| HADH | hydroxyacyl-CoA dehydrogenase | 0.3 | Cytoplasm | enzyme |
| HSD17B7 | hydroxysteroid 17-beta dehydrogenase 7 | 2.1 | Cytoplasm | enzyme |
| ID1 | inhibitor of DNA binding 1. HLH protein | 2.2 | Nucleus | transcription regulator |
| ILDR2 | immunoglobulin like domain containing receptor 2 | 7.1 | Other | other |
| Insulin | | | Extracellular Space | group |
| KCNE3 | potassium voltage-gated channel subfamily E regulatory subunit 3 | 0.5 | Plasma Membrane | ion channel |
| LMO4 | LIM domain only 4 | 0.6 | Nucleus | transcription regulator |
| lysophospholipase | | | Other | group |
| MGLL | monoglyceride lipase | 0.4 | Plasma Membrane | enzyme |
| NATD1 | N-acetyltransferase domain containing 1 | 1.7 | Other | other |
| NFIA | nuclear factor I A | 0.6 | Nucleus | transcription regulator |
| NFIB | nuclear factor I B | 0.6 | Nucleus | transcription regulator |
| Nuclear factor 1 | | | Nucleus | group |
| PDK2 | pyruvate dehydrogenase kinase 2 | 0.6 | Cytoplasm | kinase |
| PLA2G16 | phospholipase A2 group XVI | 0.6 | Nucleus | enzyme |
| Proinsulin | | | Other | group |
| PRRG4 | proline rich and Gla domain 4 | 2.8 | Plasma Membrane | other |
| RETSAT | retinol saturase | 0.6 | Cytoplasm | enzyme |
| SLC39A6 | solute carrier family 39 member 6 | 1.7 | Plasma Membrane | transporter |
| SLC7A1 | solute carrier family 7 member 1 | 1.6 | Plasma Membrane | transporter |

48 hrs of stretching, network 5

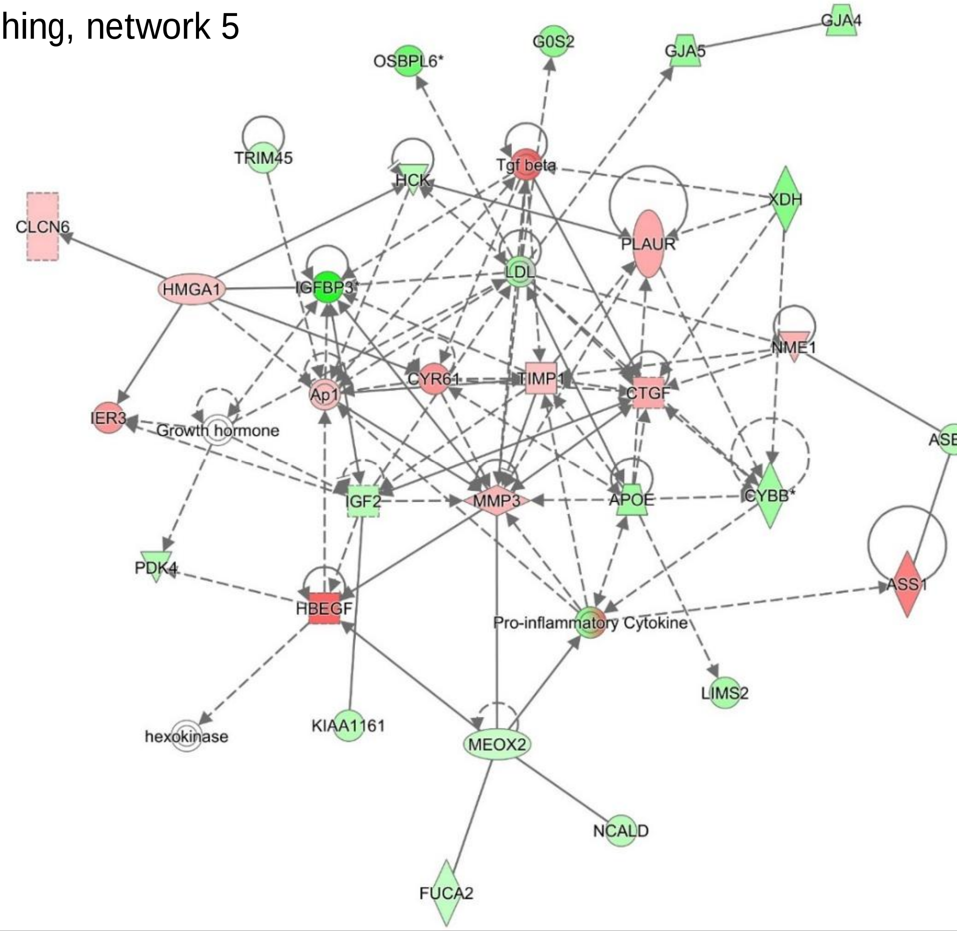


| Symbol | Gene Name | Fold change | Location | Family |
|---|---|---|---|---|
| Ap1 | Ap! Transcription factor | | Nucleus | complex |
| APOE | apolipoprotein E | 0.5 | Extracellular Space | transporter |
| ASB18 | ankyrin repeat and SOCS box containing 18 | 0.6 | Other | other |
| ASS1 | argininosuccinate synthase 1 | 2.7 | Cytoplasm | enzyme |
| CLCN6 | chloride voltage-gated channel 6 | 1.5 | Plasma Membrane | ion channel |
| CTGF | connective tissue growth factor | 1.9 | Extracellular Space | growth factor |
| CYBB | cytochrome b-245 beta chain | 0.5 | Cytoplasm | enzyme |
| CYR61 | cysteine rich angiogenic inducer 61 | 2.3 | Extracellular Space | other |
| FUCA2 | fucosidase. alpha-L- 2. plasma | 0.6 | Extracellular Space | enzyme |
| G0S2 | G0/G1 switch 2 | 0.4 | Cytoplasm | other |
| GJA4 | gap junction protein alpha 4 | 0.5 | Plasma Membrane | transporter |
| GJA5 | gap junction protein alpha 5 | 0.5 | Plasma Membrane | transporter |
| Growth hormone | | | Extracellular Space | group |
| HBEGF | heparin binding EGF like growth factor | 3.3 | Extracellular Space | growth factor |
| HCK | HCK proto-oncogene. Src family tyrosine kinase | 0.6 | Cytoplasm | kinase |
| hexokinase | | | Other | group |
| HMGA1 | high mobility group AT-hook 1 | 1.6 | Nucleus | transcription regulator |
| IER3 | immediate early response 3 | 2.2 | Cytoplasm | other |
| IGF2 | insulin like growth factor 2 | 0.6 | Extracellular Space | growth factor |
| IGFBP3 | insulin like growth factor binding protein 3 | 0.2 | Extracellular Space | other |
| KIAA1161 | KIAA1161 | 0.6 | Nucleus | other |
| LDL | | | Plasma Membrane | complex |
| LIMS2 | LIM zinc finger domain containing 2 | 0.5 | Cytoplasm | other |
| MEOX2 | mesenchyme homeobox 2 | 0.7 | Nucleus | transcription regulator |
| MMP3 | matrix metallopeptidase 3 | 1.8 | Extracellular Space | peptidase |
| NCALD | neurocalcin delta | 0.6 | Cytoplasm | other |
| NME1 | NME/NM23 nucleoside diphosphate kinase 1 | 1.8 | Cytoplasm | kinase |
| OSBPL6 | oxysterol binding protein like 6 | 0.3 | Cytoplasm | other |
| PDK4 | pyruvate dehydrogenase kinase 4 | 0.6 | Cytoplasm | kinase |
| PLAUR | plasminogen activator. urokinase receptor | 1.9 | Plasma Membrane | transmembrane receptor |
| Pro-inflammatory Cytokine | | | Other | group |
| Tgf beta | | | Extracellular Space | group |
| TIMP1 | TIMP metallopeptidase inhibitor 1 | 1.6 | Extracellular Space | cytokine |
| TRIM45 | tripartite motif containing 45 | 0.6 | Cytoplasm | other |
| XDH | xanthine dehydrogenase | 0.4 | Cytoplasm | enzyme |
